# Complexity of *cis*-regulatory organization of *six3a *during forebrain and eye development in zebrafish

**DOI:** 10.1186/1471-213X-10-35

**Published:** 2010-03-26

**Authors:** Chung-Hao Chao, Horng-Dar Wang, Chiou-Hwa Yuh

**Affiliations:** 1 Division of Molecular and Genomic Medicine, National Health Research Institutes, Zhunan Town, Miaoli County, Taiwan; 2 College of Life Science and Institute of Biotechnology, National Tsing-Hua University, HsinChu, Taiwan; 3 College of Life Science and Institute of Bioinformatics and Structural Biology, National Tsing-Hua University, HsinChu, Taiwan; 4 Department of Biological Science & Technology, National Chiao Tung University, HsinChu, Taiwan

## Abstract

**Background:**

Six3a belongs to the SIX family of homeodomain proteins and is expressed in the most anterior neural plate at the beginning of neurogenesis in various species. Though the function of Six3a as a crucial regulator of eye and forebrain development has been thoroughly investigated, the transcriptional regulation of *six3a *is not well understood.

**Results:**

To elucidate the transcriptional regulation of *six3a*, we performed an *in vivo *reporter assay. Alignment of the 21-kb region surrounding the zebrafish *six3a *gene with the analogous region from different species identified several conserved non-coding modules. Transgenesis in zebrafish identified two enhancer elements and one suppressor. The D module drives the GFP reporter in the forebrain and eyes at an early stage, while the A module is responsible for the later expression. The A module also works as a repressor suppressing ectopic expression from the D module. Mutational analysis further minimized the A module to four highly conserved elements and the D module to three elements. Using electrophoresis mobility shift assays, we also provided evidence for the presence of DNA-binding proteins in embryonic nuclear extracts. The transcription factors that may occupy those highly conserved elements were also predicted.

**Conclusion:**

This study provides a comprehensive view of *six3a *transcription regulation during brain and eye development and offers an opportunity to establish the gene regulatory networks underlying neurogenesis in zebrafish.

## Background

Zebrafish (*Danio rerio*) has long been an excellent vertebrate model organism for developmental biology [1-3] and is used to study the mechanisms of axis formation [4], endoderm differentiation [3,5] and muscle development [6]. It was also recently suggested to be an excellent model system for eye genetics [7]. Development is controlled by the hierarchical regulation between signaling pathways and transcription factors. The basic operating principle for specifying a territory or regulatory state is controlled coordinately by transcription factors and signal transduction machinery in the *cis*-regulatory code. Gene regulatory networks (GRNs) for early embryogenesis have been established in sea urchin mesendoderm [8], *Xenopus *endoderm [9], *Drosophila *dorsal/ventral polarity [10] and zebrafish mesendoderm [11]. It is essential to decode the *cis*-regulatory operation for the key transcription regulators contributing to the GRNs to elucidate the development of tissues and organs.

The Sine Oculis Homeobox (SIX) proteins share two evolutionarily conserved functional motifs in which the 115 amino acid SIX protein-protein interaction domain is located just upstream of the homeobox DNA binding domain [12,13]. Disrupting the SIX domain or the homeodomain abolishes the ability of Six3a to induce rostral forebrain enlargement in zebrafish, implying that these domains are essential for *six3 *gene function [14]. However, it also has been suggested that part of the biochemical and functional specificity between members of the SIX protein family is due to their non-conserved C-terminal segments [15].

Six3 is a member of the SIX family and is expressed in the most rostral portion of the brain in many animals. The first member of the SIX family, *sine oculis *(*so*), was identified in *Drosophila *[16], and later, six3 was discovered in many other species, including mouse [16], chick [17], zebrafish [14], medaka [18] and *Xenopus *[19]. Together with the products of other homeobox genes, such as Otx [20] and Emx [21], Six3 plays a central role in the patterning of forebrain and eye development [22-25]. The function of Six3a in the development of the forebrain and eyes has been demonstrated in many species. Overexpression of *six3 *induces rostral forebrain enlargement in zebrafish and promotes ectopic lens formation [14] and ectopic retinal primordia formation in medaka [26] and *Xenopus *[27]. The telencephalon of *six3a *and *six3b *double morphant embryos is markedly reduced in size, owing to impaired cell proliferation [28]. In humans, study of severe malformation of the brain identified that mutations in the homeodomain of the Six3 gene may relate to holoprosencephaly [29-31]. Therefore, previous studies suggest a conserved function for Six3 in eye and forebrain development in metazoans.

The transcriptional regulation of *six3a *has been investigated previously (e.g., *olSix3.2 *in the developing medaka forebrain [32] and *six3.1 *in zebrafish retina and forebrain [33]). However, the minimal binding elements and the transcription factors were not identified in those studies. The negative regulator for forebrain specification, LOM4b, has also been found [25], but how it regulates *six3a *in terms of binding sites is not known. Several eye-field transcription factors (EFTFs) are expressed in a dynamic, overlapping pattern in the presumptive eye and forebrain. A genetic network regulating vertebrate eye field specification has been proposed in *Xenopus *using a combination of subsets of (EFTFs) and functional (inductive) analysis [34].

To delineate the network of *trans*-acting factors that control the evolutionarily conserved activity of *six3a *during forebrain development, we studied the function of the conserved non-coding regions in zebrafish *six3a*. Functionally important regions in the genome usually evolve more slowly than non-functional regions due to selective pressure. Alignment of conserved non-coding DNA sequences among different species using bioinformatics tools (e.g., the UCSC genome browser) provides a useful method for identifying the important regulatory elements of a gene (Chen and Blanchette, 2007; Werner et al., 2007). To facilitate the identification of functionally important elements in *six3a*, we applied the same strategy as for conserved non-coding regions. We took advantage of the power of computational analysis, the availability of the zebrafish genome sequence, and the efficiency of embryo transgenesis to analyze the regulatory control of one of the two *six3 *homologs in zebrafish, *six3a*. Elucidating the regulation of *six3a *and identifying the transcription factors responsible for correct expression of *six3a *helps to show how the gene is regulated and reveals the comprehensive gene regulatory networks (GRNs) for forebrain and eye specification across all vertebrates.

## Results

### Identification of the *cis*-regulatory modules for *six3a *expression in zebrafish

Both medaka and zebrafish have Six3 duplications. We aligned 14 protein sequences for the Six3 homologs (Additional file 1) and performed phylogenetic analysis (Additional file 2). From the protein sequence comparison, we found that zebrafish Six3a is more similar to medaka Six3.2 (85% identity) than to Six3.1 (75% identity). From the phylogenetic analysis, medaka Six3.2 and zebrafish Six3a were the closest homologs, while medaka Six3.1 was more closely related to zebrafish Six3b.

**Figure 1:**
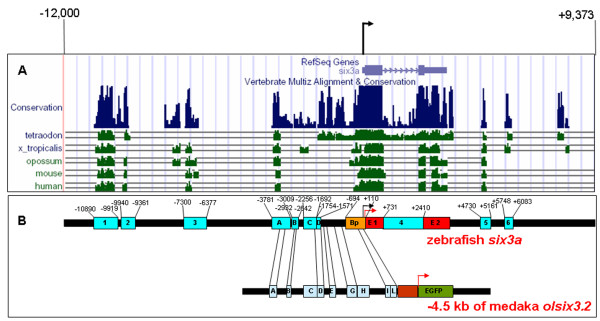
**Zebrafish *six3a *conserved non-coding modules and map**. (A) The 21-kb genomic region from -12 kb to +9373 bp, relative to the zebrafish *six3a *transcriptional start site. The horizontal light blue blocks at the top of the figure represent two exons of zebrafish *six3a*, the transcriptional start site indicated as a black arrow. Green histograms indicate the sequence similarities of different species in the corresponding regions. The conserved regions are shown as dark blue histograms under the *six3a *transcript. (B) Schematic structure of zebrafish *six3a cis*-regulatory elements. The thick black line represents the genomic sequence from -12 kb to +9937 bp. The horizontal red blocks at the top of the figure represent two exons of zebrafish *six3a*, and "+1" represents the transcriptional start site indicated as a black arrow. The translational initiation site is indicated as a red arrow. Conserved regions in Fig. 1A are shown by light blue blocks named, from 5' to 3': 1, 2, 3, A, B, C, D, 4, 5 and 6. Bp represents the basal promoter, shown by an orange block. Modules 1, A and 5 are conserved among all species, modules 2 and 3 show minor conservation and the remaining modules have low sequence similarity among species. The 4.5-kb upstream sequence from medaka *olsix3.2 *was used as comparison.

To identify the *cis*-regulatory modules responsible for zebrafish *six3a *expression, we first aligned 21 kb of DNA (spanning from -12 kb to +9 kb of the zebrafish *six3a *transcription initiation site) with the corresponding sequences from *Tetraodon*, *Xenopus tropicalis*, opossum, mouse and human. We used the 2006 version of the UCSC genome browser to identify conserved regions. If conservation appeared in more than four species, it was regarded as highly conserved, and if conservation was seen in less than two species, it was regarded as less conserved. Excluding the *six3a *coding region (two exons labeled E1 and E2 in red boxes), ten conserved non-coding modules (blue boxes) and the basal promoter (orange box) were identified (Fig. 1A). The transcriptional regulation of the *six3a *homolog in medaka (*olSix3.2*) was investigated previously by Conte and Bovolenta [32], who analyzed the 4.5-kb upstream sequence from *olsix3.2 *[32]. For ease and consistency, we used the same nomenclature for the modules that have equivalent positions as *olsix3.2*. Therefore, we have modules A through D, which were similar to Box A through D in the work of Conte et al. We have included the medaka sequence for comparison in Fig. 1B. The basal promoter (Bp) actually overlapped with Box I and L of *olsix3.2*. Box E and G of *olsix3.2 *matched to the sequence of *zsix3a*, but were not among our conserved modules; Box C and H of *olsix3.2 *did not match the *zsix3a *sequence. We illustrated the relationship of the genomic sequence of medaka *olsix3.2 *verses zebrafish *zsix3a *in Fig. 1B. To avoid confusion, we used different nomenclature for the modules identified through the UCSC genome browser cross-species comparison. Modules 1, 2, and 3 are located upstream from module A; module 4 is located in the intronic region; and modules 5 and 6 are in the downstream area. Modules B and C were not highly conserved. However, we tested their function because of a previous study by Anders Fjose's lab that demonstrated that a possible Pax6.1 binding site on module F and a putative Brn3b binding site on module E are important for regulating *six3a *[33].

### Brn3 and Pax6.1 binding sites on modules B and C contain no enhancer function in the forebrain or eyes up to 24 hpf

**Figure 2:**
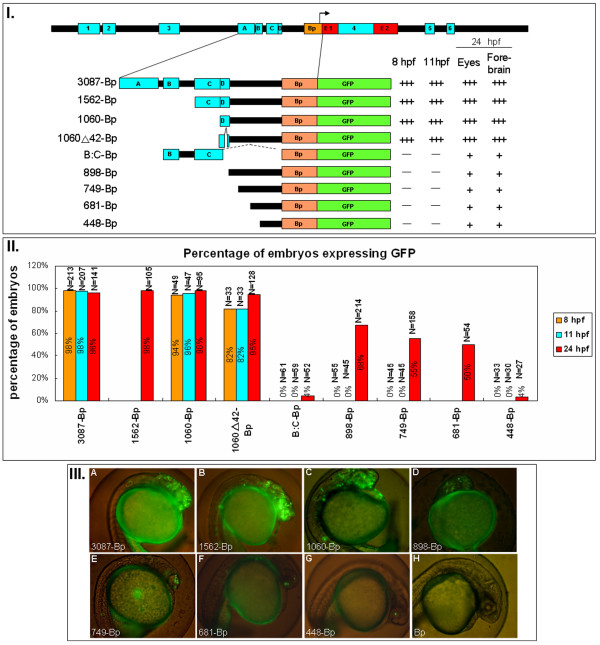
***six3a *promoter deletion mutant constructs**. **I**. Different deletion constructs used to study the combinatory effect of those modules. The names of those constructs are given by the length of each DNA fragments ligated to Bp. Followed each construct are the summary of the GFP expression level after microinjection into zebrafish embryos. Zebrafish embryos injected with each construct expressing GFP at different times (8, 11 and 24 hpf) and regions (eyes, forebrain and ectopic) are labeled "+", "++" and "+++" to indicate different levels of GFP intensity. The symbol "-" indicates the absence of GFP expression. **II**. The percentages of embryos expressing GFP from different batches are shown in the bar graph. The total numbers of embryos are indicated as N=number above each bar. Three different time points were shown: 8 hpf (orange), 11 hpf (blue) and 24 hpf (red). **III**. Representative GFP expression patterns for each construct. (A) 3087-Bp-GFP shows accurate and strong GFP expression in the forebrain and eyes. (B) 1562-Bp-GFP shows a similar expression pattern to 3087-Bp, with minor ectopic expression on the ventral side. (C) 1060-Bp-GFP shows similar expression and strong ectopic expression on the ventral side. (D) 898-Bp-GFP shows weak expression in the eyes and forebrain. (E) 749-Bp-GFP shows GFP expression in the eyes. (F) 681-Bp-GFP has GFP expression extending to the eye. (G) 448-Bp has very weak GFP expression in the eyes. (H) Bp-GFP, as a control, shows almost no expression of GFP, except for 1-2 cells in the forebrain region.

**Figure 3:**
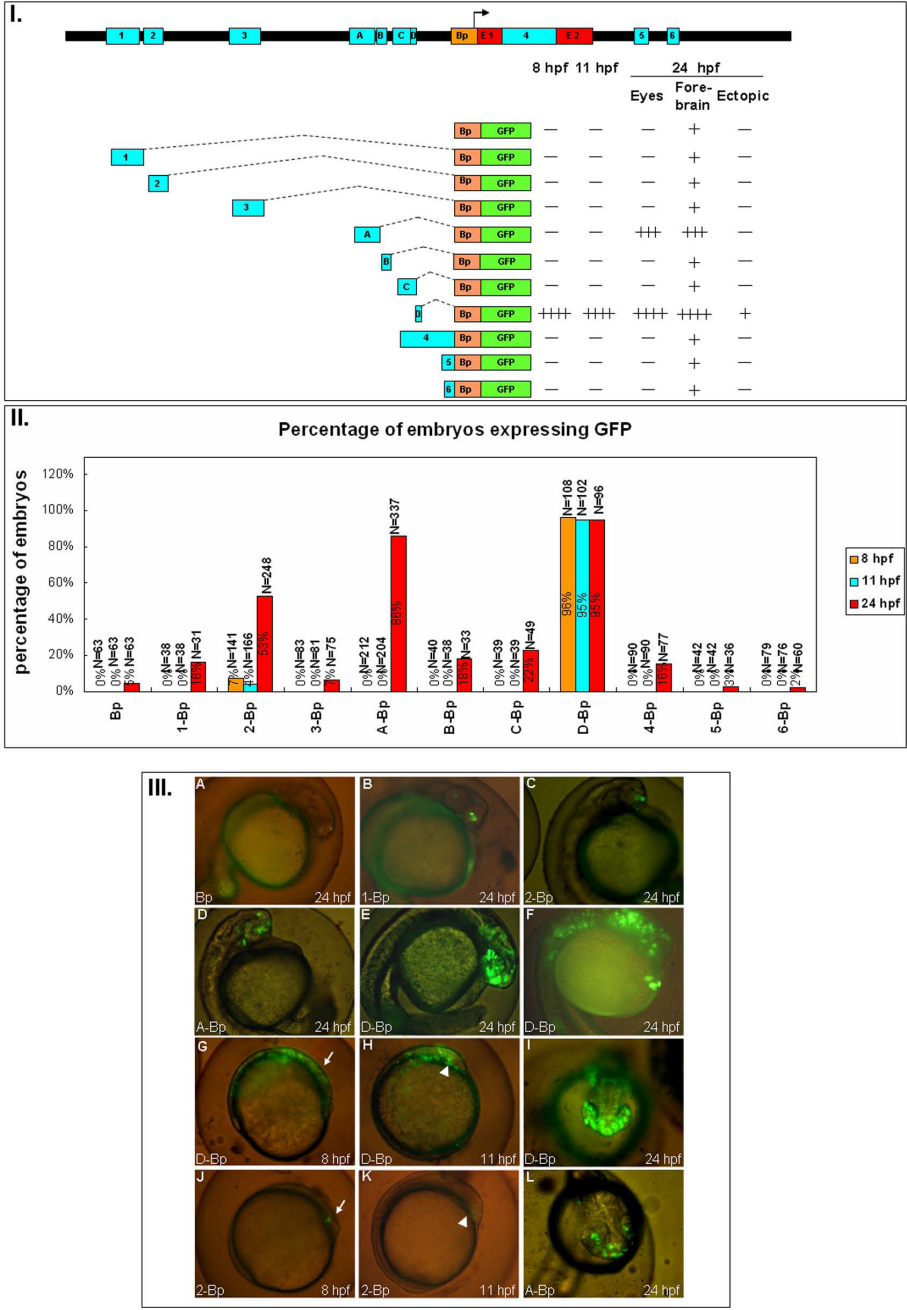
**The expression pattern of the ten conserved non-coding modules of zebrafish *six3a***. **I**. Different constructs used to study the function of each module. Followed by each construct are the summary of the GFP expression level. Zebrafish embryos injected with each construct expressing GFP at different times (8, 11 and 24 hpf) and regions (eyes, forebrain and ectopic), as in figure 2, the plus and minus indicated the level of GFP intensity. **II**. The percentages of embryos expressing GFP from different batches are shown with the total numbers of embryos, and three time points: 8 hpf (orange), 11 hpf (blue) and 24 hpf (red). **III**. Representative GFP expression patterns for each construct in lateral views (A-F, G-H, J-K) or dorsal view (I, L). Most of the images are from 24 hpf except for G and J at 8 hpf, and H and K at 11 hpf (A) Bp-GFP. (B) 1-Bp-GFP. (C) 2-Bp-GFP. (D) A-Bp-GFP. (E) D-Bp-GFP expresses GFP in forebrain and midbrain. (F) D-Bp-GFP expresses GFP extending to the notochord. (G) D-Bp-GFP expression pattern at 8 hpf; the anterior position is marked with an arrow. (H) D-Bp-GFP expression pattern at 11 hpf; the position of the brain is marked with an arrowhead. (I) D-Bp-GFP at 24 hpf showing ventral expression in addition to the forebrain and eye. (J) 2-Bp-GFP expresses at 8 hpf; the anterior position is marked with an arrow. (K) 2-Bp-GFP expression pattern at 11 hpf; the position of the brain is marked with an arrowhead. (L) A-Bp-GFP.

**Figure 4:**
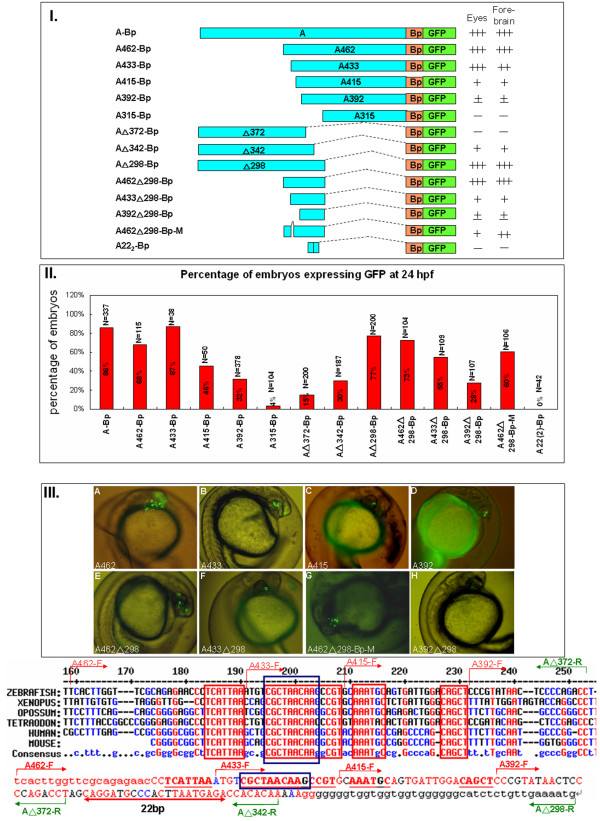
**Constructs and representative images for module A**. **I**. Different constructs used to study the function of *six3a *module A. The names of those constructs were given by the length of the DNA fragment left after 5' or 3' deletion. The number after A indicates the length of module A left after 5' deletion. The small triangle indicates the length of module A left after 3' deletion. Followed by each construct are the summary of the GFP expression level at 24 hpf for different regions (eyes or forebrain) as plus sign (+) to indicate different levels of GFP. The minus symbol (-) indicates the absence of GFP expression, and plus/minus (+/-) indicates slightly higher GFP expression than no expression. **II**. The percentages of embryos expressing GFP from different batches are shown with the total numbers of embryos from 24 hpf (red). **III**. Representative GFP expression patterns for each construct in lateral views at 24 hpf. (A) A462-GFP. (B) A433-Bp-GFP. (C) A415-Bp-GFP. (D) A392-Bp-GFP. (E) A462(triangle)298-Bp-GFP (F) A433(triangle)298-Bp-GFP. (G) A462(triangle)298-Bp-M-GFP. (H) A392(triangle)298-Bp-GFP. The last panel shows the sequence in module A. Conserved sequences in module A were aligned among zebrafish, *X. tropicalis*, opossum, *Tetraodon*, human and mouse. The primers used to obtain serial deletion constructs are shown in red or green arrow with direction indicated forward or reverse. The 22-bp region (underlines with double red arrow) was deleted in A462(triangle)298-BP-M-GFP. The red boxes represent the highly conserved elements.

**Figure 5:**
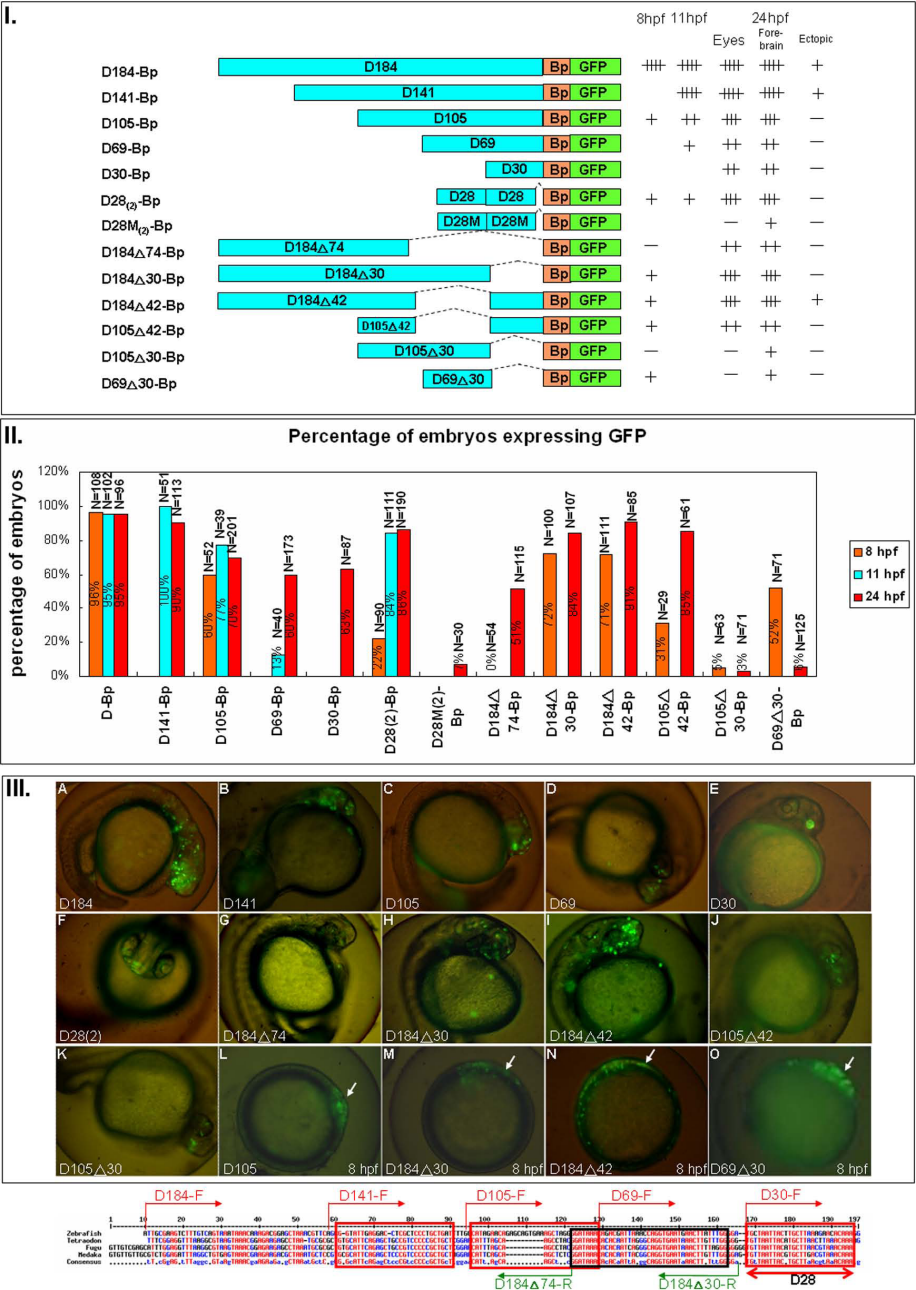
**Constructs and representative images for module D**. **I**. Different constructs used to study the function of *six3a *module D. The names of those constructs were given by the length of the DNA fragment left after 5' or 3' deletion. The number after D indicates the length of module D left after 5' deletion. The small triangle indicates the length of module D left after 3' deletion. Followed by each construct are the summary of the GFP expression level at different times (8, 11 and 24 hpf) and regions (eyes, forebrain and ectopic) as plus sign (+) to indicate different levels of GFP. The minus sign (-) indicates the absence of GFP expression. **II**. The percentages of embryos expressing GFP from different batches are shown with the total numbers of embryos, and three time points: 8 hpf (orange), 11 hpf (blue) and 24 hpf (red). **III**. Representative GFP expression patterns for each construct in lateral views at 24 hpf (A-K) or 8 hpf (L-O) with the anterior position marked with an arrow. (A) D184-GFP. (B) D141-Bp-GFP. (C) D105-Bp-GFP. (D) D69-Bp-GFP. (E) D30-Bp-GFP. (F) Dorsal view of D28(2)-Bp-GFP. (G) D184(triangle)74-Bp-GFP. (H) D184(triangle)30-Bp-GFP. (I) D184(triangle)42-Bp. (J) D105(triangle)42-Bp-GFP. (K) D105(triangle)30-Bp-GFP. (L) D105, (M) D184, (N) D184(triangle)42 and (O) D69(triangle)30. The last panel shows the sequence in module D. Conserved sequences in module D were aligned among zebrafish, *Tetraodon*, fugu and medaka, and the primers for making the deletion constructs are shown above the sequences. See legend in Fig 4 for others symbols.

To determine if the expression of zebrafish *six3a *is regulated by these conserved modules, a zebrafish *six3a *BAC clone (DKEY-254J21) was purchased from the BACPAC Resource Center (BPRC) and used as a template for PCR. Each module was PCR amplified from *six3a *BAC DNA using specific forward and reverse primers, and cloned into the pEGFPN1 vector to generate different GFP reporter constructs (Fig. 2-I, Fig. 3-I, Fig. 4-I and Fig. 5-I). The primers for amplifying those modules are listed in Additional file 3, and the sequence of each module is shown in Additional file 4. The GFP expression reporter constructs were individually injected into one-cell stage zebrafish embryos, and GFP expression patterns and images were captured at 8, 11 and 24 hpf. We report GFP expression in transiently transgenic embryos by two different criteria. The number of cells that express GFP per embryo is indicated by plus (+) and minus (-) signs (Fig. 2-I, Fig. 3-I, Fig. 4-I and Fig. 5-I). The percentages of embryos that expressed GFP are shown in bar graphs (Fig. 2-II, Fig. 3-II, Fig. 4-II and Fig. 5-II). Representative images are shown for each construct (Fig. 2-III, Fig. 3-III, Fig. 4-III and Fig. 5-III).

We first performed deletion analysis to search for the important elements. The genomic sequence spanning from -3782 to +110, containing modules A-D and Bp, was fused with GFP (Fig. 2-I). Transgenesis analysis showed that this 4-kb fragment (3087-Bp) expressed GFP accurately at all stages (Fig. 2-II-A). This is similar to the results by Bovolenta in medaka *six3.2*, in which the 4.5-kb fragment was responsible for *six3.2 *expression. Serial deletion mutants demonstrated that the genomic sequence that started from module D (1060-Bp, Fig. 3-II-C) exhibited a strong expression pattern similar to the 4-kb fragment (3087-Bp, Fig. 3-II-A). However, the sequence containing modules B and C (B:C-Bp) had no enhancer function in the forebrain or eyes up to 24 hpf (Fig. 2). The *six3a *promoter analysis by Wargelius et al. indicates that the retinal enhancer at 48 hpf is located in the 1.2-kb fragment between -3302 and -1409, which contains both Brn3 and Pax6.1 binding sites. Our B:C module (-2932 to ~ -1692) overlapped with this 1.2-kb fragment. It is possible that module B:C-Bp functions late only in the retina.

The 1060△42-Bp and 1060-Bp constructs (Fig. 3-II-C) displayed the same activity, indicating that the 42 bp in module D can be excised without change of function (the sequence is shown in Fig. 5 black box). Although 1060△42-Bp exhibits very strong activity, 898-bp shows much lower expression both early and late (Fig. 2), indicating that the sequence differences between these constructs (-1755 to -1594) contributed dramatically to the transcription of *six3a*. This sequence contains two DNA fragments that were shown to have no enhancement function in other constructs, part of the B:C module (-1755 to -1693) and the 42 bp in module D (-1646 to -1605). Thus, the most important elements in module D were minimized to the 46-bp region (-1692 to -1647). From electrophoresis mobility shift assays (EMSAs), we also found that the 46-bp region interacted with proteins present in the 24 hpf nuclear extracts (data shown in the EMSA section).

From our serial deletion mutants, the sequence between 681-Bp (Fig. 3-II-F) and 448-Bp (Fig. 3-II-G) seemed to contribute, to some degree, to GFP expression at 24 hpf. Half of the embryos bearing the 681-Bp construct expressed GFP at 24 hpf in the forebrain, but 448-Bp is very similar to Bp, and only 4% of embryos expressed GFP. This region actually contains the Box E discovered by Bovolenta's lab, which is a late neural and retinal enhancer in medaka *six3.2 *[32]. Consistent with their discovery, our results suggested that a booster enhancer is located between 681-Bp and 448-Bp.

### Identification of modules D and A, together with the basal promoter, as early and late enhancers

From our cross-species comparison, we identified ten conserved non-coding modules. We then tested the function of individual modules by transgenesis. First, we tried to identify the basal promoter. From our transgenesis results, we found that the sequence from -694 to +110 drove weak GFP expression restricted to 1-3 cells in the forebrain, which was only observed at 24 hpf. The expression rate of the Bp was about 10% (Fig. 3-III-A), suggesting that this region only has weak promoter activity. Thus, we named it "basal promoter", which contains the transcriptional start site (Fig. 3-I, black arrow). Wargelius et al. found that the zebrafish *six3a *sequence between 805 and 236 bp upstream of the ATG is required for early expression. Our results showed that a similar sequence located in the basal promoter (695 bp from the first exon) has a basal level of transcription activation up to 24 hpf, consistent with Wargelius's discovery. Conte et al. found that the sequence upstream of the translation start site in medaka *six3.2 *(Boxes I and L) is responsible for brain expression at later stages (stages 24 to 40) [32]. Because the stages of our study were early compared to Wargelius's [33], we cannot conclude that the basal promoter does not have an enhancer function at later stages as discovered in medaka.

Embryos injected with modules 1, 3, B, C, 4, 5 or 6 individually showed expression patterns similar to that of the basal promoter. Only 5-22% of embryos carrying these constructs expressed low levels of GFP, as shown in Fig. 3-III-B for module 1. In spite of the sequence conservation, modules 1, 3, 4, 5 and 6 did not appear to contribute to the spatio-temporal control of *six3a *in zebrafish, at least up to 24 hpf. Interestingly, neither module B nor C was functional. Previously, a possible Pax6.1 binding site in module B and a putative Brn3b binding site in module C were shown to be important for regulating *six3a *in zebrafish, but this was not found in our study. Again, these results were consistent with the deletion analysis described earlier.

Module 2 of *six3a *increased the expression of GFP in embryos to 53% compared to 5% with Bp alone (Fig. 2-III-C); however, the results from eight batches of embryos were inconsistent (Additional file 5). Module A and D enhanced Bp expression dramatically, and the results from eight batches of experiments were consistent (Additional file 5). Fig. 3-III-B shows the strongest expression pattern of module 2. Half of the time, module 2 resulted in no expression. On the other hand, the expression pattern from modules A and D were always "strongly expressed in a large number of cells", but the expression from modules 1, 3, B, C, 4, 5 and 6 were always "expressed in only a few cells". Embryos injected with module A showed GFP expression in the forebrain and eyes from 14 hpf (data not shown). At 24 hpf, GFP was strongly expressed in the forebrain and retina (Fig. 3-III-D).

Because zebrafish *six3a *is expressed from 6 hpf and constitutively expressed in the neuroectoderm throughout anterior neuroectoderm formation [14], Bp, module 2 and A were not sufficient to control *six3a *expression. This suggests that an extra module is responsible for zebrafish *six3a *expression during early stages. Bovolenta and coworkers showed that a 4.5-kb region upstream of the medaka *olsix3.2 *transcription initiation site is responsible for *olsix3.2 *expression. They identified an early neural enhancer, Box D. We aligned the medaka and zebrafish genomes and found that Box D of Conte et al. was similar to the *six3a *genomic sequence -1754 to -1571 bp in zebrafish; we named it module D. Module D significantly activated transcription (Fig. 3-III-E, F), and it was the only module that drove expression in early stages (Fig. 3-III-G for 8 hpf, 3III-H for 11 hpf). Our result showed that the early neural enhancer in medaka *olsix3.2 *also appears in a similar position and exhibits an enhancer function for *six3a *expression in the early zebrafish neuroectoderm. At 24 hpf, modules D and A displayed an enhancer effect when linked to the basal promoter, except the expression domain from D-Bp (Fig. 3-III- F, I), which seemed to extend to a more ventral region than A-Bp (Fig. 3-III-D, L). This suggests that a suppressor function is missing from D-Bp. More related data is shown in the "silencer function of module A on the suppression of the ectopic expression from module D" section.

### Deletion analysis of module A identified several activator binding sites

To understand the transcriptional regulation of *six3a *in detail, we dissected module A by using deletions and mutations to identify the minimal sequence and the bound transcription factors required for enhancer function. Comparison of module A sequences among zebrafish, *Xenopus*, opossum, *Tetraodon*, human and mouse revealed four highly conserved elements with 100% similarity among those species (Fig. 4, bottom). We first generated five deletion constructs by PCR (Fig. 4-I). The A462-Bp construct was 462 bp long and lacked 311 bp from the 5'-end of module A. In the A433-Bp construct, 29 bp were deleted, including the first highly conserved element "TCATTAA". In the A415-Bp construct, the second highly conserved element "CGCTAACAA" was deleted. Both of the "AAATGC" and "CAGCT" elements were deleted in the D392-Bp construct. A 22-bp, highly conserved element was deleted in the A315-Bp construct. These constructs were separately microinjected into zebrafish embryos at the one-cell stage, and the zebrafish were analyzed for GFP expression. A462-Bp mutants showed GFP expression in the forebrain and retina at 24 hpf (Fig. 4-III-A). Furthermore, deletion of the first element (A433-Bp) slightly decreased GFP expression in the forebrain (Fig. 4-III-B). The deletion of the second highly conserved element (A415-Bp) caused decreased GFP expression in the eye and forebrain (Fig. 4-III-C). A construct containing none of the highly conserved elements (A392) showed very low expression in both the forebrain and eye, suggesting the importance of those elements (Fig. 4-III-D).

We further deleted the 3'-end of module A to find the minimal active elements. The 3' deletion mutant A△ 372-Bp, which contained all four highly conserved elements, had very low GFP expression and was expressed in only 30% of embryos. It was similar to the 5' deletion mutant A392-Bp. Although A392-Bp and A△372-Bp each contained half of module A, neither could drive GFP expression, indicating the possibility of cooperation of the transcriptional factors bound to each of these two DNA fragments. We therefore examined the functions of the four highly conserved elements plus the downstream sequences up to A△298 (Fig. 4).

The importance of the four highly conserved elements was demonstrated by the serial deletion mutants, in which we successively removed more sequence from the A△298-Bp construct. Comparison of the results between A462△298-Bp and A433△298-Bp (Fig. 4-III-E, F) confirmed the importance of the enhancer function in the "TCATTAA" sequence. Analysis of the results between A462△298-Bp and A462△298-Bp-M (Fig. 4-III-F, G) corroborated the importance of "CGCTAACAA" for forebrain and eye development. The GFP expression pattern between A462△298-Bp-M and A392△298-Bp (Fig. 4-III-G, H) supported the activation function of two highly conserved elements "AAATGC" and "CAGCT". Duplication of the 22-bp, highly conserved element showed no activation function at all. This again indicated that multiple elements must work together, and implied that transcription factor occupancy of these *cis*-elements has a synergistic effect.

### Deletion analysis in module D elucidated multiple activator binding elements

As mentioned previously, module D is the only module that drives expression in early stages of zebrafish development. Therefore, we next dissected module D to find the minimal regulatory elements. Alignment of this module among *Tetraodon*, fugu and medaka revealed clusters of conserved regions (Fig. 5-I). The comparison of the results between D141-Bp and D105-Bp showed decreased GFP expression in the eyes and forebrain in D105-Bp-injected embryos (Fig. 5-III-B, C). Further removal of nucleotides between D105-Bp and D69-Bp decreased GFP expression at both 11 and 24 hpf (Fig. 5-III-C, D). The deletion to a shorter construct, D30-Bp, which contained a highly conserved 28-bp region, showed GFP expression in the eyes and forebrain (Fig. 5-III-E). When we duplicated this element (D28(_2_)-Bp), it drove GFP not only at 24 hpf (Fig. 5-III-F) but also at 8 and 11 hpf. Our results indicated that the highly conserved 28-bp sequence might be bound by some early transcription factor(s). Mutation of six nucleotides in this 28-bp element from "ctaatt" to "AGCCGG" abolished the enhancer activity, indicating that the occupancy of transcription factor(s) at this core sequence was responsible for activation. We then used the ctaatt sequence to search the transcription factor binding site database PROMO http://alggen.lsi.upc.es/cgi-bin/promo_v3/promo/promoinit.cgi?dirDB=TF_8.3 and found that POU1F1a binds to "CTAAT" and "ATTAC" in the 28-bp highly conserved element. Both sequences were destroyed in our D28M(_2_)-Bp construct.

In contrast to module A, the multiple elements in module D seem to function independently. Constructs D184△74-Bp (Fig. 5-III-G) and D69 (Fig. 5-III-E) represent the 5' and 3' halves of module D; both drove expression in the neuroectoderm, although at a lower level compared to the full-length construct. However, comparing the three deletion mutants D184△30-Bp (Fig. 5-III-H), D105△30-Bp (Fig. 5-III-K) and D69△30-Bp (Fig. 5-III-O), we found that in the absence of the 28-bp region (removed in △30), the sequence between D184 and D105 was essential for expression. Previous deletion mutants indicated that the most important elements in module D included the 46-bp region between -1692 and ~ -1647, which co-localizes exactly with the sequence between D184 and D105.

### Silencer function of module A for the suppression of the ectopic expression of module D

**Figure 6:**
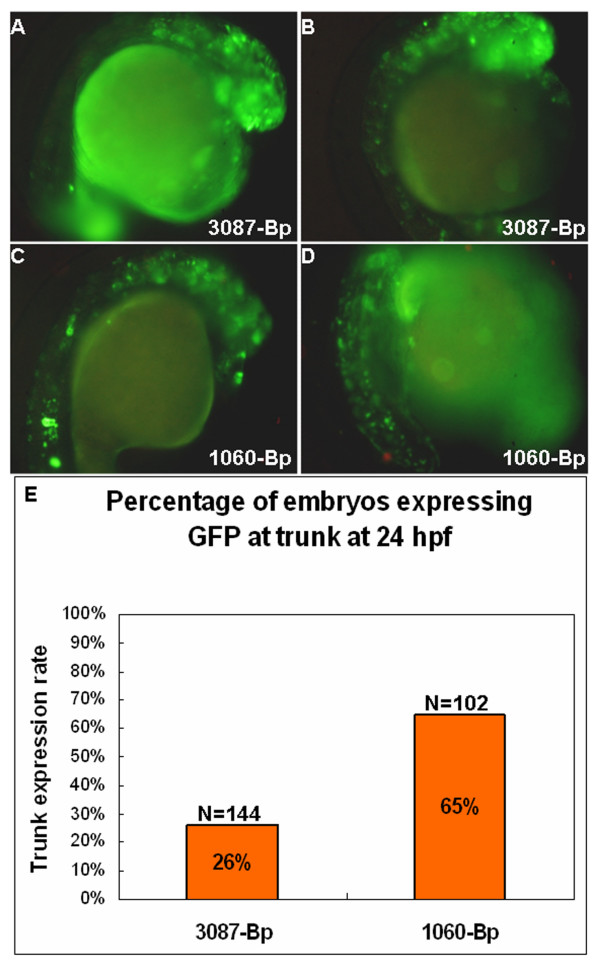
**Module A suppresses ectopic expression of module D**. Images of 24 hpf embryos showing the forebrain and eye expression pattern from 3086-Bp (A, B) and ectopic expression from 1060-Bp to the notochord (C) or the whole trunk (D). (E) Statistical analysis for 144 embryos injected with 3087-Bp and 102 embryos injected with 1060-Bp. Sixty-five percent of embryos carried 1060-Bp expressed ectopically to the trunk, while only 26% of embryos carried 3087-Bp expressed ectopically, with few cells per embryos.

We previously discovered that the *six3a *module D exhibited a strong enhancer function, although ectopically to the more ventral region. Similarly, the construct D184 42-Bp expressed GFP not only at an early stage (Fig. 5-III-N) but also strongly at a later stage (Fig. 5-III-I) and ectopically in the notochord. Conte et al [32] also found ectopic expression of Box D (*six3a *module D). Further, they found that Box A (*six3a *module A) has a silencer effect that eliminates ectopic expression. We performed statistical analyses on the percentage of trunk expression for two constructs: 3087-Bp (containing modules A and D) and 1060-Bp (containing only module D). We found 65% ectopic expression in 102 embryos injected with 1060-Bp but only 26% ectopic expression and fewer GFP-expressing cells per embryo that were injected with 3087-Bp (N = 144) (Fig. 6). Our results supported the idea that module A contains a silencer function. Additional images that depict the silencer function of module A plus module D (3087-Bp) verses module D alone (1060-Bp) are provided in Additional File 6.

### EMSA for modules D and A

**Figure 7:**
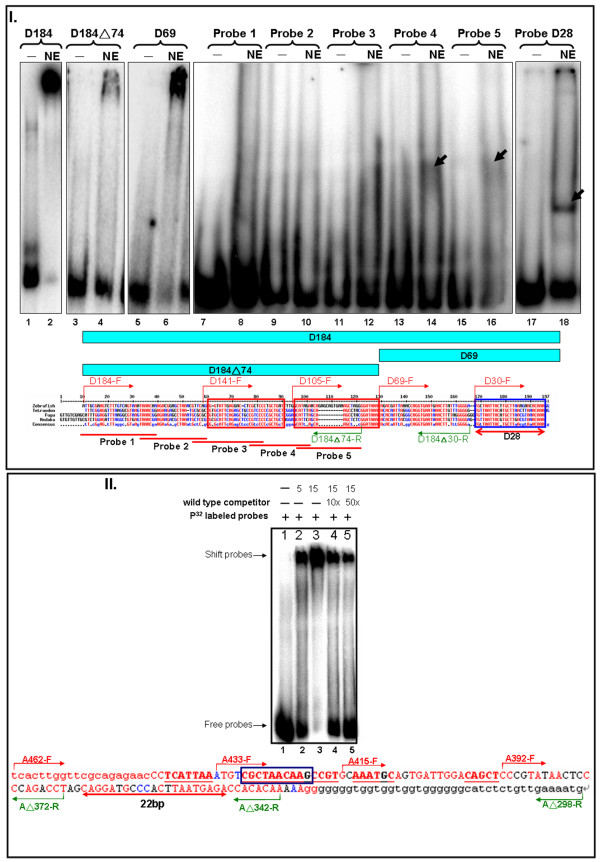
**EMSA analysis of modules D and A**. **I**. EMSA of module D using nine different probes. D184 and two of the smaller probes: D184(triangle)74 covers the 5' half, and D69 covers the 3' half were used for EMS. Five different double-stranded oligonucleotides were generated and labeled as probes for EMSA. The 28-bp region (blue box) was generated as double strand oligonucleotide for EMSA. The gel image was obtained with probe only (lane 1, 3, 5, 7, 9, 11, 13, 15 and 17), and with nuclear extract (lane 2, 4, 6, 8, 10, 12, 14, 16 and 18) to detect the binding protein; the sequence is shown below. Conserved sequences in module D were aligned among zebrafish, *Tetraodon*, fugu and medaka, with the primers used to obtain serial deletion constructs shown in red with direction. The red boxes represent the highly conserved elements. **II**. EMSA of module A. The gel image was obtained with probe only (lane1), with nuclear extract (lane 2, 3) and with competitor (lane 4, 5) to detect the binding protein using A462(triangle)298; the sequence is shown below. The primers used to obtain serial deletion constructs are shown in green above the sequences, and their length and direction are reflected in the length and direction of the arrows. The 22-bp region (red arrow) was deleted in the A462(triangle)298-Bp-M-GFP construct. The red boxes represent the highly conserved elements.

Next, we searched for the transcription factors that bind to the important elements identified in the previous functional study. For module D, we first used the entire module sequence as a probe and found that the band was strongly retarded in the well because the DNA-protein complex was too big to migrate (Fig. 7-I, lane 1, 2). We then used the 5' and 3' halves of module D as probes and again found the complexes too big to resolve on the gel (Fig. 7-I, lane 4 ~ 6). We designed five additional probes covering the 5' half of module D (Fig. 7-I, lane 7 ~ 16). Probes #4 and #5 formed weak complexes (Fig. 7-I, lane 14, 16), but no binding protein could be detected using the other three probes. These results were consistent with the functional data showing that the region between D141 and D69 has an enhancer function. The 3' half of module D also showed strong binding activity. When we used a smaller probe containing 28 bp of the functional element in module D, there was significant binding (Fig. 7-I, lane 17, 18), indicating that the 24 hpf zebrafish nuclear extract contained transcription factors that bind to this functional element.

We found multiple elements in module A during our functional study. However, synergy between those elements is required for activation, and this may be due to cooperativity in DNA binding. We used EMSAs to test transcription factor binding to module A (Fig. 7-II) and found that a large complex was formed with the probe A462 298 in a dose-dependent manner (Fig. 7-II). The complex was reduced during competition with unlabelled DNA (Fig. 7-II). However, no band shift was detected when we used smaller probes (data not shown). It is likely that those elements bind transcription factors in the 24 h nuclear extract with low affinity. Thus, multiple elements are required to achieve a higher level of occupancy on the DNA and to activate *six3a *transcription.

**Figure 8:**
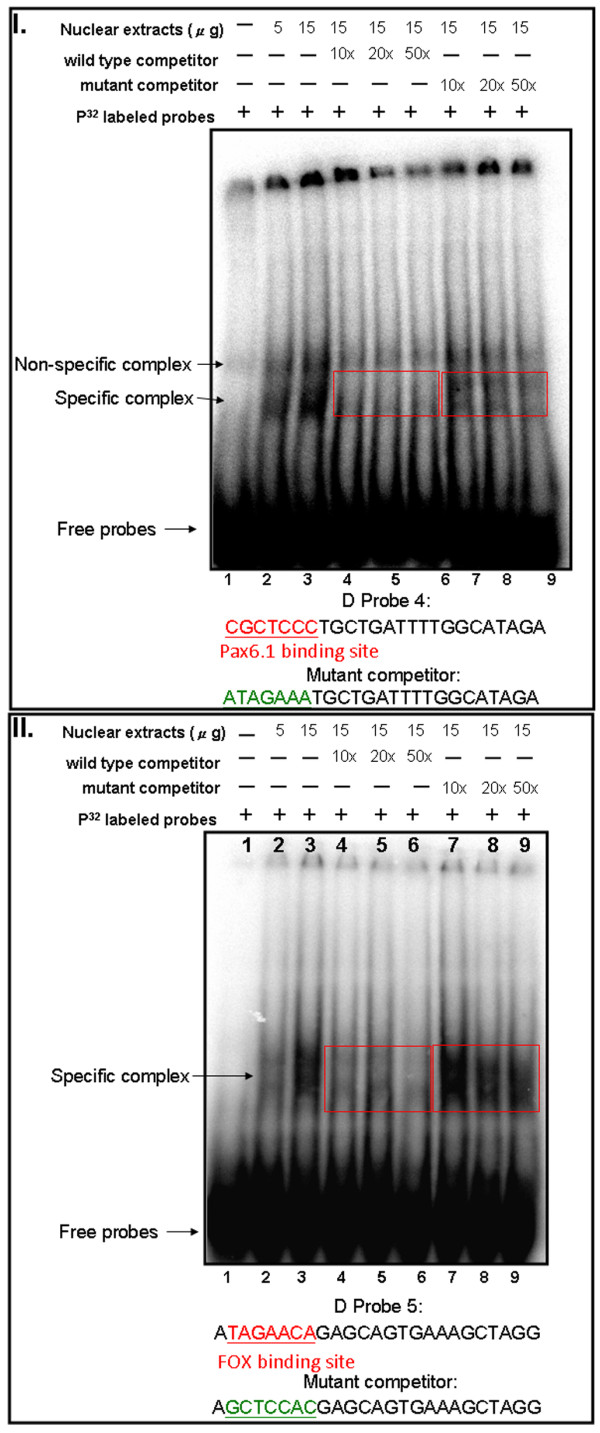
**Competition experiment for module D probes 4 and 5**. **I**. EMSA competition experiment of module D-probe 4. The gel image was obtained with probe only (lane 1) and with 5 and 15 μg nuclear extract (lane 2, 3) to detect the binding protein. The sequences (wild-type or Pax6.1 binding site mutant) for competition are shown below. Three different amounts (10×, 20× and 50×) of competitor were added to the binding reaction with 15 μg nuclear extract. Lanes 4, 5 and 6 are competition experiments with wild-type competitors at 10×, 20× and 50×, respectively, and lanes 7, 8 and 9 are competition experiments with the Pax6.1 site mutant at 10×, 20× and 50×, respectively. **II**. EMSA competition experiment of module D-probe 5. The gel image was obtained with probe only (lane 1) and with 5 and 15 μg nuclear extract (lane 2 and 3) to detect the binding protein. The sequences (wild-type or FOX binding site mutant) for competition are shown below. Three different amounts (10×, 20× and 50×) of competitor were added to the binding reaction with 15 μg nuclear extract. Lanes 4, 5 and 6 are competition experiments with wild-type competitors at 10×, 20× and 50×, respectively, and lanes 7, 8 and 9 are competition experiments with the FOX site mutant at 10×, 20× and 50×, respectively.

To identify the module D-binding proteins, we searched the transcription factor binding sites and recognized one Pax6.1 binding site in the #4 probe (Fig. 8-I), and one FOX binding site in the #5 probe (Fig. 8-II). To investigate if those sites were responsible for the binding, competition experiments with either wild-type or mutant unlabelled DNA were performed. As shown in Fig. 8-I, the specific complex on the module D-probe #4 was in competition with wild-type but not with Pax6.1 mutant DNA. In addition, the binding activity of the module D-probe #5 was in competition with wild-type but not with FOX site mutant DNA (Fig. 8-II). These data indicated that sites for Pax6.1 and FOX were indeed responsible for the binding activity.

**Figure 9:**
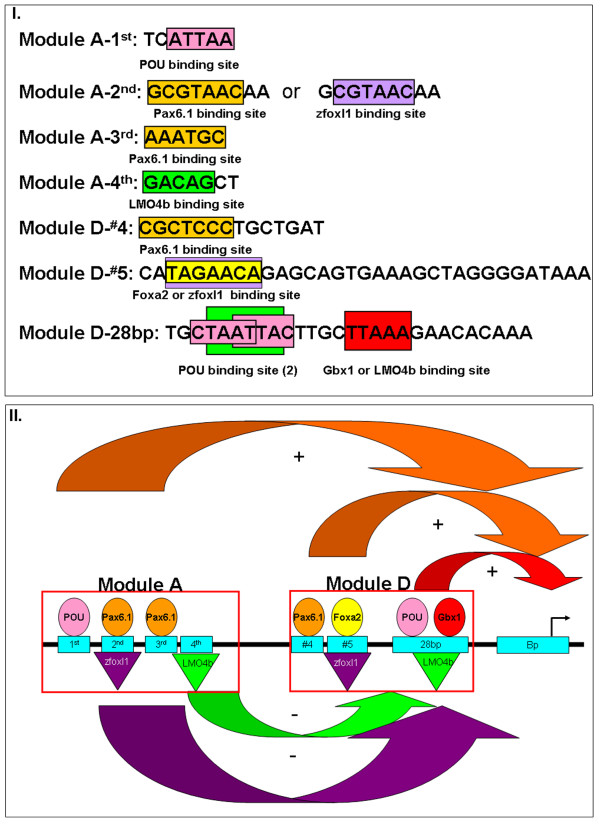
**Cartoon figure of zebrafish *six3a *regulation by modules A and D**. The black line represents the *six3a *genomic DNA, and the blue boxes represent the functional and evolutionarily conserved elements identified in this study. There are four elements in module A and three in module D. Each module is occupied by predictive transcription factors, some of which are activators (circles), and others are repressors (triangles). The 28-bp element in module D is bound by the POU and Gbx1 transcription factors, enhancing expression by interacting with the basal promoter. Elements #4 and #5 in module D, presumptively occupied by Pax6.1 and Foxa2, also enhance *six3a *expression by interacting with the basal promoter. There are four elements in module A, none of which can function independently. Through the interaction between POU and Pax6.1 and the 1^st ^to 3^rd ^elements, module A enhances *six3a *expression by interacting with the basal promoter. To inhibit the ectopic expression of *six3a*, zfoxl1 binds to module A through the 2^nd ^element and module D through the #5 element, possibly eliminating the Foxa2 interaction with the same DNA. Alternatively, LMO4b may bind to the 4^th ^element in module A and simultaneously to the 28-bp element in module D, perhaps eliminating Gbx1 binding to the same DNA. The repression effect can be achieved by module A alone or with both modules together.

Further experiments are necessary to demonstrate the identity of other transcription factors that regulate *six3a*. From predictions for module A, we discovered the Pax6.1 binding sites on the 2^nd ^and 3^rd ^conserved elements. The POU domain transcription factor binding site was predicted in the 1^st ^conserved element of module D (Fig. 9-I). We also found homeobox protein binding sites on both module D (#5 probe) and module A (4^th ^conserved element). LMO4b is a homeobox protein that is a repressor for *six3a *[25]. Further experiments evaluating the binding of LMO4b to module D and A will be necessary to provide direct evidence for this interaction. However, from our functional assay and EMSA analysis, we strongly proposed the importance of multiple elements in modules D and A, and demonstrated the binding proteins present in zebrafish embryo nuclear extracts. The EMSAs also revealed synergism between the multiple elements in modules D and A. Our results suggested that the functional response of the *six3a *transcription unit to positive inputs from POU, Pax6.1 and Gbx1 and to negative inputs from zFoxl1 and LMO4b is encoded in the *six3a cis*-regulatory elements, i.e., modules A and D. This study provides a comprehensive view of the regulation of brain and eye and may establish the GRNs underlying neurogenesis in the zebrafish.

## Discussion

### Two evolutionarily conserved modules and many minimal functional elements work together to achieve correct *six3a *expression

Six3a is an important transcription factor expressed in the presumptive brain and eye territory and continuously expressed in the forebrain and retina. Forebrain development is evolutionarily conserved among vertebrates and so is the underlying mechanism that operates the GRNs. By analyzing the transcriptional regulation of *six3a*, we not only uncovered the evolutionary *cis*-regulatory code responsible for the expression of *six3a*, but also drew a preliminary picture of how those transcription factors specify forebrain development.

We found that the early enhancer module D activated six3a expression as early as 8 hpf. However, expression extended to the ventral area. Module A was necessary to restrict this expression. Bovolenta's lab found that Box D (similar to our module D) is an early enhancer, and Box A (similar to our module A) is a silencer [32]. The interactions between these two Boxes are necessary for correct *six3.2 *expression in medaka. They also found that Box E is a late neural and retinal enhancer of medaka *six3.2*. Our results are consistent with their findings and suggest that a booster enhancer is located between 681-Bp and 448-Bp (similar to Box E). However, it is a very weak enhancer compared to modules A and D.

There are also some inconsistencies between our data and previous reports on *six3a*. We found that module A acted as an enhancer at 24 hpf, and the basal promoter had no enhancer function, as suggested by the Bovolenta [32] and Fjose [33] groups. Because the developmental stages of our study are early compared to the aforementioned studies, we cannot conclude that the basal promoter does not have an enhancer function at later stages, as they discovered. However, we have injected the Bp-GFP construct and observed expression at later stages (48 and 72 hpf), but we still do not detect GFP expression (data not shown). This preliminary result indicated that this region (the basal promoter of *zsix3a *and Box I and L of *olsix3.2*) does not have enhancer activity. Also, module B:C had no enhancer function, as proposed by Wargelius et al. However, we suggest that POU and Pax6.1 activate *six3a *expression by binding to modules A and D.

We not only demonstrated the functionality of these evolutionarily conserved elements using transgenesis in zebrafish, but we also made many deletion and mutation constructs to identify the minimal elements necessary for regulation in order to predict the transcription factors responsible for *six3a *regulation. EMSAs, using nuclear extracts from zebrafish embryos, revealed binding proteins that occupied these functional elements. The predictions of transcription factors are given in Fig. 9-I, and the working model for *six3a *regulation is shown in Fig. 9-II.

### The POU domain transcription factor is important for activating *six3a *expression

We searched for transcription factor binding sites on the evolutionarily conserved and functional minimal elements that we identified. The 28-bp, conserved element has two HNF1Bs (GCTAATTA and TAATTACT), two POU domain transcription factor (CTAAT and ATTAC), one C/EBP beta (TTGCTTA) and one PR-alpha (AGAACACAA) binding sites. Mutational analysis showed that the POU domain transcription factor binding site was responsible for the enhancer function; mutation of the six-nucleotide sequence containing this site eliminated the enhancer function. In module A, four highly conserved elements work synergistically as an enhancer. The first element (TCATTAA) has a binding site for the POU domain transcription factor (ATTAA).

Two POU domain transcription factors are expressed in zebrafish brain and eyes: pou3f3a (*POU class 3 homeobox 3a; *previous names:*zp12, wu:fc33a11, pou12 and brn1.1*), which is predominantly expressed in the central nervous system [35]; and *pou3f3b (POU class 3 homeobox 3b*; previous names:*zp23pou, wu:fb92g06(1), zp23, fb92g06, zfpou1(1), pou1 and pou23*), which is expressed in developing neural tissues [36]. They are expressed at the right time and place for the activator to bind to *six3a *modules A and D. It is likely that Pou3f3a and Pou3f3b are important in regulating *six3a *expression by binding to modules A and D. Although we did not find that the proposed Brn3 (which is a POU domain transcription factor) binding site on module B had any activation function, as described previously [33], we suggest that Pou3f3a and Pou3f3b activate *six3a *by binding to the 28-bp element in module D and the first highly conserved element in module A.

### Pax6a binds to *six3a *module A to activate expression in the eyes and brain

In module A, the second element (GCGTAACAA) has a binding site for a paired box protein (GCGTAAC), and the third (AAATGC) also has a binding site for a paired box protein (AAATGC). In module D, in addition to the 28-bp element, two highly conserved elements show enhancer function and are bound by proteins in the 24 h nuclear extract. The EMSA probe #4 (**CGCTCCC**TGCTGATT) has a binding site for a paired box protein (CGCTCCC).

Many paired box proteins in zebrafish are likely important in regulating *six3a *expression. Among these, *pax6a (paired box gene 6a)*, which is expressed in the anterior neural plate at the bud stage [37] and in the brain and immature eye at the prim5 stage, is the most likely activator of module D expression. The regulatory mechanisms governed by *pax6 *are evolutionarily conserved among teleosts and mammals [38]. Although we did not find that the proposed Pax6 binding site on module C had any activation function, as proposed previously [33], we suggest that Pax6a activates *six3a *by binding to the 2^nd ^and 3^rd ^highly conserved elements in module A.

### Other possible transcription factors regulating *six3a *expression

The 28-bp conserved element of module D has two homeobox protein binding sites (TAATTA), and the fourth element of module A (GACAGCT) has a binding site for HoxA3 (GACAG). It is possible that a homeobox protein, Gbx1 (gastrulation brain homeobox 1), activates *six3a *expression as early as 5 hpf. Our unpublished real-time RT-PCR data indicated that knockdown of *Gbx1 *expression by morpholino injection decreased *six3a *expression at 5 hpf. Gbx1 is expressed in the presumptive brain at the 75% epiboly stage [39]. However, if Gbx1 expression extends to the hindbrain neural plate, neural plate and ventral mesoderm [40], early expression will result in an ectopic pattern unless there is a repression system. Another homeobox transcription factor candidate for regulating *six3 *expression is the LIM homeobox gene, *LMO4b*, which has been proposed as a negative regulator of forebrain growth acting via the restriction of *six3 *expression during early segmentation stages [25]. It is possible that LMO4b negatively regulates *six3a *by binding to modules A and D together.

We found that the second conserved element (CGTAACA) of module A shows similarity to the forkhead transcription factor binding site. The fifth EMSA probe of module D (CA**TAGAACA**GAGCAGTGAAAGCTAG) has a binding site for the forkhead transcription factor (TAGAACA). Thus, the forkhead transcription factor may also activate module D expression in the presumptive brain. Foxa2, also called hepatocyte nuclear factor 3β (hnf-3β), is a member of the forkhead box transcription factor protein family. *In situ *hybridization shows that Foxa2 is first expressed on the dorsal side of the hypoblast just before gastrulation [41]. Later, at 8 hpf, it is expressed in the endoderm and axial mesoderm. In adult fish, Foxa2 is expressed in multiple territories, including the gut, liver and pancreas of the endoderm and the floor plate of the ectoderm [42]. Thus, during zebrafish development, Foxa2 is expressed in all three germ layers. Its role in the ventral central nervous system was proposed by Norton et al. Nodal signaling from the notochord induces Foxa2 expression in the medial floor plate (MFP), and Foxa2 activates downstream genes (e.g., Ntn1b, Shha and Shhb) that are required for MFP maintenance and differentiation.

One of the forkhead transcription factors, zfoxl1, is strongly expressed in neural tissues, such as the midbrain, hindbrain and the otic vesicle at the early embryonic stage. It is a novel regulator of neural development that acts by suppressing *shh *expression [43]. Our results and previous data from Conte et al. [32] indicated that the suppressor function mediated by module A eliminates the ectopic expression carried by module D. It is possible that zfoxl1 binds to module A via the 2^nd ^conserved element and to module G through the 28-bp element to execute its repressor function over module D.

## Conclusions

In this study, we identified two enhancers in the regulation of zebrafish *six3a*. Those modules are evolutionarily conserved across all vertebrates. Module D was also identified as an enhancer in medaka. Module A not only exhibited an enhancer function but also had a suppressor effect, eliminating the ectopic expression driven by module D. Further analysis of the minimal binding elements in both modules demonstrated that the multiple elements in module A work synergistically in binding to DNA, while the 45-bp and 28-bp elements in module D have strong DNA-protein interaction activity and are functionally important. Possible activators that bind to modules D and A are Pou3f3a, Pou3f3b, Pax6a, Gbx1 and Foxa2. Possible repressors binding to module D and A are LMO4 and zfoxl1. The discovery of these elements and transcription factor binding sites provides a new insightful view of the entire interplay of transcription factors in the GRNs for forebrain development.

## Methods

### Zebrafish husbandry, experimentation and care/welfare

AB strain *D. rerio *fish were purchased from the Zebrafish International Resource Center (ZIRC), Oregon. Fish were maintained at 28°C in our zebrafish facility, a continuous flow-through system, with a 14 h light/10 h dark cycle. The developmental stages were as described previously [44]. Embryos from naturally spawning AB strain zebrafish were used in this study. To generate embryos for injection, male and female fish were placed in a 1 L fish tank with inner mesh and a divider the night before injection. Zebrafish embryos were obtained from naturally spawning adults stimulated by light and by removing the divider. The embryos were stored at 28.5 before and after microinjection.

### Ethical approval

All experiments involving zebrafish were conducted according to the guidelines of Institutional Animal Care and Use Committee (IACUC) of the National Health Research Institutes (NHRI). The animal protocol involving zebrafish was approved by IACUC of NHRI; the approved protocol numbers are NHRI-IACUC-095050-A, 096037-A, 098017-A and 098087 under the name of Dr. Yuh, Chiou-Hwa, who is the corresponding author.

### DNA constructs and site-directed mutagenesis

The zebrafish *six3a *DNA was obtained from zebrafish genomic DNA or a *six3a *BAC clone (DKEY-254J21) purchased from the BACPAC Resource Center (BPRC) at the Children's Hospital, Oakland Research Institute, Oakland, CA. The genomic sequence was obtained from the Ensembl Genome Browser Database http://www.ensembl.org/Danio_rerio/index.html. *Cis*-regulatory elements in the zebrafish *six3a *genome, which are conserved among several species, were identified using the UCSC Genome Browser Database http://genome.ucsc.edu/cgi-bin/hgGateway. In order to obtain the *cis*-regulatory elements for our constructs, we used PCR to amplify the BAC DNA by specific forward and reverse primers containing restriction enzyme sites for ligation into the pEGFP-N1 vector (BD Biosciences, San Jose, CA). After digesting with restriction enzymes and purifying from the gel, the DNA was inserted into an EGFP-N1 vector. The primer sequences used to amplify the DNA are shown in [additional data 1], and the restriction enzyme sites are underlined.

Site-directed mutagenesis was performed using a QuickChange Site-Directed Mutagenesis kit (Stratagene). This method utilizes *PfuTurbo *DNA polymerase, which replicates both plasmid strands with high fidelity and without displacing the mutant oligonucleotide primers. A 50-ng sample of template DNA was used in each reaction. After temperature cycling (95°C for 30 s, then 18 cycles of 95°C for 30 s, 55°C for 1 min and 68°C for 12 min), the product was treated with *Dpn*I. The nicked vector DNA containing the desired mutations was then transformed into XL2-Blue ultracompetent cells. The oligonucleotides used to generate the mutations are given in Additional file 3. The underlined sequences are the 5' and 3' parts of the primer sequences. The internal sequences were deleted after site-directed mutagenesis.

### Microinjection and microscopic photography

Embryos were injected using either DNA from the construct, which was PCR-amplified with the specific forward and reverse primers (EGFP-N1-r-poly(A) containing the SV40 poly(A) signal), or the linearlized constructs formed by digestion with *Xho*I. For microinjection, the morpholinos or DNAs were prepared in PBS with 0.05% (w/v) phenol red. Embryos were injected at the one-cell stage with 2.3 nl of 25 ng/μl *six3a*-green fluorescent protein (GFP) constructs by Nanoject (Drummond Scientific Co., Broomall, PA). Embryos were collected at different stages for GFP visualization and photography using a Nikon Optiphot-2 upright microscope with the episcopic-fluorescence attachment EFD-3 (Nikon Inc., Melville, NY) coupled with a DXM1200 Nikon Digital Camera.

### EMSA

Radioactively labeled DNA probes were designed based on the functional assay results. The G184 EMSA probe was generated by PCR using the D184-F and D184-R primers and was digested with *Xho*I to produce 5' protruding ends for labeling. The D184△74 probe was generated by PCR using the D184-F and D184△74-R and was digested with *Xho*I to produce 5' protruding ends for labeling. The D69 probe was generated by PCR using the D69-F and D184-R primers and was digested with *Xho*I and *Sac*I to produce 5' protruding ends for labeling. The #1-#5 and D28 probes in module D were synthesized oligonucleotides and annealed to form double strands using the method described below. The module A EMSA probe was generated by PCR using the A462-F and AΔ298-R primers and was digested with *Xho*I and *Kpn*I to produce 5' protruding ends for labeling.

The oligonucleotides used for EMSA and PCR primers for generating the DNA fragments for EMSA are listed in Additional file 1. The 5' protruding sequence on the annealed double-stranded oligonucleotide probes, 5'ATCG, that generated by restriction enzyme digestion, was used for Klenow labeling by filling in with [α^32^P] dCTP. The double-stranded DNA was annealed in 0.1 M NaCl buffered with 10 mM Tris (pH 8.0) at a 100 μM final concentration. The double-stranded DNA was labeled with Klenow polymerase, and the free un-incorporated nucleotides were removed with a G-25 Sephadex spin column. Reactions were prepared per tube as follows: to 2 μl nuclear extract or buffer C [45], 1 μl specific competitor (100 ng/Al) or H2O was added, plus 1 μl labeled probe (1 × 10^5^cpm/μl), 16 μl pre-mix binding buffer (containing nonspecific competitors [polyd(I)-d(C)/polyd(I)/d(C), 10 μg]), and 1× binding buffer [20 mM HEPES-KOH (pH 7.9), 75 mM KCl, 0.5 mM DTT]. The tubes were incubated on ice for 15 min before loading onto a 6% native polyacrylamide/TBE gel. The gel was run at 150 V for 2.5 h, placed on 3 MM paper, wrapped with plastic wrap, dried for 60 min at 80°C in a vacuum gel dryer and subjected to phosphoimager exposure.

## Authors' contributions

CC carried out all of the experiments in this project (including making constructs, microinjection, taking images and EMSAs) and made substantial contributions to the acquisition and interpretation of data. WH participated in the design of the study, revised the manuscript, and made critical intellectual contributions. CY was involved in drafting the manuscript, revising it critically for important intellectual content and gave final approval of the version to be published. All authors read and approved the final manuscript.

## Supplementary Material

Additional file 1 **Six3 protein sequence alignment**. Fourteen Six3 proteins from 12 different species were used for the alignment analysis. Click here for file

Additional file 2 **Phylogenetic tree of 14 Six3 proteins from 12 different species**. The tree was built using CLC Main Workbench 5 software with the Neighbor Joining method. The neighbor joining algorithm is generally considered to be fairly good and is widely used. The number indicates the bootstrap score, which shows that the corresponding branch occurs in all 100 trees made from re-sampled alignments. Thus, a high bootstrap score is a sign of greater reliability. Click here for file

Additional file 3 **Primers used in this study**. All of the primers used for generating PCR products for microinjection and site-direct mutagenesis are listed. Click here for file

Additional file 4 **Conserved non-coding regions or regulatory modules identified in this study**. All of the conserved non-coding regions or regulatory elements identified in this study are shown. Click here for file

Additional file 5 **Raw data for microinjection experiment**. All of the microinjection data in this study are shown. Click here for file

Additional file 6 **Additional images for 3087-Bp and 1060-Bp**. More GFP images for 3087-Bp and 1060-Bp are shown in this file. Click here for file
