# Supplementary material for: Complexity of cis-regulatory organization of six3a during forebrain and eye development in zebrafish
Source: BMC Dev Biol. 2010 Mar 26;10:35. doi: 10.1186/1471-213X-10-35 (PMC2858731; doi:10.1186/1471-213X-10-35)
Supplement: Additional file 1 — Six3 protein sequence alignment. Fourteen Six3 proteins from 12 different species were used for the alignment analysis. [file 1471-213X-10-35-S1.DOC]

## Additional file 1: Six3 protein sequence alignment:


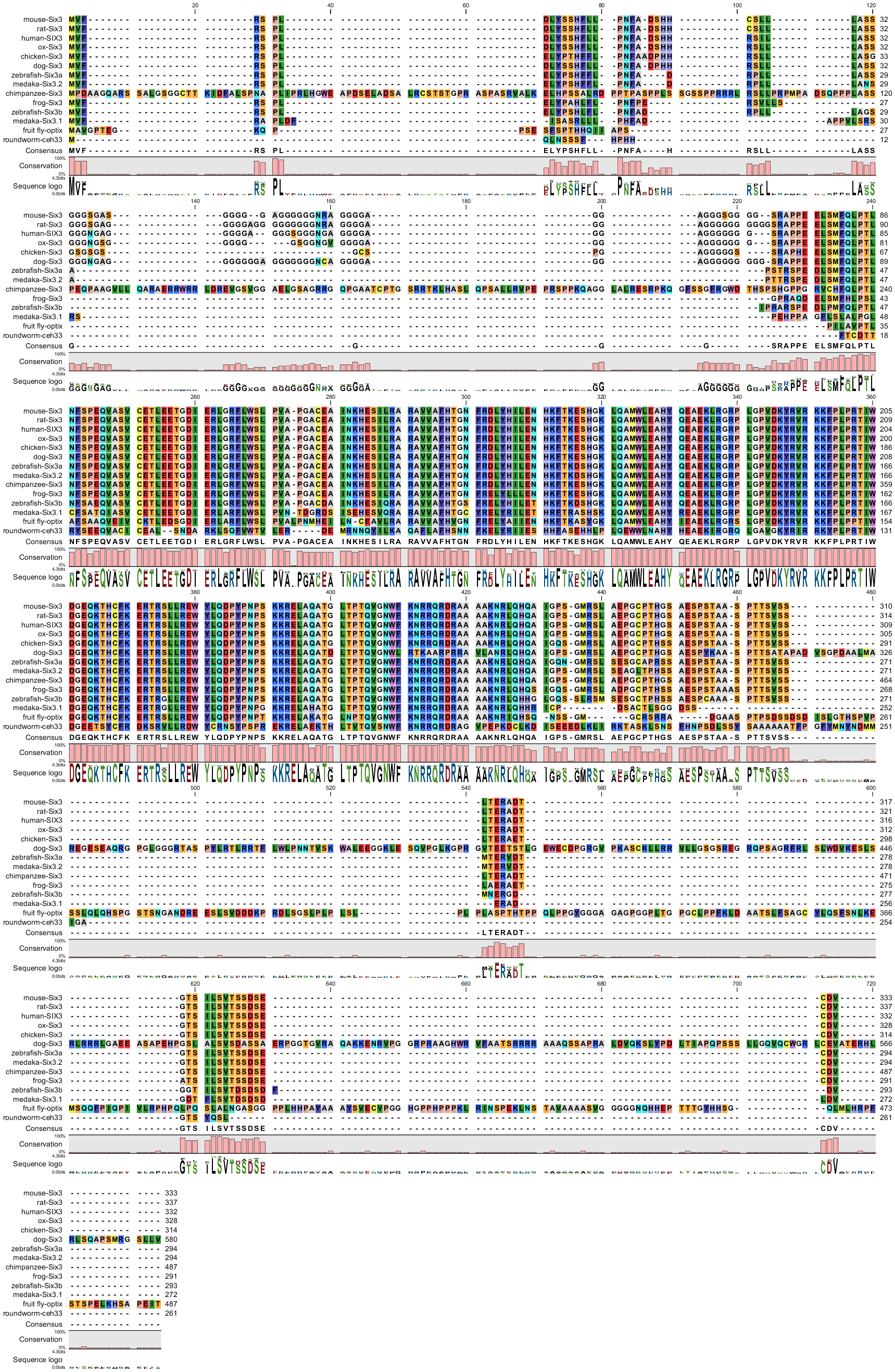


**This figure shows the alignment of *Six3* amino acid sequence from different species including human, chimpanzee, mouse, rat, dog, ox, chicken, frog, medaka, zebrafish, fruit fly, and roundworm. The protein sequences for aligment are listed below:**

1. Human: NP_005404.1| SIX homeobox 3 [Homo sapiens]

2. Chimpanzee: XP_525749.2| PREDICTED: similar to Six3b [Pan troglodytes]

3. Mouse: NP_035511.2| SIX homeobox 3 [Mus musculus]

4. Rat: NP_076480.1| SIX homeobox 3 [Rattus norvegicus]

5. Dog: XP_538477.2| PREDICTED: similar to sine oculis homeobox homolog 3 [Canis familiaris]

6. Ox: XP_873956.2| PREDICTED: similar to sine oculis homeobox homolog 3 [Bos taurus]

7. Chicken: NP_989695.1| SIX homeobox 3 [Gallus gallus]

8. Frog: NP_001079171.1| Six3 protein [Xenopus laevis]

9. Zebrafish: NP_571437.1| sine oculis homeobox homolog 3a [Danio rerio]

10. Zebrafish: NP_571438.1| sine oculis homeobox homolog 3b [Danio rerio]

11. Medaka: NP_001116400.1| putative transcription factor [Oryzias latipes]

12. Medaka: CAA04394.1| Six3 [Oryzias latipes]

13. Fruit fly: NP_524695.2| optix, isoform A [Drosophila melanogaster]

14. Roundworm: NP_504420.1| Homeobox family member (ceh-33) [Caenorhabditis elegans]

>gi|4885597|ref|NP_005404.1| SIX homeobox 3 [Homo sapiens]

MVFRSPLDLYSSHFLLPNFADSHHRSILLASSGGGNGAGGGGGAGGGSGGGNGAGGGGAGGAGGGGGGGSRAPPEELSMFQLPTLNFSPEQVASVCETLEETGDIERLGRFLWSLPVAPGACEAINKHESILRARAVVAFHTGNFRDLYHILENHKFTKESHGKLQAMWLEAHYQEAEKLRGRPLGPVDKYRVRKKFPLPRTIWDGEQKTHCFKERTRSLLREWYLQDPYPNPSKKRELAQATGLTPTQVGNWFKNRRQRDRAAAAKNRLQHQAIGPSGMRSLAEPGCPTHGSAESPSTAASPTTSVSSLTERADTGTSILSVTSSDSECDV

>gi|114577291|ref|XP_525749.2| PREDICTED: similar to Six3b [Pan troglodytes]

MPDAAGQARSSALGSGGCTTKIDFALSPNAPLIPRLHGWEAPDSELADSALRCSTSTGPRASPASRVALKELHPSSALRDPPTPASPPLSSGSSPPRRRLRSLLPRPMPADSQPPPLASSPEQPAAGVLLQARAERRWRRLDREVGSVGGAELGSAGRRGQPGAATCPTGSRRTKLHASLQPSALLRVPEPRSPPKQAGGLALRESRPKQGFSSGFRGWDTHSPSHGPPGRVCHFQLPTLNFSPEQVASVCETLEETGDIERLGRFLWSLPVAPGACEAINKHESILRARAVVAFHTGNFRDLYHILENHKFTKESHGKLQAMWLEAHYQEAEKLRGRPLGPVDKYRVRKKFPLPRTIWDGEQKTHCFKERTRSLLREWYLQDPYPNPSKKRELAQATGLTPTQVGNWFKNRRQRDRAAAAKNRLQHQAIGPSGMRSLAEPGCPTHGSAESPSTAASPTTSVSSLTERADTGTSILSVTSSDSECDV

>gi|73970118|ref|XP_538477.2| PREDICTED: similar to sine oculis homeobox homolog 3 [Canis familiaris]

MVFRSPLDLYSSHFLLPNFADPHHRSLLLASSGGGNGAGGGGGGGAGGGGGGGNCAGGGGAGGAGGGGGGGGGSRAPPEELSMFQLPTLNFSPEQVASVCETLEETGDIERLGRFLWSLPVAPGACEAINKHESILRARAVVAFHTGNFRDLYHILENHKFTKESHGKLQAMWLEAHYQEAEKLRGRPLGPVDKYRVRKKFPLPRTIWDGEQKTHCFKERTRSLLREWYLQDPYPNPSKKRELAQATDLTPTQVGNWLRTKAARPRRAVLANRLQHQAIGPSGMRSLAEPGCPTHGSAESPYKAASPTTSATAPADVSGPDAALMAREGESEAQRGPGLGGGRTASPYLRTLRRTFLWLPNNTVSKWALEEGGKLESQVPGLKGPRGVTEETSTLGEWECDPGRGVPKASCKLLRRVLLGSGSREGRQPSAGRFRLSLWDVKESLSRLRRRLGAEEASAPEHPGSLALSVSDASSAERPGGTGVRAQAKKENRVPGGRPRAAGHWRVFAATSRRRRAAAQSSAPRALDVQKSLYPDLTIAPQPSSSLLGQVQCWGRLCEVATERHLRLSQAPSMRGSLLV

>gi|119922631|ref|XP_873956.2| PREDICTED: similar to sine oculis homeobox homolog 3 [Bos taurus]

MVFRSPLDLYSSHFLLPNFADSHHRSLLLASSGGGNGSGGGGGGSGGNGVGGGGAGGAGGGGGGGSRAPPEELSMFQLPTLNFSPEQVASVCETLEETGDIERLGRFLWSLPVAPGACEAINKHESILRARAVVAFHTGNFRDLYHILENHKFTKESHGKLQAMWLEAHYQEAEKLRGRPLGPVDKYRVRKKFPLPRTIWDGEQKTHCFKERTRSLLREWYLQDPYPNPSKKRELAQATGLTPTQVGNWFKNRRQRDRAAAAKNRLQHQAIGPSGMRSLAEPGCPTHGSAESPSTAASPTTSVSSLTERADTGTSILSVTSSDSECDV

>gi|59939908|ref|NP_035511.2| SIX homeobox 3 [Mus musculus]

MVFRSPLDLYSSHFLLPNFADSHHCSLLLASSGGGSGASGGGGGAGGGGGGNRAGGGGAGGAGGGSGGGGSRAPPEELSMFQLPTLNFSPEQVASVCETLEETGDIERLGRFLWSLPVAPGACEAINKHESILRARAVVAFHTGNFRDLYHILENHKFTKESHGKLQAMWLEAHYQEAEKLRGRPLGPVDKYRVRKKFPLPRTIWDGEQKTHCFKERTRSLLREWYLQDPYPNPSKKRELAQATGLTPTQVGNWFKNRRQRDRAAAAKNRLQHQAIGPSGMRSLAEPGCPTHGSAESPSTAASPTTSVSSLTERADTGTSILSVTSSDSECDV

>gi|13027452|ref|NP_076480.1| SIX homeobox 3 [Rattus norvegicus]

MVFRSPLDLYSSHFLLPNFADSHHCSLLLASSGGGSGAGGGGGAGGGGGGGGGNRAGGGGAGGAGGGGGGGGGGSRAPPEELSMFQLPTLNFSPEQVASVCETLEETGDIERLGRFLWSLPVAPGACEAINKHESILRARAVVAFHTGNFRDLYHILENHKFTKESHGKLQAMWLEAHYQEAEKLRGRPLGPVDKYRVRKKFPLPRTIWDGEQKTHCFKERTRSLLREWYLQDPYPNPSKKRELAQATGLTPTQVGNWFKNRRQRDRAAAAKNRLQHQAIGPSGMRSLAEPGCPTHGSAESPSTAASPTTSVSSLTERADTGTSILSVTSSDSECDV

>gi|45383428|ref|NP_989695.1| SIX homeobox 3 [Gallus gallus]

MVFRSPLELYPTHFFLPNFAADPHHRSLLLASGGSGSGSGCSPGAGGGGGSSRAPHEELSMFQLPTLNFSPEQVASVCETLEETGDIERLGRFLWSLPVAPGACEAINKHESILRARAVVAFHTGNFRDLYHILENHKFTKESHGKLQAMWLEAHYQEAEKLRGRPLGPVDKYRVRKKFPLPRTIWDGEQKTHCFKERTRSLLREWYLQDPYPNPSKKRELAQATGLTPTQVGNWFKNRRQRDRAAAAKNRLQHQAIGQSGMRSLAEPGCPTHSSAESPSTAASPTTSVSSLTERAETGTSILSVTSSDSECDV

>gi|148226350|ref|NP_001079171.1| Six3 protein [Xenopus laevis]

MVFRSPLELYPAHLFLPNFPERSVLLSGPRAQDELSMFHLPSLNFSPEQVASVCETLEETGDIERLGRFLWSLPVAPGACEAINKHESILRARAVVAFHTGNFRELYLLLENHKFTKESHGKLQAMWLEAHYQEAEKLRGRPLGPVDKYRVRKKFPLPRTIWDGEQKTHCFKERTRSLLREWYLQDPYPNPSKKRELAQATGLTPTQVGNWFKNRRQRDRAAAAKNRLQHQSIGQSGMRSLADPGCPTHSSAESPSTAAASPTTSVSSLAERAETATSILSVTSSDSECDV

>gi|24586477|ref|NP_524695.2| optix, isoform A [Drosophila melanogaster]

MAVGPTEGKQPPSESFSPTHHQIIAPSPILAVPTLAFSAAQVEIVCKTLEDSGDIERLARFLWSLPVALPNMHEILNCEAVLRARAVVAYHVGNFRELYAIIENHKFTKASYGKLQAMWLEAHYIEAEKLRGRSLGPVDKYRVRKKFPLPPTIWDGEQKTHCFKERTRSLLREWYLQDPYPNPTKKRELAKATGLNPTQVGNWFKNRRQRDRAAAAKNRIQHSQNSSGMGCRSRRADGAASPTPSDSSDSDISLGTHSPVPSSLQLQHSPGSTSNGANDREESLSVDDDKPRDLSGSLPLPLSLPLPLASPTHTPPQLPPGYGGGAGAGPGGPLTGPGCLPPFKLDAATSLFSAGCYLQSFSNLKEMSQQFPIQPIVLRPHPQLPQSLALNGASGGPPLHHPAYAAAYSVECVPGGHGPPHPPPKLRINSPEKLNSTAVAAAASVGGGGGNQHHEPTTTGYHHSGQLMLHRPFSTSPELKHSAPEIT

>gi|17559042|ref|NP_504420.1| Homeobox family member (ceh-33) [Caenorhabditis elegans]

MQLNSSSFHPHHFTCDTTRYSEEQVACICEALSNDARKLSQFVWTVLERDEMRNNQYILKAQAFLAFHSNNFKELYRIIESHHFASEHHLPLQEWWLNAHYHEAEKIRGRQLGAVGKYRIRRKYPLPRTIWDGEETSYCFRDKSRVLLRDWYCRNSYPSPREKRELAEKTHLTVTQVSNWFKNRRQRDRAGVPEPKDCLKDISEEEDLKLIRKTASKLSNSFHNPSDLSSYSAAAAAATFPGFYMNYNDMMIGAGTSYQSL

>gi|18859363|ref|NP_571437.1| sine oculis homeobox homolog 3a [Danio rerio]

MVFRSPLELYPSHFFLPNFADRPLLLASSAPSTRSPEDLSMFQLPTLNFSPEQVASVCETLEETGDIERLGRFLWSLPVAPGACEAINKHESILRARAVVAFHTGNFRDLYHILENHKFTKDSHGKLQAMWLEAHYQEAEKLRGRPLGPVDKYRVRKKFPLPRTIWDGEQKTHCFKERTRSLLREWYLQDPYPNPSKKRELAQATGLTPTQVGNWFKNRRQRDRAAAAKNRLQHQAIGQNGMRSLSESGCAPRSSAESPSTAASPTTSVSSMTERVDTGTSILSVTSSDSECDV

>gi|24308522|ref|NP_571438.1| sine oculis homeobox homolog 3b [Danio rerio]

MVFRSPLELYPSHLFLPNFADRPLLLAGSIPRARSPEDLPMFQLPTLNFSAEQVASVCETLEETGDIERLGRFLWSLPVAPGACDAINKHESIQRARAVVAYHTGSFRELYHILETHKFTKDSHGKLQAMWLEAHYQEAEKLRGRPLGPVDKYRVRKKFPLPRTIWDGEQKTHCFKERTRGLLREWYLQDPYPNPSKKRELAQATGLTPTQVGNWFKNRRQRDRAAAAKNRLQHHGLGQSSLRSMSESGCTPHSSAESPCAAASPTTSVSSMNERGDGGTILSVTDSDSDFDV

>gi|171545967|ref|NP_001116400.1| putative transcription factor [Oryzias latipes]

MVFRSPLELYPSHFFLPNFADRPLLLANSAPTTRSPEDLSMFQLPTLNFSPEQVASVCETLEETGDIERLGRFLWSLPVAPGACEAINKHESILRARAVVAFHTGNFRDLYHILENHKFTKDSHGKLQAMWLEAHYQEAEKLRGRPLGPVDKYRVRKKFPLPRTIWDGEQKTHCFKERTRSLLREWYLQDPYPNPSKKRELAQATGLTPTQVGNWFKNRRQRDRAAAAKNRLQHQAIGPSGMRSLSEAGLTPHSSAESPSTAASPTTSVSSMTERVDTGTSILSVTSSDSECDV

>gi|3115307|emb|CAA04394.1| Six3 [Oryzias latipes]

MVFRAPLDFISASRLLLPHFADAPPVLSRSRSPEHPPAGFLSLALPGLCFSATQIASVCETLEETGDIERLARFLWSLPVNTDGRDSISEHESVQRARAVVAFHTGCYRELYRILETHRFTRASHSKLQAMWLEAHYREAEKLRGRPLGPVDKYRVRKKFPLPRTIWDGEQKTHCFKERTRGLLREWYLQDPYPNPGKKRELAHATGLTPTQVGNWFKNRRQRDRAAAAKNRLQHHRICPDSACTLSGGDSSERADGDTFLSVTDSDSDLDV
