# Supplementary material for: Complexity of cis-regulatory organization of six3a during forebrain and eye development in zebrafish
Source: BMC Dev Biol. 2010 Mar 26;10:35. doi: 10.1186/1471-213X-10-35 (PMC2858731; doi:10.1186/1471-213X-10-35)
Supplement: Additional file 2 — Phylogenetic tree of 14 Six3 proteins from 12 different species. The tree was built using CLC Main Workbench 5 software with the Neighbor Joining method. The neighbor joining algorithm is generally considered to be fairly good and is widely used. The number indicates the bootstrap score, which shows that the corresponding branch occurs in all 100 trees made from re-sampled alignments. Thus, a high bootstrap score is a sign of greater reliability. [file 1471-213X-10-35-S2.DOC]

## Additional file 2: phylogenetic tree of 14 Six3 proteins from 12 different species.


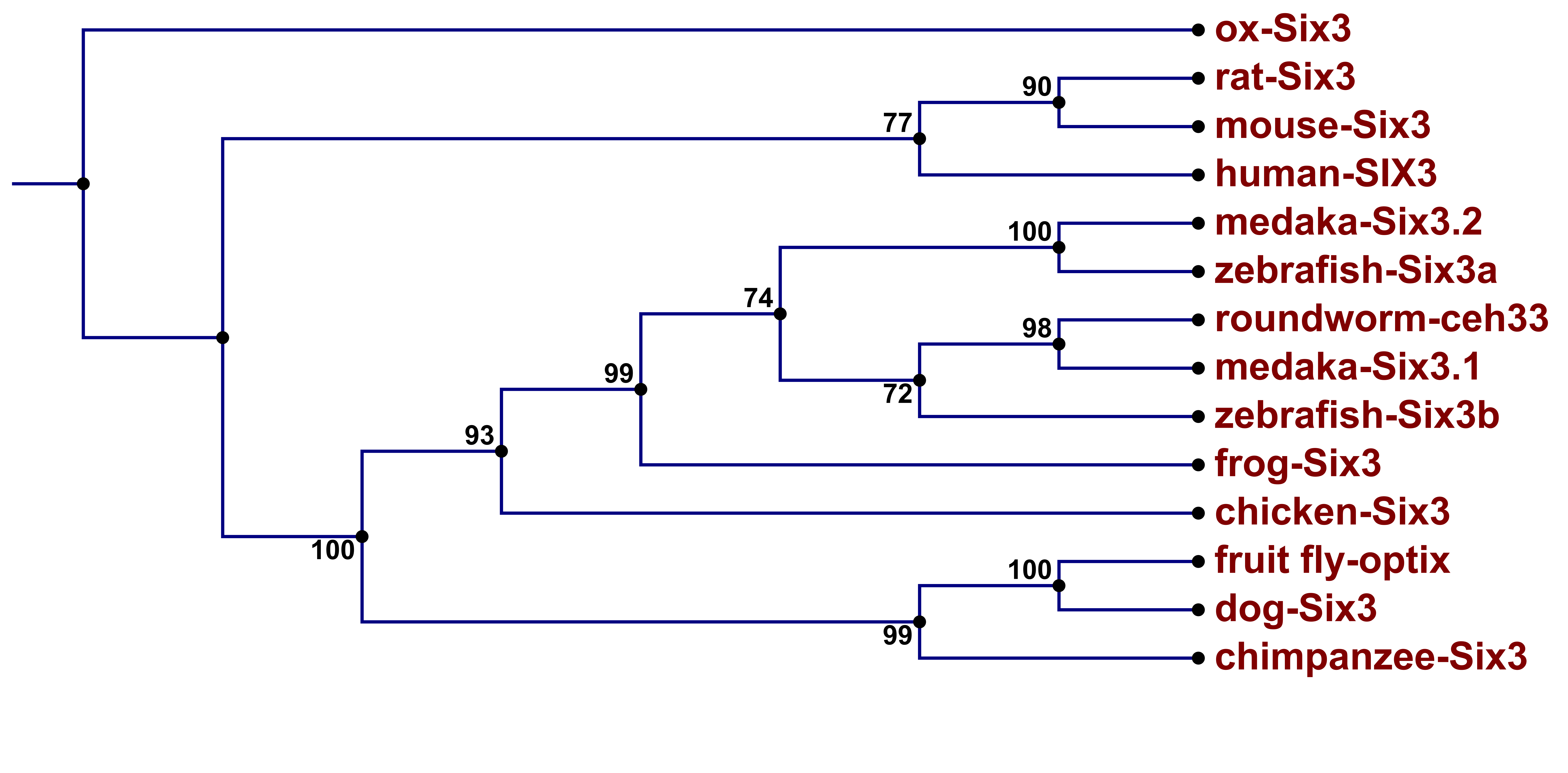


The tree was built by using CLC Main Workbench 5 software with Neighbor Joining method. The neighbor join algorithm is generally considered to be fairly good and is widely used. The number indicates the bootstrap score which shows the corresponding branch occurs in all 100 trees made from re-sampled alignments. Thus, a high bootstrap score is a sign of greater reliability.
