# Supplementary material for: Complexity of cis-regulatory organization of six3a during forebrain and eye development in zebrafish
Source: BMC Dev Biol. 2010 Mar 26;10:35. doi: 10.1186/1471-213X-10-35 (PMC2858731; doi:10.1186/1471-213X-10-35)
Supplement: Additional file 3 — Primers used in this study. All of the primers used for generating PCR products for microinjection and site-direct mutagenesis are listed. [file 1471-213X-10-35-S3.DOC]

## Additional file 3 – primers used in this study

### All the primers used for generated PCR product for microinjection, site-direct mutagenesis are listed below:

Module 1-F AAGGCTCGAGgagcacagtgttggcattaag XhoI

Module 1-R CCGTGAGCTCAGAACCGACACCAAATGACATC SacI

Module 2-F AAGGCTCGAGGATGTCATTTGGTGTCGGTTCT XhoI

Module 2-R CCGTGAGCTCACCAACAAACAATTCAGTGACC SacI

Module 3-F CCGTGAGCTCGAGAACCCTTGAAATGAGGC SacI

Module 3-R CTAAGTCGACTAAGTTTTGGTGTGCTGCTCAC SalI

Module A-F CTAAGTCGACACAAGGGTTACGGGTCTTACG SalI

Module A-R TACAGGTACCGGCAATATGATGTCACACCTAC KpnI

Module B-F AAGGCTCGAGaccctaagcggagcgaata XhoI

Module B-R CTAAGTCGACAATTGTTCTGGAAGACTCACCA SalI

Module C-F AAGGCTCGAGgcttggcagtgtgtaatgacc XhoI

Module C-R CTAAGTCGACGTCCTCAATACCGCTGAACG SalI

Module D-F AAGGCTCGAGATTGCGAAGTCTTTGTCAGT XhoI

Module D-R CCGTGAGCTCCCTTTGTGTTCTTTAAGCAAGT SacI

Basal promoter-F AATTCCCGGGATAAACAAGTATGGGCTACGC XmaI

Basal promoter-R TTCAGGTACCGACCTGAGAGAGAGTGAAAT BamHI

Module 4-F TACAGGTACCTGGACTCACTCCTACACAGGT KpnI

Module 4-R AATTCCCGGGACACTGGTTGTTGGACTAGCC XmaI

Module 5-F AAGGCTCGAGAACACTTCGTCCAGAGCCTAA XhoI

Module 5-R CCGTGAGCTCAGTTCCGACTGTTGTCCTAGC SacI

Module 6-F CTAACTCGAGATTCGCTTTTGAAGGAGTACCG SalI

Module 6-R TACAGGTACCCCAGTCGTCAGTATTTGTAAGAC KpnI

3087-F CTAAGTCGACACAAGGGTTACGGGTCTTACG SalI

1562-Bp-F AAGGCTCGAGgcttggcagtgtgtaatgacc XhoI

1060-F AAGGCTCGAGATTGCGAAGTCTTTGTCAGT XhoI

898-Bp-F acttgcttaaagaacacaaagg

749-Bp-F agaacaagtcccacaataag

681-Bp-F AAGGCTCGAGGCCAATGATGAAGTAACAGGT XhoI

448-Bp-F CAGGAATGTGAGTTCATAACAA

B:C-Bp-F AAGGCTCGAGaccctaagcggagcgaata XhoI

B:C-Bp-R CTAAGTCGACGTCCTCAATACCGCTGAACG SalI

1060△42-Bp-F aacagagcagtgaaagctagggatgctaattacttgctta

A-F CTAAGTCGACACAAGGGTTACGGGTCTTACG SalI

A462-F tcacttggttcgcagagaa

A433-F atgtcgctaacaagccgtgc

A415-F gcaaatgcagtgattggaca

A392-F CCCGTATAACTCCCCAGACC

A315-F GCATCTCTGTTGAAAATGTTGC

A△372-R TACAGGTACCGGTCTGGGGAGTTATACGGG KpnI

A△342-R TACAGGTACCGTGTGGTCTCATTAAGTGGG KpnI

A△298-R TACAGGTACCATTTTCAACAGAGATGCCCC KpnI

A462△298-Bp-M agagaacCCTCATTAAATGTGCCGTGCAAATGCAGTGATT
Six3a-C2-22bp-F atcgagatctcaggatgcccacttaatgagacggatcctacg

Six3a-C2-22bp-R cgtaggatccgtctcattaagtgggcatcctgagatctcgat

D184-F AAGGCTCGAGATTGCGAAGTCTTTGTCAGT XhoI

D141-F cgttcagcggtattgaggac

D105-F ggcatagaacagagcagtga

D69-F cagacgattaaaccaggtga

D30-F tgctaattacttgcttaaag

D28-F atcgagatcttgctaattacttgcttaaagaacacaaaggatcctacg

BglII BamHI

D-28-R cgtaggatcctttgtgttctttaagcaagtaattagcaagatctcgat

BamHI BglII

D-28M-F atcgagatcttgAGCCGGacttgcttaaagaacacaaaggatcctacg

BglII BamHI

D-28M-R CGTAGGATCCTTTGTGTTCTTTAAGCAAGTCCGGCTCAAGATCTCGAT

BamHI BglII

D△74-R TACAGGTACCCCTAGCTTTCACTGCTCTGT KpnI

D△30-R CCGTGAGCTCTCCCCAAATAAGTTCATTCACC SacI

EMSA oligonucleotides:

D184: PCR using D-F and D-R primers, and digested with XhoI.

D184△74: PCR using D-F and D△74-R primers, and digested with XhoI.

A462△298: PCR using A462-F and A△298-R primers, and digested with XhoI.

D69 probe was generated from PCR using: D69-F and D-R primers, and digested with *Xho*I and *Sac*I to get 5'protruding end for labelling.

D-F AAGGCTCGAGATTGCGAAGTCTTTGTCAGT XhoI

D-R CCGTGAGCTCCCTTTGTGTTCTTTAAGCAAGT SacI

D-F AAGGCTCGAGATTGCGAAGTCTTTGTCAGT XhoI

D△74-R TACAGGTACCCCTAGCTTTCACTGCTCTGT KpnI

D69-F AAGGCTCGAGcagacgattaaaccaggtga XhoI

D-R CCGTGAGCTCCCTTTGTGTTCTTTAAGCAAGT SacI

A462-F AAGGCTCGAGtcacttggttcgcagagaa XhoI

A△298-R TACAGGTACCATTTTCAACAGAGATGCCCC KpnI

Double strand oligonucleotide

Bp2-28-GS-F AGCTTGCTAATTACTTGCTTAAAGAACACAAA

Bp2-28-GS-R TTTGTGTTCTTTAAGCAAGTAATTAGCA

D#1-F AGCTATTGCGAAGTCTTTGTCAGTAAATAAACAA

D#1-R TTGTTTATTTACTGACAAAGACTTCGCAAT

D#2-F AGCTAACAAAGACGGAGCTAAACGTTCAG

D#2-R CTGAACGTTTAGCTCCGTCTTTGTT

D#3-F AGCTTTCAGCGGTATTGAGGACCTCGCTC

D#3-R GAGCGAGGTCCTCAATACCGCTGAA

D#4-F AGCTCGCTCCCTGCTGATTTTGGCATAGA

D#4-R TCTATGCCAAAATCAGCAGGGAGCG

D#5-F AGCTATAGAACAGAGCAGTGAAAGCTAGG

D#5-R CCTAGCTTTCACTGCTCTGTTCTAT

## Oligonucleotide for competition:

D69-F AAGGCTCGAGcagacgattaaaccaggtga XhoI

A462-F AAGGCTCGAGtcacttggttcgcagagaa XhoI

D#4-(Pax)m-F ATAGAAATGCTGATTTTGGCATAGA
D#4-(Pax)m-R TCTATGCCAAAATCAGCATTTCTAT

D#5-(Fox)m-F AGCTCCACGAGCAGTGAAAGCTAGG
D#5-(Fox)m-R CCTAGCTTTCACTGCTCGTGGAGCT

Bp2-28-(POU)m-F TGAGCCGGCATTGCTTAAAGAACACAAA
Bp2-28-(POU)m-R TTTGTGTTCTTTAAGCAATGCCGGCTCA

Bp2-28-(Hox)m-F TGCTTTTTACTTGCTTAAAGAACACAAA

Bp2-28-(Hox)m-R    TTTGTGTTCTTTAAGCAAGTAAAAAGCA

## 
