# Supplementary material for: Complexity of cis-regulatory organization of six3a during forebrain and eye development in zebrafish
Source: BMC Dev Biol. 2010 Mar 26;10:35. doi: 10.1186/1471-213X-10-35 (PMC2858731; doi:10.1186/1471-213X-10-35)
Supplement: Additional file 4 — Conserved non-coding regions or regulatory modules identified in this study. All of the conserved non-coding regions or regulatory elements identified in this study are shown. [file 1471-213X-10-35-S4.DOC]

## Additional file 4 – Conserved non-coding regions or regulatory modules identified in this study

All the conserved non-coding regions or regulatory elements identified in this study are shown below:

**Module 1 (-10890~-9919) 972 bp**

gagcacagtgttggcattaagtcagcgtgtcagtttacaaaaacaaaaaatacagtccacttatttctgtttgaccattactttgaggtttttcagagtgacagcggaagactttgaaacaagtgttgaacataagcatatctctgtagataaaaaaaaaaatgtataaacaggctgagaagatttttgctcaagtgtcttggcttttaaaagcagtctatctgtttcatggcgatagagttccgaatagaataacgtgtaattaatgttgggatgtggccctggggagcttggaaatgttggaagaggagagaggagggagaggagtcacggagatttctgattacctgacaagttggggccggaaaggctacacttagcccattaaagattttccagcgtgacatttgagagcagctgccgcaccgtgcgccacctcgccatttccccattcgctgggcatcaatttagagagagaaagagagagagagaaggggagagaaaggaagagcaaacgagagaaagagaggccaccaggcagacactggcacaaatggatttcattcaccaaggtgaactgtacttggctgataaatgcacttccttgaatgtcagcaaatgtcatttgtgaagccttttactgttgctttaatagcttccccaaacttacaaagaaatgaaaatagaatgacagagagagtgagagaacagagagagagagagggtttggctgttgtcacttccacatgattaagcaataagacatttgagaagtcccaacaagaaagtggttaaacttgctggataatgcctccgcgattaagtgttagactgtgagtttcttgctttgaactgaagctttggacaaaagcttgggaaaacccagttcaatccagttactaatcaaatcatagatcagctttggtttttaaaaatgttccacaacacaagatgtcatttggtgtcggttct

**Module 2 (-9940~–9361) 580 bp**

GATGTCATTTGGTGTCGGTTCTtcttttaaaggtggcagcaaaagtttgccattgacattaaacacaaaatgtaataggtaaattaatacaaaatgcttttcaacttaaaaacaaaatcatgaattatggatgaattcttcttgtataaacactcatattagaataaaattatagaagaggctgatttaacatcaatgatttgccagtatctataatataaattcctctaaatacattataatttaaaaaggtgtgtgacactttatgattatttaggaatcctgttgtatcctgggcaacctcactctggcccaatccacaaaggcagcccacatggctgtgataatccccatttccagtagttgctaaggttacaaattacctttggtactcttaacagctcctctgtgacactctactaatccttttatggaatttcatttaggatttatactttagatacaggattacagagcactatattttgttattgttcacatttgtccagtggagtcacggagcacgttccagtctcttttattttcagcttagacactggtcactgaattgtttgttggt

˙

**Module 3 (-7301~-6377) 925 bp**

gagaacccttgaaatgaggcctaacttagaatcgcacatataacctgagccattttttcatgctggcactatccacactagtcattacccagaaaagtagataatcatttggatgttttggggggtatttatctattcatgtcggcgaatgataaccatcacagccattgaaagaccaagggcagactaggctacaggttacagctgtcagtcataaattttttatttgggcatgggataatgcgtcgtgcactgggcttactgggcaaatgttattcactcataaacatttttaagacgttttgacatctgcttgagtttttgataatactttagttaagtgatctaataaaaaattaatatgcattgatatgacaagtatatcggtcaaatatttgaacagaccctataaattaatgtttcagtaaattcgtatgctataattgcatttgtttttggcaatgtgttttagtgttattattattttatttatattaatgtattttagtagtattgccatctaaacaagaaggaaacgtgcattggaattcatttaattcattttggattgaaaacacaaagacactgaatacacagactggctggagtcttatcacaagcttaattttaaaacattatgaaccatttacactaaaataataattcactggttacaatttacttttacaatctacttttatgaaaatattttttgcaaaacaaaggttctagataagaaacagatttaaaaaataatgttttttagcctctttttgttaatgctaactgaataataataataataataataataataataataataataataataataacaataatataaaaaagactaaaaatttcgtcctattttctattcgtttaaattatttcttttggtgagcagcacaccaaaactta

**Module A (-3781~-3119) 773 bp (medaka six3.2-Box A shown in yellow highlighted)**

acaagggttacgggtcttacgttgatcctgaatagggtcttaaaatcaacataatcaattaaacagaatggatacaacgtatgtctctttataagaatatctcgctactccctccaaccccaaaacacagtgcacgtcatcaccacccacaccacacagtatggatttggtccaagccaaagcactaagagaaaccggaagaaagagggcctgcagttccaggcgtccggctccattcactagcgaaacctccaatggcttcattagaagtaacgagttgacacaaggtaaatctaacattgaagcgccattcacttggttcgcagagaaccctcattaaatgtcgctaacaagccgtgcaaatgcagtgattggacagctcccgtataactccccagacctagcaggatgcccacttaatgagaccacacaaaaagggggggtggtggtggtggggggcatctctgttgaaaatgttgctagaatgtcgagtccctcccccggcaaacaagaaaatatatacatatacaaaaatgttgtatagctaatgcaaataatgaattaacaacccccctgcgttaaataggcctaataaaatgctaaatttagtaagtcatgaggattttgatcatgaacatgatccgcaaaaatagttaaaaatataattctttaacaaataaaacatatctaaaacttgtcactcaaaacatttttacattttccagaaatggtatcttggctacctacccaagtaggtgtgacatcatattgcc

**A462**

tcacttggttcgcagagaacCCTCATTAAATGTCGCTAACAAGCCGTGCAAATGCAGTGATTGGACAGCTCCCGTATAACTCCCCAGACCTAGCAGGATGCCCACTTAATGAGACCACACAAAAAgggggggtggtggtggtggggggcatctctgttgaaaatgttgctagaatgtcgagtccctcccccggcaaacaagaaaatatatacatatacaaaaatgttgtatagctaatgcaaataatgaattaacaacccccctgcgttaaataggcctaataaaatgctaaatttagtaagtcatgaggattttgatcatgaacatgatccgcaaaaatagttaaaaatataattctttaacaaataaaacatatctaaaacttgtcactcaaaa….

**A433-F**

ATGTCGCTAACAAGCCGTGCAAATGCAGTGATTGGACAGCTCCCGTATAACTCCCCAGACCTAGCAGGATGCCCACTTAATGAGACCACACAAAAAgggggggtggtggtggtggggggcatctctgttgaaaatgttgctagaatgtcgagtccctcccccggcaaacaagaaaatatatacatatacaaaaatgttgtatagctaatgcaaataatgaattaacaacccccctgcgttaaataggcctaataaaatgctaaatttagtaagtcatgaggattttgatcatgaacatgatccgcaaaaatagttaaaaatataattctttaacaaataaaacatatctaaaacttgtcactcaaaa….

**A415-F**

GCAAATGCAGTGATTGGACAGCTCCCGTATAACTCCCCAGACCTAGCAGGATGCCCACTTAATGAGACCACACAAAAAgggggggtggtggtggtggggggcatctctgttgaaaatgttgctagaatgtcgagtccctcccccggcaaacaagaaaatatatacatatacaaaaatgttgtatagctaatgcaaataatgaattaacaacccccctgcgttaaataggcctaataaaatgctaaatttagtaagtcatgaggattttgatcatgaacatgatccgcaaaaatagttaaaaatataattctttaacaaataaaacatatctaaaacttgtcactcaaaa….

**A392-F**

CCCGTATAACTCCCCAGACCTAGCAGGATGCCCACTTAATGAGACCACACAAAAAgggggggtggtggtggtggggggcatctctgttgaaaatgttgctagaatgtcgagtccctcccccggcaaacaagaaaatatatacatatacaaaaatgttgtatagctaatgcaaataatgaattaacaacccccctgcgttaaataggcctaataaaatgctaaatttagtaagtcatgaggattttgatcatgaacatgatccgcaaaaatagttaaaaatataattctttaacaaataaaacatatctaaaacttgtcactcaaaa….

**A315-F**

gcatctctgttgaaaatgttgctagaatgtcgagtccctcccccggcaaacaagaaaatatatacatatacaaaaatgttgtatagctaatgcaaataatgaattaacaacccccctgcgttaaataggcctaataaaatgctaaatttagtaagtcatgaggattttgatcatgaacatgatccgcaaaaatagttaaaaatataattctttaacaaataaaacatatctaaaacttgtcactcaaaa….

**Module B (-2932~-2642) 291 bp (putative brn3-b binding site, Brn3 binding site in green highlighted, medaka six3.2-Box B shown in yellow highlighted):**

accctaagcggagcgaatagaaaggggcatttgcgccccgttacgacggccccacaacgctgcaagctgcacttgtctcgcgtgattcactgagtggagagcaacgtcccggcagaggatgacaactttaaaacagataaactgggaatatgcatgactctaagcggttctgaacgatgcttactgattaacctaccaccatcgcattatgatctacttggtgtcttttactaaagtcctaaatattcttaagaactcgaccaactctctggtgagtcttccagaacaatt

**Module C (-2256~-1692) 565 bp (putative Pax6.1 binding site, Pax6.1 binding site in blue highlighted, medaka six3.2-Box D shown in yellow highlighted)):**

gcttggcagtgtgtaatgacctaagtggatgtataaaaacagaaaaagcgtttaaaataaaataaaatcttaaacgggagaaggcattgtcccgtgccctttccttctcaaacacttgcaagtccccgactcaccacacaatgcacaaaattactcatgcatgttaaccgcgtagttgccaacatttggagagaggatgggaggacacgttttagtgtgataagaagtgagtgcaagtttgaatcgagagctaagaaatggtttacttaccttctcatacgaccaagcttttctcagtgatctctggcatgccgtaacaaaacgtatagctttatttgggctatatgactctctcagagagaataatagtcgaaattacttgttaactttaaatcgtctaatgtcagatcaattagaaatagcctactacaattttgacgaaaacaaatatacatattctatacgttgaactgcgagataatccactggctattactttcttattgcgaagtctttgtcagtaaataaacaaagacggagctaaacgttcagcggtattgaggac

**Module B:C (-2932~-1692) 1240bp (Brn3 binding site in green highlighted, Pax6.1 binding site in blue highlighted)**

accctaagcggagcgaatagaaaggggcatttgcgccccgttacgacggccccacaacgctgcaagctgcacttgtctcgcgtgattcactgagtGGAGAGCAACGTCCCGGCAGAGGATGACAACTTTAAAACAGATAAACTGGGAATATGCATgactctaagcggttctgaacgatgcttactgattaacctaccaccatcgcattatgatctacttggtgtcttttactaaagtcctaaatattcttaagaactcgaccaactctctggtgagtcttccagaacaattagctattttaaaataacatgcttcaaacttataggatgcaaagacgcatgctacacacttgaagcagcacagagctcatggacacatttcaaacaagccgtttcagacggatttgtttttatcgcagtagaaaacagcttgtctctaattttataaatgaatgaaagcaagtaaaacgctgaaaaaaacaaacgaataaaaatgtaatgtaagggcaaaatgagtgaacaatggatcagaatcgatagaacaacaaagtttcgaaaatcacaaagttagtttaagaaaaaaaaaggaaaaagaaagactcaaaccatcgcttcgctccggagaagaagtctcttgatagaactgtctttcgccgtcacatgaagatcgatttcctgcttggcagtgtgtaatgacctaagtggatgtataaaaacagaaaaagcgtttaaaataaaataaaatcttaaacgggagaaggcattgtcccgtgccctttccttctcaaacacttgcaagtccccgactcaccacacaatgcacaaaattactcatgcatgttaaccgcgtagttgccaacatttggagagaggatgggaggacacgttttagtgtgataagaagtgagtgcaagtttgaatcgagagctaagaaatggtttacttaccttctcatacgaccaagcttttctcagtgatctctggcatgccgtaacaaaacgtatagctttatttgggctatatgactctctcagagagaataatagtcgaaattacttgttaactttaaatcgtctaatgtcagatcaattagaaatagcctactacaattttgacgaaaacaaatatacatattctatacgttgaactgcgagataatccactggctattactttcttATTGCGAAGTCTTTGTCAGTAAATAAACAAAGACGGAGCTAAACGTTCAGCGGTATTGAGGAC

**1060-Bp ~ 898-Bp (part of B:C-Bp underlined, gel shift probe #4 in green highlighted, gel shift probe #5 in blue highlighted, deletion in 1060△42-Bp shown in italic and bold)**

ATTGCGAAGTCTTTGTCAGTAAATAAACAAAGACGGAGCTAAACGTTCAGCGGTATTGAGGACCTCGCTCCCTGCTGATTTTGGCATAGAACAGAGCAGTGAAAGCTAG***GGGATAAACAGACGATTAAACCAGGTGAATGAACTTATTTGG***GGATGCTAATT

**most important sequence in 1060~898**

CTCGCTCCCTGCTGATTTTGGCATAGAACAGAGCAGTGAAAGCTAG

**681-Bp ~ 448-Bp (Late neural and retina enhancer medaka six3.2-Box C shown in blue highlighted)**

gccaatgatgaagtaacaggTTATTTCCTGTCCAAAAAGCTTTCCAAAGGGACCATTTCAAGTGCCACTTGGAATTACTTCACTTAAGTTTCCTGTGACAGAAGAACAAACAGATTTGATCCTGGAAAcacaacgtgacccaatacttctataaagacctcttccccgcccctttctgccacacacacacgagcttgcagtgcatgtgtaaatctatgacagtgtaatcagaa

Late neural and retina enhancer Box E

**Module D (-1754~-1571) 183 bp (medaka six3.2-Box D shown in yellow highlighted):**

ATTGCGAAGTCTTTGTCAGTAAATAAACAAAGACGGAGCTAAACGTTCAGCGGTATTGAGGACCTCGCTCCCTGCTGATTTTGGCATAGAACAGAGCAGTGAAAGCTAGGGGATAAACAGACGATTAAACCAGGTGAATGAACTTATTTGGGGATGCTAATTACTTGCTTAAAGAACACAAAGG

**Basal promoter (-694~+110) 804 bp (medaka six3.2-Box I shown in blue highlighted, medaka six3.2-Box L shown in yellow highlighted)**

ataaacaagtatgggctacgcgccagtggtttgctccgagcaaaaaagaaaagcaaagcgcagccacgggaatgcgcgacggttgtcactgtgagaatagcggtcggtcacatcgtcgagacgcgtttgtaatgcaaaacgttccgggagctagccacaaatacgcgcatgcttttaaatacacaattcaaacgcgttttgcggctgctgtattcatgagataagcgacacggcgtaacatcgtagcatatcttctctagtggtgctaaagaatgaaatagcctatctgtgcacctatagtagattccgttttagcgcttcatttaaattcccgtgtccgtgacagtcatcccgagaccccacccctgccttctctcccTACATATCTTCTTAACTTTAACGAGCCTCGTTAAGATCACAATAATATTCCACCCACTAATTGCTCATTCCATTCAACAAATAGGCGAGAGTCTGCTTCGACTTCACACGAGTCAAGGGAGGGAGGGAGCTGTTGAGATTGGAGTTCCCGATAACCCCCCGTGCgtGCAGCCGAAGTGGTGAAAGCCTCTACGTACTGGCTAATGATTGGCACGCTTGACAGTGATTGGCAGGGCTGCCATGACAACCCTACAACGACACCGAGAAGACCAATAGAAAAGCGAAACAAAATATTTCAATGCTACACTCACGGTGG**ATTTAGGGGGAGATATTATGAGGCTGGTGTCATTAGGCGATAGCTATTGAATCATTCAATCTGAATTCGTCGTTCTTTTTTCCTTCGCAAATTTCACTCTCTCTCAGGTC**

**Module 4 (+730~+2409) 1800 bp**

tggactcactcctacacaggtcggaaattggtttaaaaacaggagacaacgagacagggcagcggcagcaaaaaacaggtcagtcatgttatgtattgatacggttctacagaaattgtgtgcttcaacagtgatattaaatgtatacttattaatgatctgttgggaaatacataaacacaacgtaggaagtgatgcagttaaaacaaaatcaaatagattataacgaatacaacaaatgagggagcgaaaacgacggctttttagttgtgtaggctaaaatgtttagaaaggcttaaccaaacactttaagcaagatagaactatttaatcgttttccttaaactataaatataagctaataaaaaatgtttcctctgattaaattttacataattaaaataacacataatcaaacgtgtttatgcttctcaacacagatatgctgagttataaaatagccttttatagtcttagtcatgtgaaggtttctattttaatttactaagctttggtcgtaaattcacaatacatttttgcataatacaaaagaatagatgtggataactcctaaatttagaaatataaaaaaaatacttatagcatttctctttaagattttaaagatattttaatttatttatgaatagatttatatttcttatattgtaaccaattaattctaacaagtaatttatcacaaacattcttttcaacagatttgctacatgataatgtaaatacaattttttttaaatatacatacacagcatactctgataaataaatcacaattataaataaaattgtttgtggtacattttgcaaaggactattatgttctatgactgttaaatctgtaatctttttatttgtattttgattttaaattgtattgtgtgtgtgcgtatgtatgtatgtatgaatgtgtgtgtgtgtgtgtgtgtatatatatatatatatatatatatatatatatatatatatatatatatatatatatatattactgtgtatcttgggaacgaaaaagcacatttaactatggaactgatttatatcatttaatatcattgtatcatttaatgcatagatctacaaaaagcacttgactggataattacacatcgccaaaaaaacaaaaaacaaatgaactatgagatttttgttcatgtataacatagcaagactaatcttcatctgacgtcagctgatttgaacatgtaaacatacaaatcggactaatacttaattgtaccattgcaagttaaaaaataatctagcaagtcaatcaaatctcgacgacagtctatgagcatgctattattttggtatgtagactaattcttgtagcgaccgtgcattgtgtataaccgctttccaaaaatgtagctgtagcatttccttcaagtagttttaaattagcaagttgaactgtcgctttatttaagatatctcgtttctattaaaaatgctgtttaacagagaaggtgcatttaggtggagaaaatggcctaggaatttttaatttaaataacataccactctcttatttttctgctataggctccagcatcaagcaatagggcagaatggcatgcggtccctttcagaatccggctgcaccccacggagttcggcggagtcgccatcaactgcggctagtccaacaaccagtgt

**Module 5 (+4730~+5161) 432 bp**

aacacttcgtccagagcctaaaaggtgtcaaaaaggcagaatacattttttgtcctgttcccataaccttagtcgcacagagacaaatgaatgcaattggaccgcagacggcaataagaaaagattgtacagccctgttttttctctgatttcatcggttaactgaacacaacttttctattcgtgtgtattcatgcagttctcaatgagcgtcagttccctctgacccggattgaaagcgtgcgcctcggcatagcctgtcagtgcaacccctcttggcaagactgggccttttccctcccgacacagtccccttttcacaaataagcactgagtaaggtacgcggagcaagaatccactatgtgcattagtagcttacatatcgcgacagtggctagctgtgcattagtgctaggacaacagtcggaact

**Module 6 (+5748~+6083) 336 bp**

attcgcttttgaaggagtaccgcgagtgttgttcccgacccagcagcctgtgatgaggcgttcttggcaagcaggattaaaaactgtaattctattacagagtaatttagaggtcacaagaggctatgatacaggatcagcagtaagatcagagtgtaaaagctcgtttgaaggttaattcggatgagatcccatgaactgtggaaaaactatagaaataatcaggttttgttctaccttcacaccttgtaaggttgcagaagtctctgccgaaacatttacataagctacgagtggatgctaaacgaaaagagtcttacaaatactgacgactgg

Medaka

Box A:

CCTCATTAAATGCCGCTAACAACCCGTGTAAATACACTGATTGGACAGCTCCGATACAAGCTCCAAGCCC TGCCAGGAAGTCCCCCTAATGAGACCACACAATCAAAAACAATAAA

Box B:

GGATTGCACCGTCCCGATAGCATCACACATCTGCTCTGCA GAGAAAAGATCTAGTGGGTG

Box C

TCGGTTGGATCCAGGATGGAGCAGCAGCACATGAGTCTCCGCTCTGTTTGCCAGAGAGGGGTGGGCTGCCTTTTCTGGCCAGGCTCTGCCAAACCGAGGTCATCGAGGGAGCAAAAACTGTAACAAAACAAGAATTGCGCCGTCTTTCTCTTCCCTCTTACCTTTTCCAGTCGACACCTTTTCTTTCTATCCTCGTGCGCTCCCCTTCTCTCTCTCACTCTTATAAATGATTTGCTCCCCTTCTAAGAACAGACTG

Box D

GTGTTGTTGCGTCTGGAGATTTAGGCTGTGAGTAAACGAAGAAGACGCTAAATGCTCCGTGCGCATTCAGAGCTCCCGTCCCCCGCTGCTAGGAACATTCAGCAAGCTCTCGGATAAACACACAATTACGGCAGGTGAATAAACTTGTTTGGGGAGTGTTAATTACGTGCTTGACGTAAACAAAAGCCATGTAAAAGAATGTGAATTAGCTGTGGCTTTCCTGCCTCAATC

Box E

TACCAGCCTTTACTTCCAGTCCAATGAGCTTTCCAATGGGACCAAATCAAGTGCCACTTAGAATTAGTCCTCCTGAATTTTGTGCGGCAGCACAGTGGACAGGTTTGATCCC

Box G

AAAGTTTTGAATTCCTGAATGAATTTGTAGAAAGGCGCTTAGGATCATTATGTCAGCTTTATGGAGGACGGATTAAGCGGAGTCTAGATGCTACGCCTTGTACACGGTTCTCTCACAATGTGATCACTGTTCGTTTTATAGTGAACCCACACTATTTTAACACCTTGCAAACCCTGTCTAAACATGAAACAGTTTCAGCCATAAACATGAAAATCACACCGCTTTGGCTG

Box H

TAAGCCAAATCCTGTGACTGGATGCATGGGGACATTGATGTTGTCCTTATAGCCTCAGATCAAATGCAAGAAACTGCTTTTATGTGCCTAGTATCTTTTATGTTGCCATTCGATTGTAAT

Box I

GCATCTCCAGTCTACATATCTTCTTTAGCTTTAACGAGCCTCGTTAAGATCGCAATAATATTCCACCCTCTAATTGCTCATTCCATTCAGCAGATAGGCGAGCATTGGCTTGTGCCTGATGCGCGCGGTGCGGTGGGAGGGTTGCTGTGGAGATCCTAGACTCTGATAACCCCCCGTGC

Box L

GCTGCACAAGTGGTGAAAGCCTCGCGCTACGTACTGGCTAATGATTGGCACGCTTGACAGTGATTGGCAGGGCTGCCATGACAACGCTACAACGACACCAAGAAGACCAATAGAAAAGGGAAACAAAATGTTTCAATGCTACACTCAACGGCGGATTTAGGGGGGAGATATTATGAGGCTGGTGTCATTAGGCGATAGCCATTGAATCATTGAATCTTTTTTACTTACCGTATTTTTC

**805~236 upstream of ATG in Wargelius et al (2003) (Box I shown in blue highlighted, Box L shown in grey highlighted)**

gtatgggctacgcgccagtggtttgctccgagcaaaaaagaaaagcaaagcgcagccacgggaatgcgcgacggttgtcactgtgagaatagcggtcggtcacatcgtcgagacgcgtttgtaatgcaaaacgttccgggagctagccacaaatacgcgcatgcttttaaatacacaattcaaacgcgttttgcggctgctgtattcatgagataagcgacacggcgtaacatcgtagcatatcttctctagtggtgctaaagaatgaaatagcctatctgtgcacctatagtagattccgttttagcgcttcatttaaattcccgtgtccgtgacagtcatcccgagaccccacccctgccttctctcccTACATATCTTCTTAACTTTAACGAGCCTCGTTAAGATCACAATAATATTCCACCCACTAATTGCTCATTCCATTCAACAAATAGGCGAGAGTCTGCTTCGACTTCACACGAGTCAAGGGAGGGAGGGAGCTGTTGAGATTGGAGTTCCCGATAACCCCCCGTGCgtGCAGCCGAAGTGGTGAAAGCCTCTACGTACTGGCTAATGATTGGCACGCTTGACAGTGATTGGCAGGGCTGCCATGACAACCCTACAACGACACCGAGAAGACCAATAGAAAAGCGAAACAAAATATTTCAATGCTACACTCACGGTGG**ATTTAGGGGGAGATATTATGAGGCTGGTGTCATTAGGCGATAGCTATTGAATCATTCAATCTGAATTCGTCGTTCTTTTTTCCTTCGCAAATTTCACTCTCTCTCAGGTCATTTCCATG**
