# Supplementary material for: Complexity of cis-regulatory organization of six3a during forebrain and eye development in zebrafish
Source: BMC Dev Biol. 2010 Mar 26;10:35. doi: 10.1186/1471-213X-10-35 (PMC2858731; doi:10.1186/1471-213X-10-35)
Supplement: Additional file 5 — Raw data for microinjection experiment. All of the microinjection data in this study are shown. [file 1471-213X-10-35-S5.DOC]

## Additional file 5- Raw data for microinjection experiment

| 1-Bp | 8 hpf | total-8hpf | 11 hpf | total-11hpf | 24 hpf | total-24hpf |
| --- | --- | --- | --- | --- | --- | --- |
| batch 1 | 0 | 38 | 0 | 38 | 5 | 31 |
|  | 0 | 38 | 0 | 38 | 5 | 31 |
|  |  |  |  |  |  |  |
| 2-Bp | 8 hpf | total-8hpf | 11 hpf | total-11hpf | 24 hpf | total-24hpf |
| batch 1 | 3 | 28 | 3 | 28 | 15 | 20 |
| batch 2 | 7 | 31 | 3 | 28 | 19 | 19 |
| batch 3 |  |  |  |  | 19 | 50 |
| batch 4 |  |  |  |  | 43 | 60 |
| batch 5 |  |  | 0 | 30 | 12 | 31 |
| batch 6 | 0 | 52 | 0 | 50 | 14 | 48 |
| batch 7 | 0 | 30 | 0 | 30 | 9 | 20 |
| batch 8 |  |  |  |  | 2 | 23 |
|  | 10 | 141 | 6 | 166 | 131 | 248 |
|  |  |  |  |  |  |  |
| 3-Bp | 8 hpf | total-8hpf | 11 hpf | total-11hpf | 24 hpf | total-24hpf |
| batch 1 | 0 | 35 | 0 | 35 | 4 | 34 |
| batch 2 | 0 | 48 | 0 | 46 | 1 | 41 |
|  | 0 | 83 | 0 | 81 | 5 | 75 |
|  |  |  |  |  |  |  |
| A-Bp | 8 hpf | total-8hpf | 11 hpf | total-11hpf | 24 hpf | total-24hpf |
| batch 1 | 0 | 65 | 0 | 63 | 40 | 51 |
| batch 2 | 0 | 35 | 0 | 33 | 25 | 29 |
| batch 3 | 0 | 72 | 0 | 70 | 63 | 66 |
| batch 4 | 0 | 40 | 0 | 38 | 29 | 31 |
| batch 5 |  |  |  |  | 35 | 43 |
| batch 6 |  |  |  |  | 56 | 68 |
| batch 7 |  |  |  |  | 10 | 14 |
| batch 8 |  |  |  |  | 32 | 35 |
|  | 0 | 212 | 0 | 204 | 290 | 337 |
|  |  |  |  |  |  |  |
| B-Bp | 8 hpf | total-8hpf | 11 hpf | total-11hpf | 24 hpf | total-24hpf |
| batch 1 | 0 | 40 | 0 | 38 | 6 | 33 |
|  | 0 | 40 | 0 | 38 | 6 | 33 |
|  |  |  |  |  |  |  |
| C-Bp | 8 hpf | total-8hpf | 11 hpf | total-11hpf | 24 hpf | total-24hpf |
| batch 1 | 0 | 39 | 0 | 39 | 3 | 38 |
| batch 2 |  |  |  |  | 8 | 11 |
|  | 0 | 39 | 0 | 39 | 11 | 49 |
|  |  |  |  |  |  |  |
| D-Bp | 8 hpf | total-8hpf | 11 hpf | total-11hpf | 24 hpf | total-24hpf |
| batch 1 | 58 | 60 | 58 | 58 | 52 | 52 |
| batch 2 | 46 | 48 | 39 | 44 | 39 | 44 |
|  | 104 | 108 | 97 | 102 | 91 | 96 |
|  |  |  |  |  |  |  |
| 4-Bp | 8 hpf | total-8hpf | 11 hpf | total-11hpf | 24 hpf | total-24hpf |
| batch 1 | 0 | 50 | 0 | 50 | 8 | 38 |
| batch 2 | 0 | 40 | 0 | 40 | 4 | 39 |
|  | 0 | 90 | 0 | 90 | 12 | 77 |
|  |  |  |  |  |  |  |
| 5-Bp | 8 hpf | total-8hpf | 11 hpf | total-11hpf | 24 hpf | total-24hpf |
| batch 1 | 0 | 42 | 0 | 42 | 1 | 36 |
|  | 0 | 42 | 0 | 42 | 1 | 36 |
|  |  |  |  |  |  |  |
| 6-Bp | 8 hpf | total-8hpf | 11 hpf | total-11hpf | 24 hpf | total-24hpf |
| batch 1 | 0 | 43 | 0 | 43 | 0 | 40 |
| batch 2 | 0 | 36 | 0 | 33 | 1 | 20 |
|  | 0 | 79 | 0 | 76 | 1 | 60 |
|  |  |  |  |  |  |  |
| Bp | 8 hpf | total-8hpf | 11 hpf | total-11hpf | 24 hpf | total-24hpf |
| batch 1 | 0 | 63 | 0 | 63 | 3 | 63 |
|  | 0 | 63 | 0 | 63 | 3 | 63 |
|  |  |  |  |  |  |  |
| 3087-Bp | 8 hpf | total-8hpf | 11 hpf | total-11hpf | 24 hpf | total-24hpf |
| batch 2 | 68 | 70 | 67 | 69 | 8 | 8 |
| batch 3 | 63 | 65 | 61 | 63 | 59 | 61 |
| batch 4 | 78 | 78 | 74 | 75 | 69 | 72 |
|  | 209 | 213 | 202 | 207 | 136 | 141 |
|  |  |  |  |  |  |  |
| 1562-Bp | 8 hpf | total-8hpf | 11 hpf | total-11hpf | 24 hpf | total-24hpf |
| batch 2 |  |  |  |  | 103 | 105 |
|  | 0 | 0 | 0 | 0 | 103 | 105 |
|  |  |  |  |  |  |  |
| 1060-Bp | 8 hpf | total-8hpf | 11 hpf | total-11hpf | 24 hpf | total-24hpf |
| batch 1 | 46 | 49 | 45 | 47 | 43 | 45 |
| batch 2 |  |  |  |  | 50 | 50 |
|  | 46 | 49 | 45 | 47 | 93 | 95 |
|  |  |  |  |  |  |  |
| 1060△43-Bp | 8 hpf | total-8hpf | 11 hpf | total-11hpf | 24 hpf | total-24hpf |
| batch 1 |  |  |  |  | 92 | 92 |
| batch 2 | 27 | 33 | 27 | 33 | 29 | 36 |
|  | 27 | 33 | 27 | 33 | 121 | 128 |
|  |  |  |  |  |  |  |
| B:C-Bp | 8 hpf | total-8hpf | 11 hpf | total-11hpf | 24 hpf | total-24hpf |
| batch 1 | 0 | 61 | 0 | 59 | 2 | 52 |
|  | 0 | 61 | 0 | 59 | 2 | 52 |
|  |  |  |  |  |  |  |
| 898-Bp | 8 hpf | total-8hpf | 11 hpf | total-11hpf | 24 hpf | total-24hpf |
| batch 1 |  |  |  |  | 50 | 61 |
| batch 2 |  |  |  |  | 21 | 41 |
| batch 3 |  |  |  |  | 55 | 70 |
| batch 4 |  |  |  |  | 19 | 42 |
| batch 5 | 0 | 55 | 0 | 45 | 1 | 36 |
|  | 0 | 55 | 0 | 45 | 145 | 214 |
|  |  |  |  |  |  |  |
| 749-Bp | 8 hpf | total-8hpf | 11 hpf | total-11hpf | 24 hpf | total-24hpf |
| batch 1 |  |  |  |  | 19 | 43 |
| batch 2 |  |  |  |  | 26 | 56 |
| batch 3 |  |  |  |  | 42 | 59 |
| batch 4 | 0 | 45 | 0 | 45 | 0 | 42 |
|  | 0 | 45 | 0 | 45 | 87 | 158 |
|  |  |  |  |  |  |  |
| 681-Bp | 8 hpf | total-8hpf | 11 hpf | total-11hpf | 24 hpf | total-24hpf |
| batch 2 |  |  |  |  | 27 | 54 |
|  | 0 | 0 | 0 | 0 | 27 | 54 |
|  |  |  |  |  |  |  |
| 448-Bp | 8 hpf | total-8hpf | 11 hpf | total-11hpf | 24 hpf | total-24hpf |
| batch 2 | 0 | 33 | 0 | 30 | 1 | 27 |
|  | 0 | 33 | 0 | 30 | 1 | 27 |
|  |  |  |  |  |  |  |
| D105-Bp | 8 hpf | total-8hpf | 11 hpf | total-11hpf | 24 hpf | total-24hpf |
| batch 1 |  |  |  |  | 57 | 73 |
| batch 2 |  |  |  |  | 19 | 40 |
| batch 3 | 31 | 52 |  |  | 39 | 45 |
| batch 4 |  |  | 30 | 39 | 25 | 43 |
|  | 31 | 52 | 30 | 39 | 140 | 201 |
|  |  |  |  |  |  |  |
| D69-Bp | 8 hpf | total-8hpf | 11 hpf | total-11hpf | 24 hpf | total-24hpf |
| batch 1 |  |  |  |  | 52 | 65 |
| batch 2 |  |  |  |  | 26 | 45 |
| batch 3 |  |  | 5 | 40 | 25 | 63 |
|  | 0 | 0 | 5 | 40 | 103 | 173 |
|  |  |  |  |  |  |  |
| D28(2)-Bp | 8 hpf | total-8hpf | 11 hpf | total-11hpf | 24 hpf | total-24hpf |
| batch 1 |  |  |  |  | 38 | 50 |
| batch 2 |  |  |  |  | 33 | 35 |
| batch 3 | 20 | 90 | 93 | 111 | 92 | 105 |
|  | 20 | 90 | 93 | 111 | 163 | 190 |
|  |  |  |  |  |  |  |
| D28M(2)-Bp | 8 hpf | total-8hpf | 11 hpf | total-11hpf | 24 hpf | total-24hpf |
| batch 2 |  |  |  |  | 2 | 30 |
|  |  |  |  |  | 2 | 30 |
|  |  |  |  |  |  |  |
| D30-Bp | 8 hpf | total-8hpf | 11 hpf | total-11hpf | 24 hpf | total-24hpf |
| batch 1 |  |  |  |  | 18 | 48 |
| batch 2 |  |  |  |  | 37 | 39 |
|  | 0 | 0 | 0 | 0 | 55 | 87 |
|  |  |  |  |  |  |  |
| D184△30-Bp | 8 hpf | total-8hpf | 11 hpf | total-11hpf | 24 hpf | total-24hpf |
| batch 1 | 44 | 47 |  |  | 44 | 47 |
| batch 2 | 28 | 53 |  |  | 46 | 60 |
|  | 72 | 100 | 0 | 0 | 90 | 107 |
|  |  |  |  |  |  |  |
| D184△42-Bp | 8 hpf | total-8hpf | 11 hpf | total-11hpf | 24 hpf | total-24hpf |
| batch 1 | 44 | 47 |  |  | 19 | 24 |
| batch 2 | 35 | 64 |  |  | 58 | 61 |
|  | 79 | 111 | 0 | 0 | 77 | 85 |
|  |  |  |  |  |  |  |
| D141-Bp | 8 hpf | total-8hpf | 11 hpf | total-11hpf | 24 hpf | total-24hpf |
| batch 1 |  |  |  |  | 57 | 68 |
| batch 2 |  |  | 51 | 51 | 45 | 45 |
|  | 0 | 0 | 51 | 51 | 102 | 113 |
|  |  |  |  |  |  |  |
| D105△42-Bp | 8 hpf | total-8hpf | 11 hpf | total-11hpf | 24 hpf | total-24hpf |
| batch 1 |  |  |  |  | 27 | 34 |
| batch 2 | 9 | 29 |  |  | 25 | 27 |
|  | 9 | 29 | 0 | 0 | 52 | 61 |
|  |  |  |  |  |  |  |
| D105△30-Bp | 8 hpf | total-8hpf | 11 hpf | total-11hpf | 24 hpf | total-24hpf |
| batch 1 |  |  |  |  | 2 | 18 |
| batch 2 | 3 | 30 |  |  |  |  |
| batch 3 | 0 | 33 |  |  |  |  |
| batch 4 |  |  |  |  | 0 | 53 |
|  | 3 | 63 | 0 | 0 | 2 | 71 |
|  |  |  |  |  |  |  |
| D69△30-Bp | 8 hpf | total-8hpf | 11 hpf | total-11hpf | 24 hpf | total-24hpf |
| batch 1 | 37 | 71 |  |  | 5 | 68 |
| batch 2 |  |  |  |  | 2 | 57 |
|  | 37 | 71 | 0 | 0 | 7 | 125 |
|  |  |  |  |  |  |  |
| D184△74-Bp | 8 hpf | total-8hpf | 11 hpf | total-11hpf | 24 hpf | total-24hpf |
| batch 1 |  |  |  |  | 7 | 18 |
| batch 2 | 0 | 54 |  |  | 13 | 32 |
| batch 3 |  |  |  |  | 39 | 65 |
|  | 0 | 54 | 0 | 0 | 59 | 115 |
|  |  |  |  |  |  |  |
| A△342-Bp | 8 hpf | total-8hpf | 11 hpf | total-11hpf | 24 hpf | total-24hpf |
| batch 1 |  |  |  |  | 25 | 79 |
| batch 2 |  |  |  |  | 17 | 50 |
| batch 3 |  |  |  |  | 14 | 58 |
| batch 5 |  |  |  |  | 10 | 62 |
| batch 6 |  |  |  |  | 22 | 22 |
|  | 0 | 0 | 0 | 0 | 56 | 187 |
|  |  |  |  |  |  |  |
| A462-Bp | 8 hpf | total-8hpf | 11 hpf | total-11hpf | 24 hpf | total-24hpf |
| batch 1 |  |  |  |  | 18 | 25 |
| batch 2 |  |  |  |  | 16 | 34 |
| batch 3 | 0 | 61 | 0 | 59 | 44 | 56 |
|  | 0 | 61 | 0 | 59 | 78 | 115 |
|  |  |  |  |  |  |  |
| A433-Bp | 8 hpf | total-8hpf | 11 hpf | total-11hpf | 24 hpf | total-24hpf |
| batch 1 |  |  |  |  | 11 | 13 |
| batch 3 | 0 | 31 | 0 | 31 | 22 | 25 |
| batch 4 |  |  |  |  | 10 | 58 |
|  | 0 | 31 | 0 | 31 | 33 | 38 |
|  |  |  |  |  |  |  |
| A415-Bp | 8 hpf | total-8hpf | 11 hpf | total-11hpf | 24 hpf | total-24hpf |
| batch 1 |  |  |  |  | 12 | 24 |
| batch 2 |  |  |  |  | 3 | 35 |
| batch 3 | 0 | 40 | 0 | 39 | 11 | 26 |
|  | 0 | 40 | 0 | 39 | 23 | 50 |
|  |  |  |  |  |  |  |
| A392-Bp | 8 hpf | total-8hpf | 11 hpf | total-11hpf | 24 hpf | total-24hpf |
| batch 1 |  |  |  |  | 31 | 39 |
| batch 2 |  |  |  |  | 8 | 54 |
| batch 3 |  |  |  |  | 31 | 43 |
| batch 4 |  |  |  |  | 31 | 60 |
| batch 5 |  |  |  |  | 4 | 17 |
| batch 6 |  |  |  |  | 2 | 31 |
| batch 7 |  |  | 0 | 39 | 4 | 35 |
| batch 8 |  |  |  |  | 0 | 61 |
| batch 9 | 0 | 40 |  |  | 9 | 38 |
|  | 0 | 40 | 0 | 39 | 120 | 378 |
|  |  |  |  |  |  |  |
| A315-Bp | 8 hpf | total-8hpf | 11 hpf | total-11hpf | 24 hpf | total-24hpf |
| batch 1 |  |  |  |  | 3 | 31 |
| batch 2 |  |  |  |  | 0 | 36 |
| batch 3 | 0 | 38 | 0 | 38 | 1 | 37 |
|  | 0 | 38 | 0 | 38 | 4 | 104 |
|  |  |  |  |  |  |  |
| A22(2)-Bp | 8 hpf | total-8hpf | 11 hpf | total-11hpf | 24 hpf | total-24hpf |
| batch 2 |  |  |  |  | 0 | 42 |
|  | 0 | 0 | 0 | 0 | 0 | 42 |
|  |  |  |  |  |  |  |
| A△372-Bp | 8 hpf | total-8hpf | 11 hpf | total-11hpf | 24 hpf | total-24hpf |
| batch 1 |  |  |  |  | 7 | 44 |
| batch 2 |  |  |  |  | 1 | 20 |
| batch 3 |  |  |  |  | 20 | 56 |
| batch 4 |  |  |  |  | 2 | 53 |
| batch 5 | 0 | 29 |  |  | 0 | 27 |
|  | 0 | 29 | 0 | 0 | 30 | 200 |
|  |  |  |  |  |  |  |
| A△298-Bp | 8 hpf | total-8hpf | 11 hpf | total-11hpf | 24 hpf | total-24hpf |
| batch 1 |  |  |  |  | 14 | 19 |
| batch 2 |  |  |  |  | 50 | 59 |
| batch 3 |  |  |  |  | 90 | 122 |
|  | 0 | 0 | 0 | 0 | 154 | 200 |
|  |  |  |  |  |  |  |
| A433△298-Bp | 8 hpf | total-8hpf | 11 hpf | total-11hpf | 24 hpf | total-24hpf |
| batch 1 |  |  |  |  | 31 | 57 |
| batch 2 |  |  |  |  | 29 | 52 |
|  | 0 | 0 | 0 | 0 | 60 | 109 |
|  |  |  |  |  |  |  |
| A392△298-Bp | 8 hpf | total-8hpf | 11 hpf | total-11hpf | 24 hpf | total-24hpf |
| batch 1 |  |  |  |  | 14 | 45 |
| batch 3 |  |  |  |  | 5 | 35 |
| batch 4 |  |  |  |  | 11 | 27 |
|  | 0 | 0 | 0 | 0 | 30 | 107 |
|  |  |  |  |  |  |  |
| A462△298-Bp-M | 8 hpf | total-8hpf | 11 hpf | total-11hpf | 24 hpf | total-24hpf |
| batch 1 |  |  |  |  | 25 | 39 |
| batch 2 |  |  |  |  | 39 | 67 |
|  | 0 | 0 | 0 | 0 | 64 | 106 |
|  |  |  |  |  |  |  |
| A462△298-Bp | 8 hpf | total-8hpf | 11 hpf | total-11hpf | 24 hpf | total-24hpf |
| batch 1 |  |  |  |  | 37 | 46 |
| batch 2 |  |  |  |  | 39 | 58 |
|  | 0 | 0 | 0 | 0 | 76 | 104 |
