# Supplementary figures and images for: Complexity of cis-regulatory organization of six3a during forebrain and eye development in zebrafish
Source: BMC Dev Biol. 2010 Mar 26;10:35. doi: 10.1186/1471-213X-10-35 (PMC2858731; doi:10.1186/1471-213X-10-35)

## Additional file 6-Additional images for 3087-Bp and 1060-Bp

**3087-Bp:**


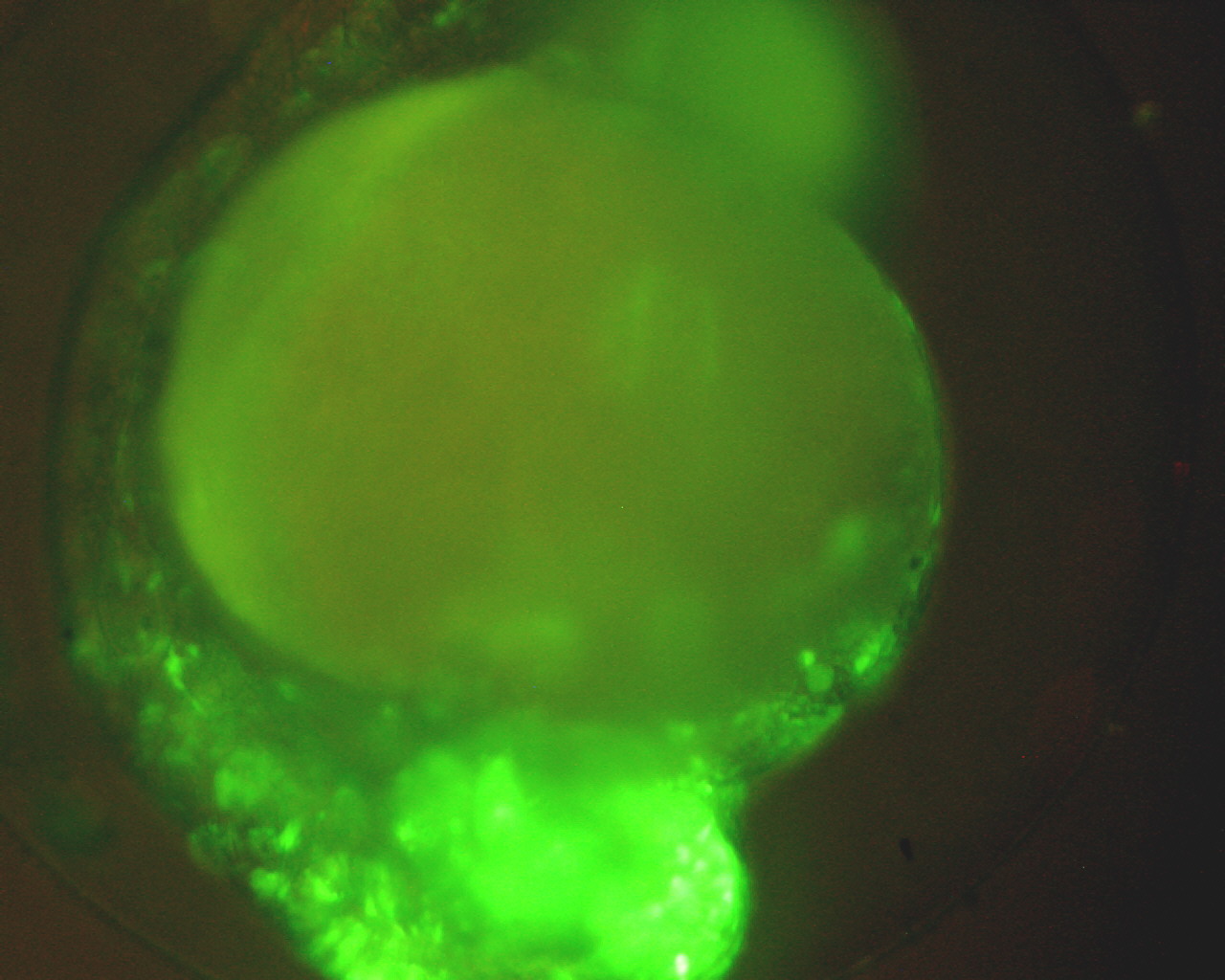

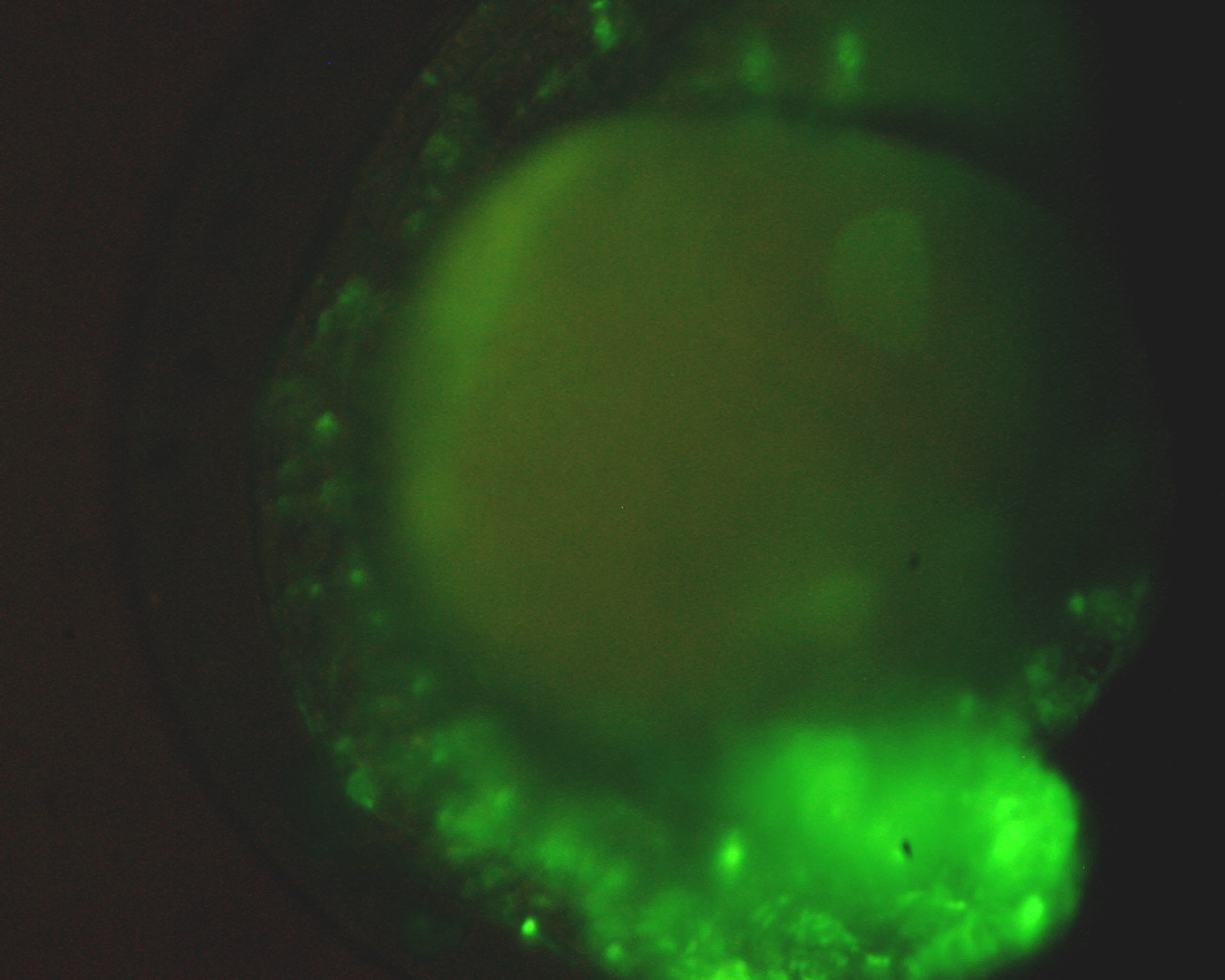

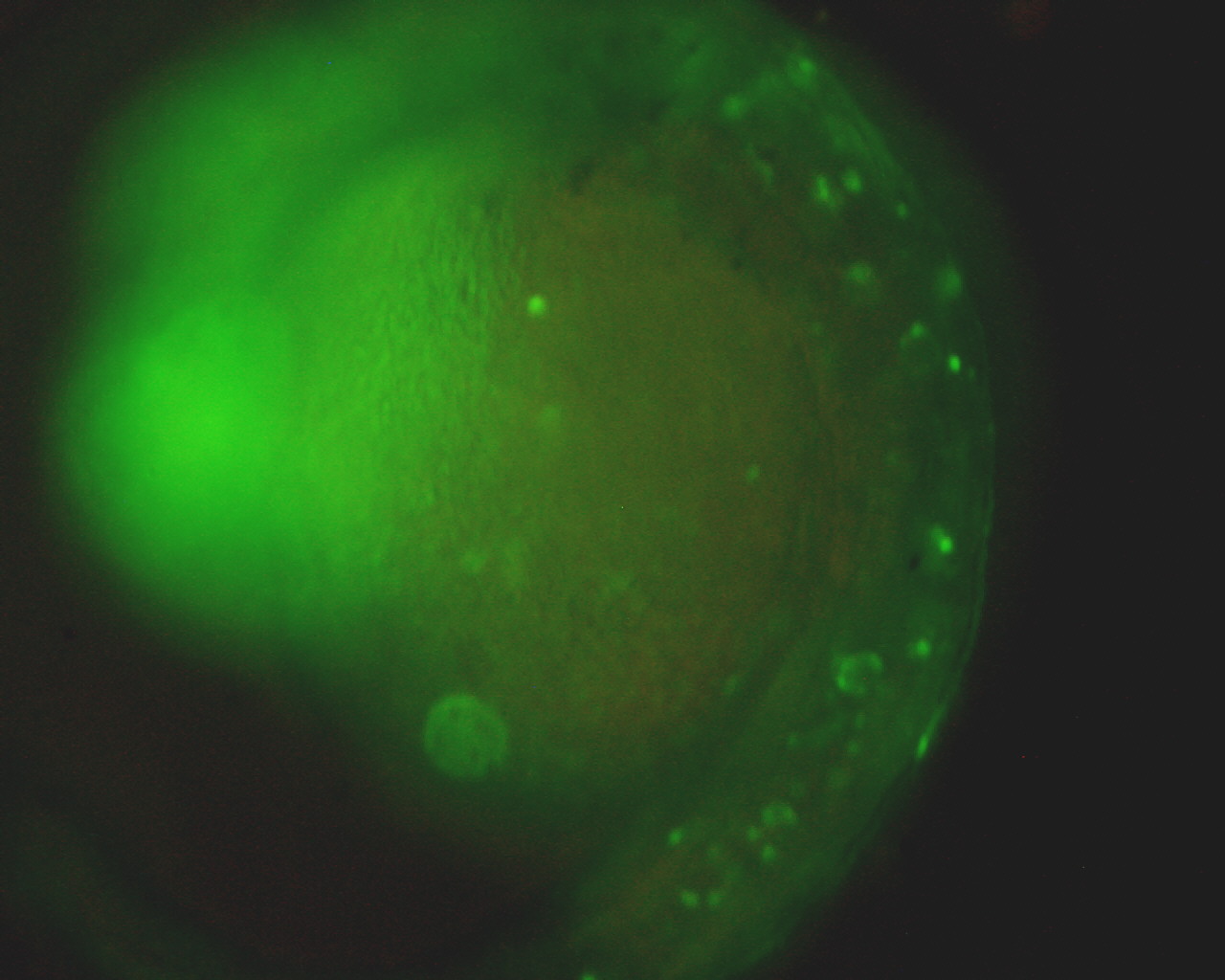

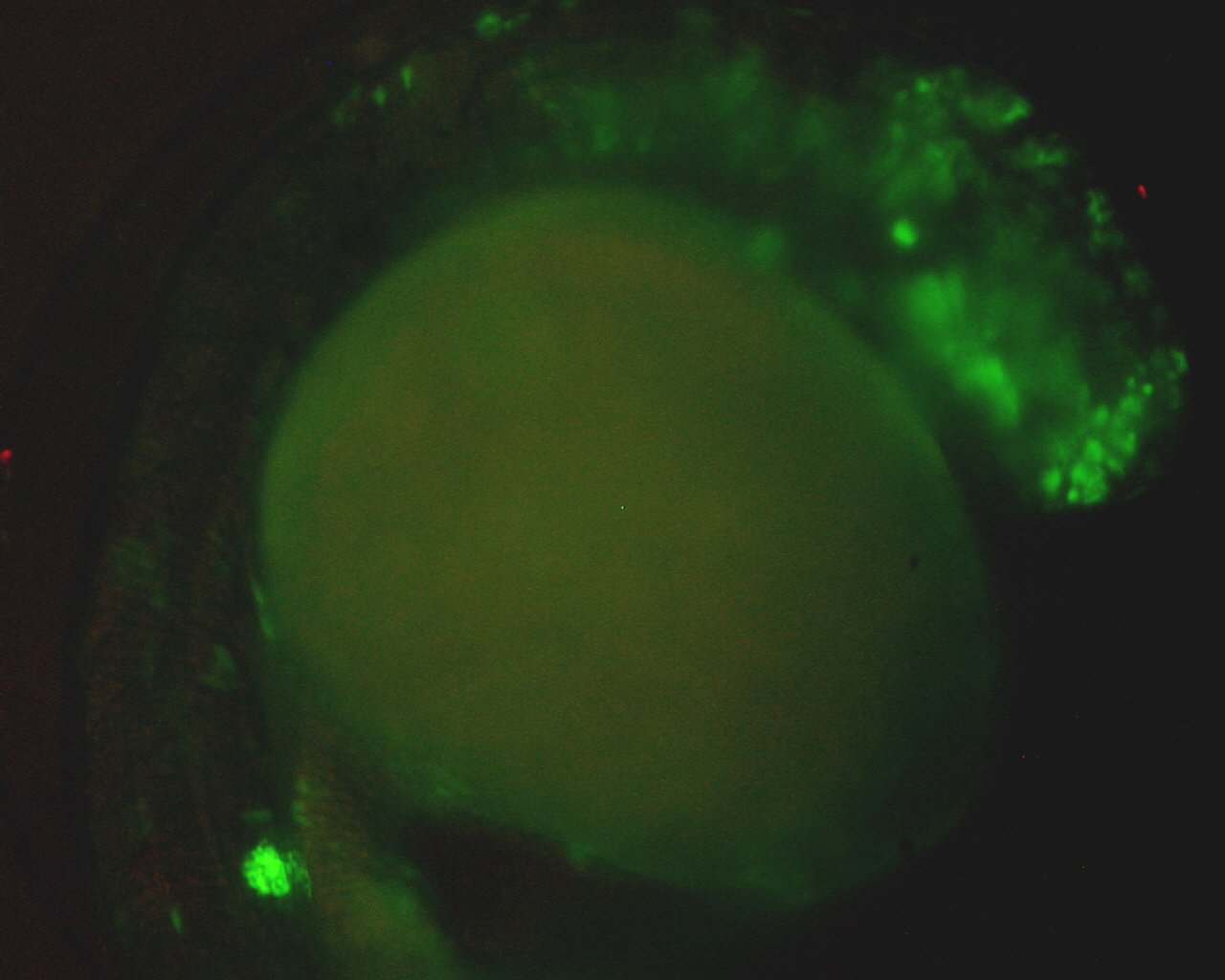

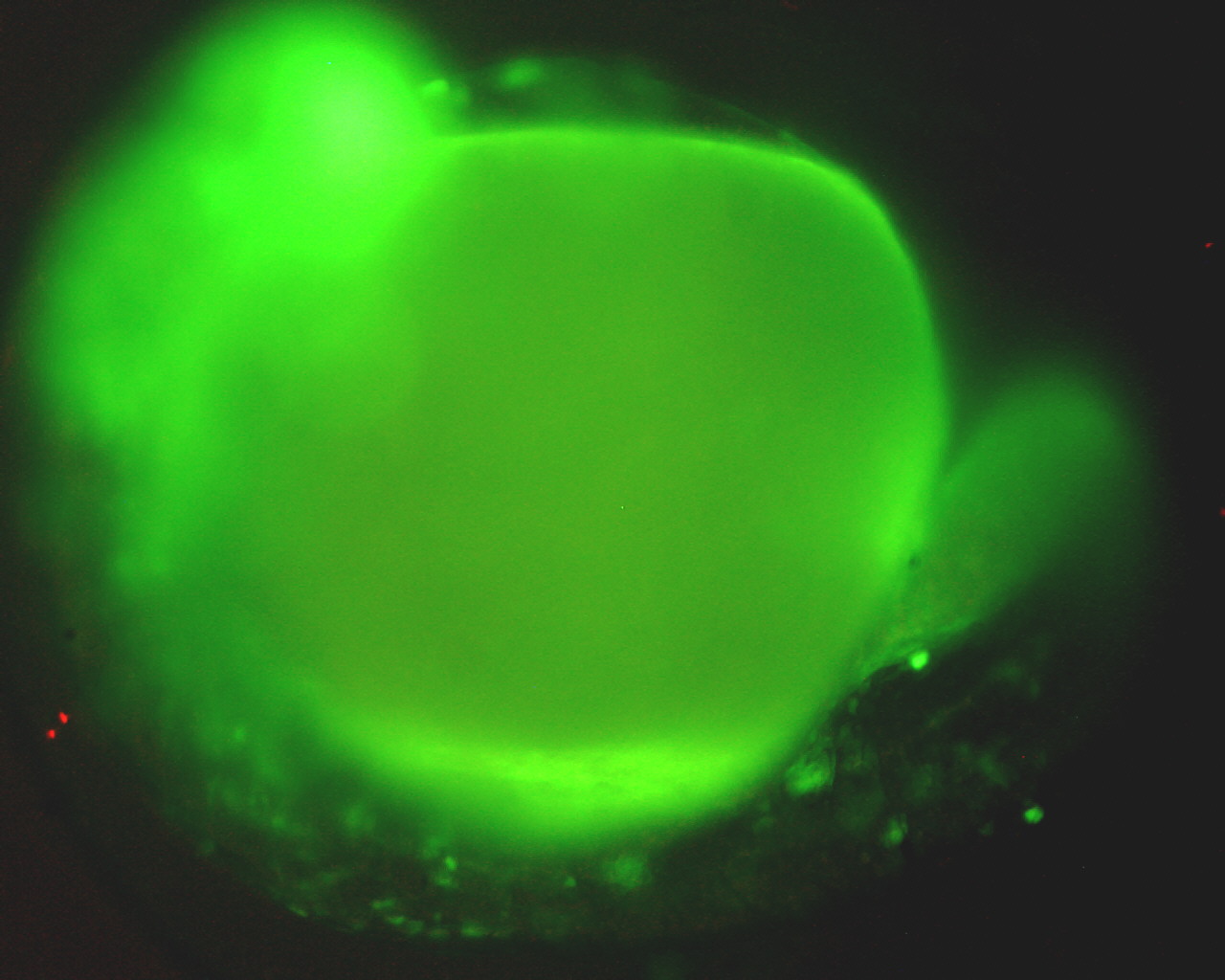

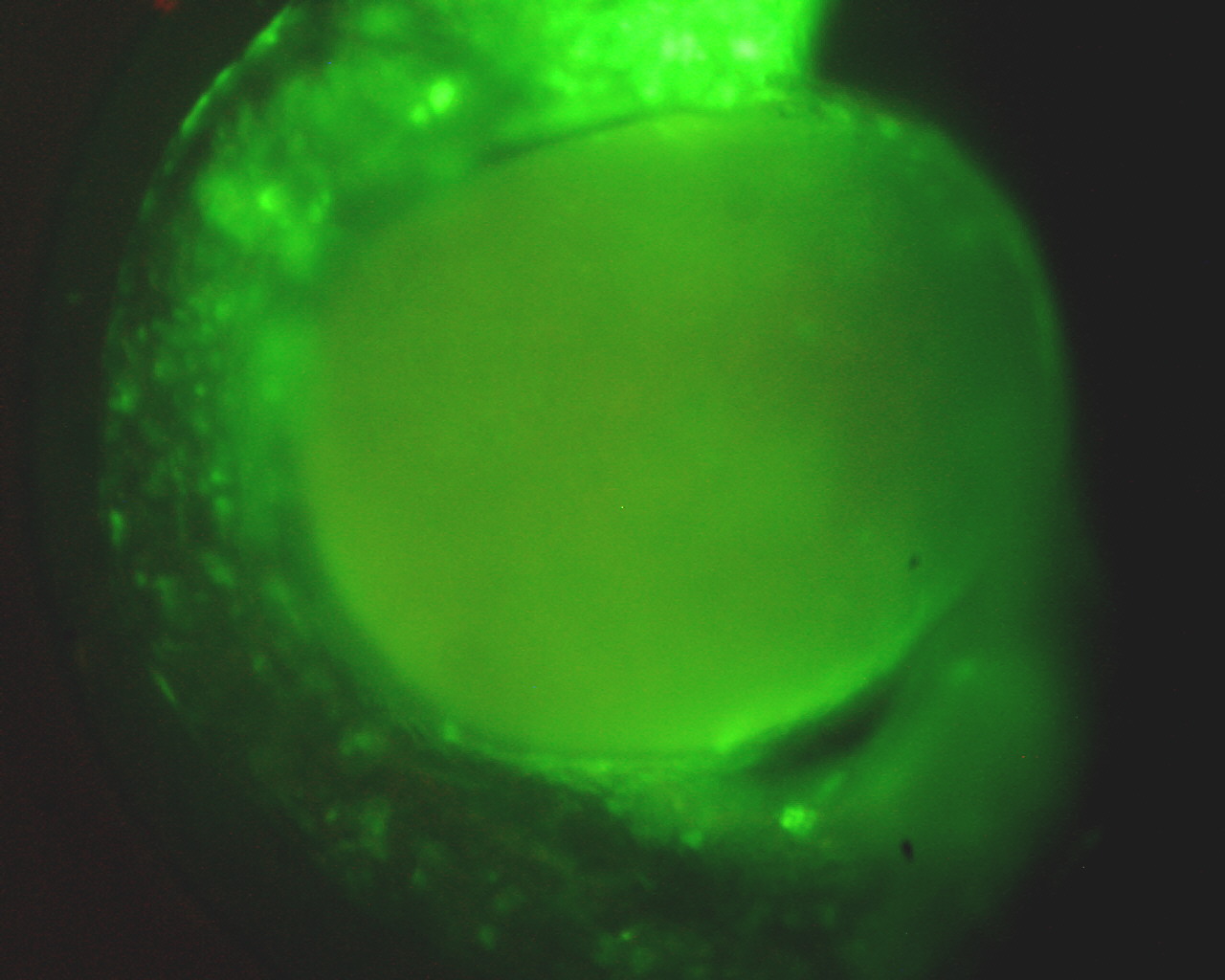


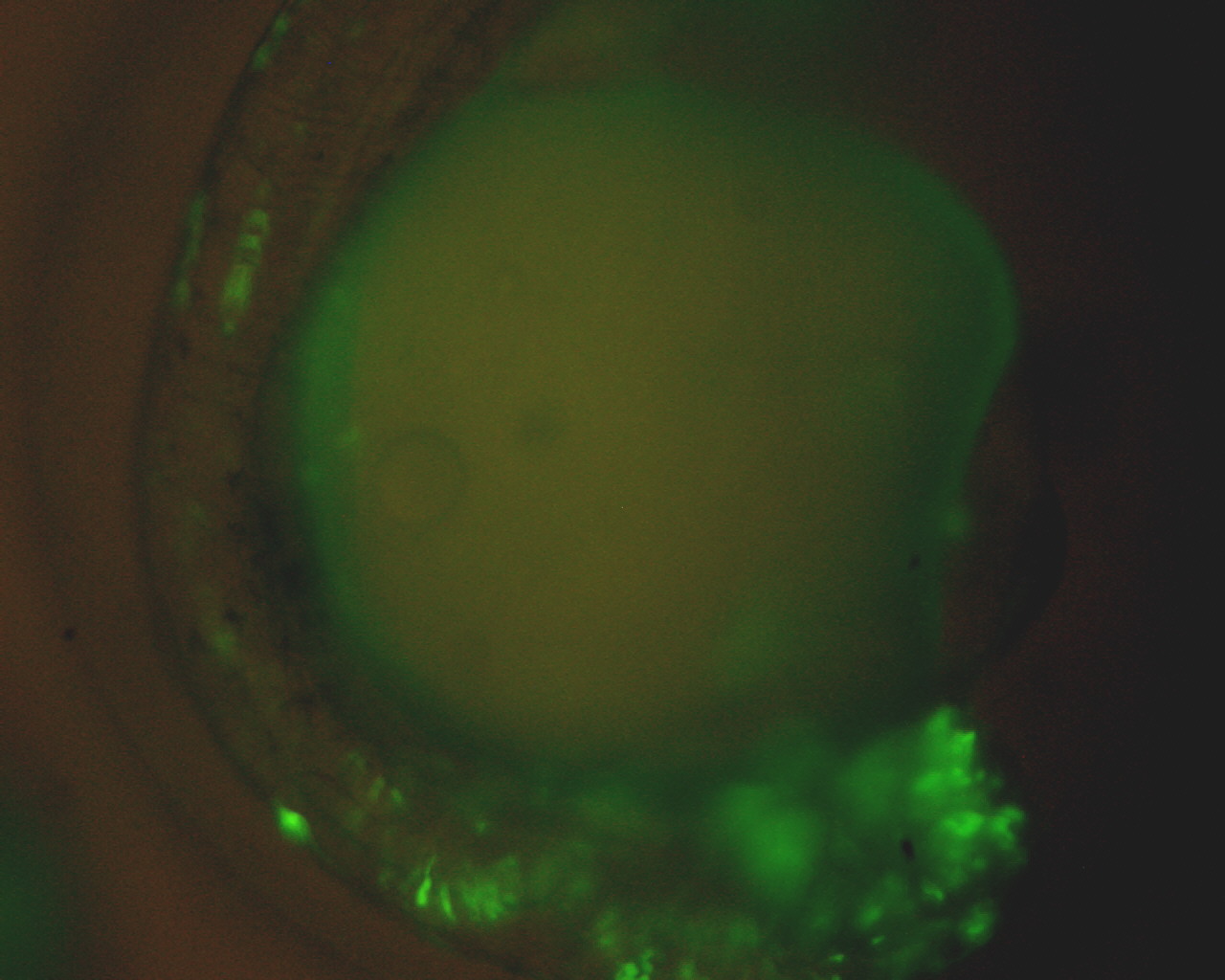


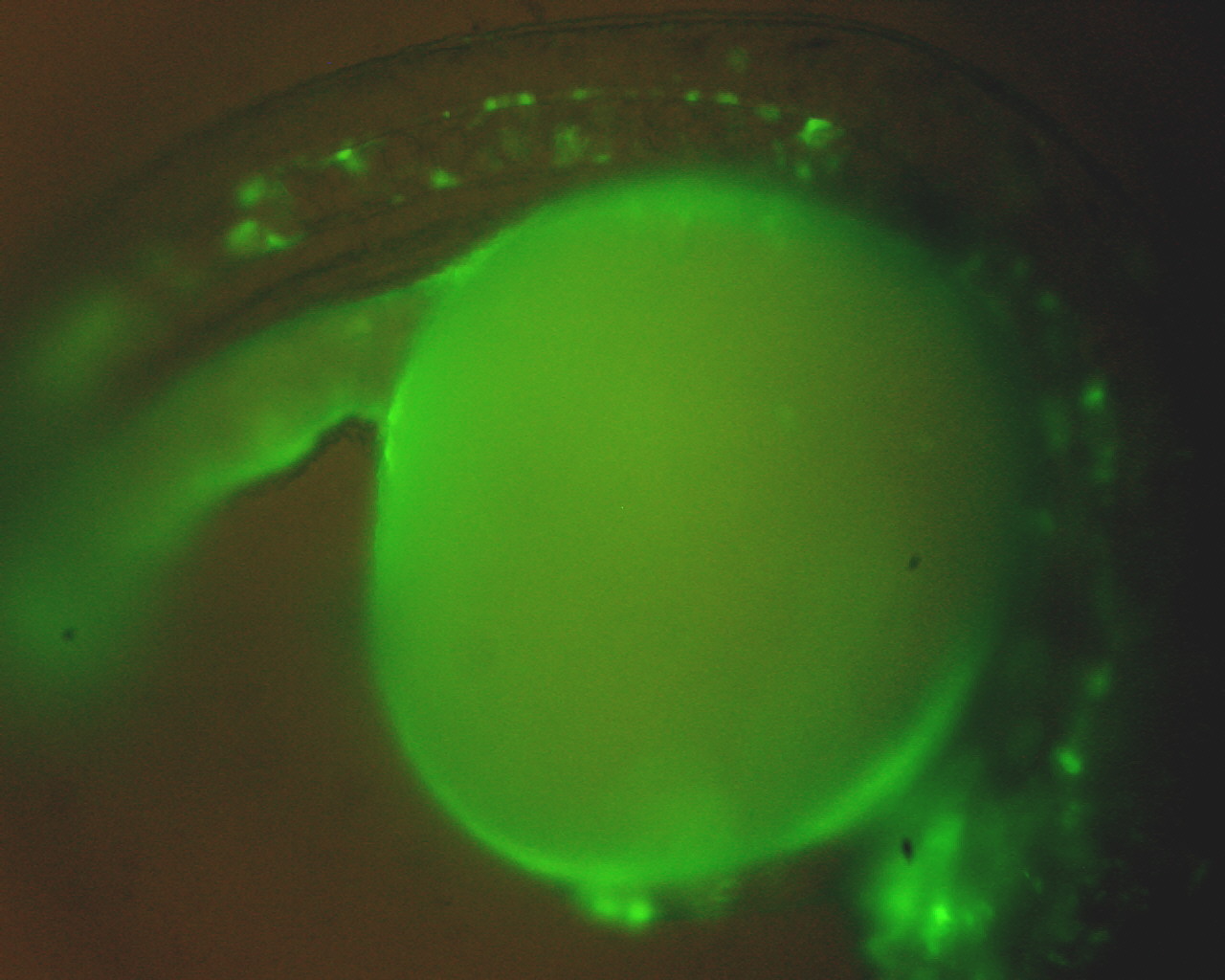

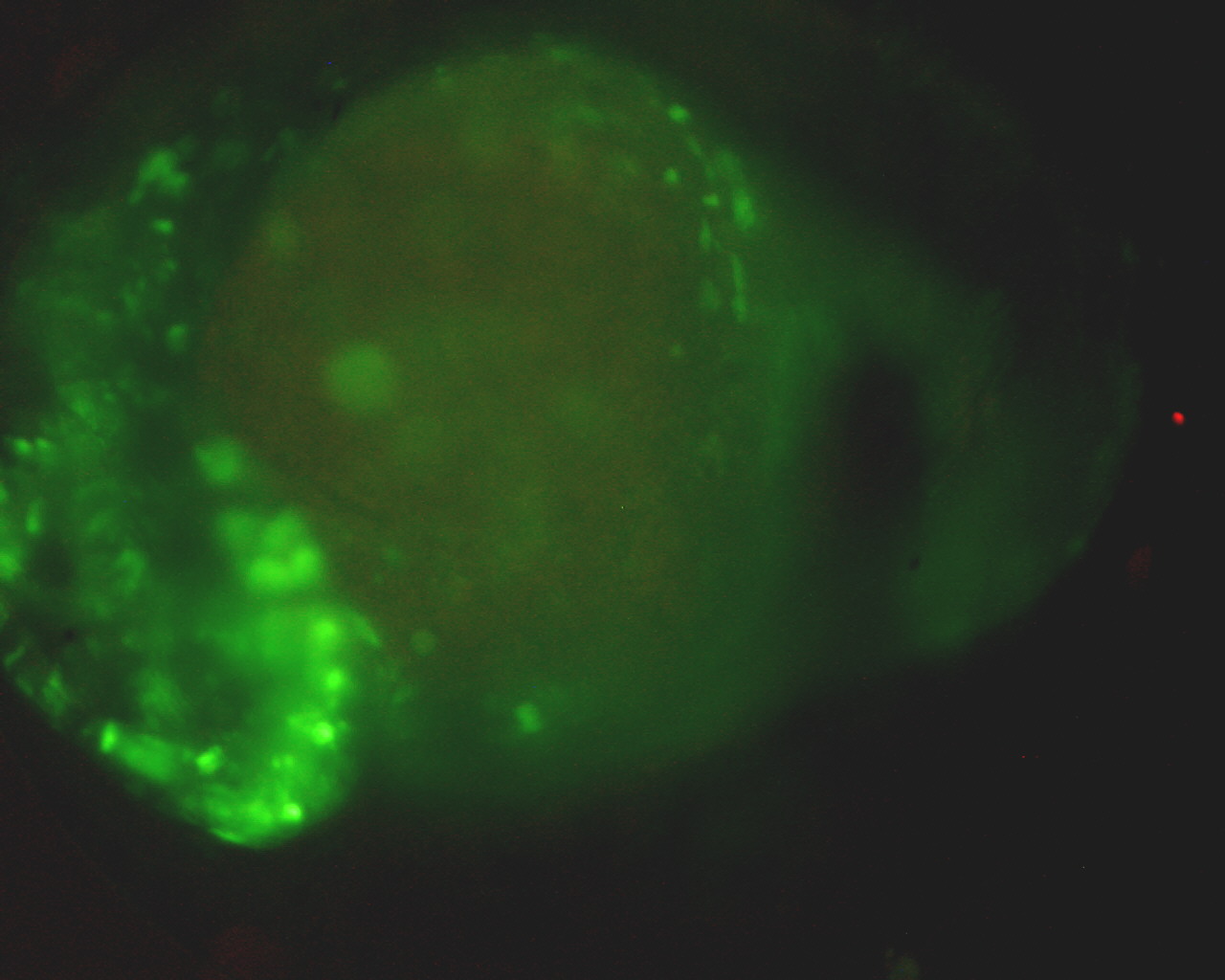

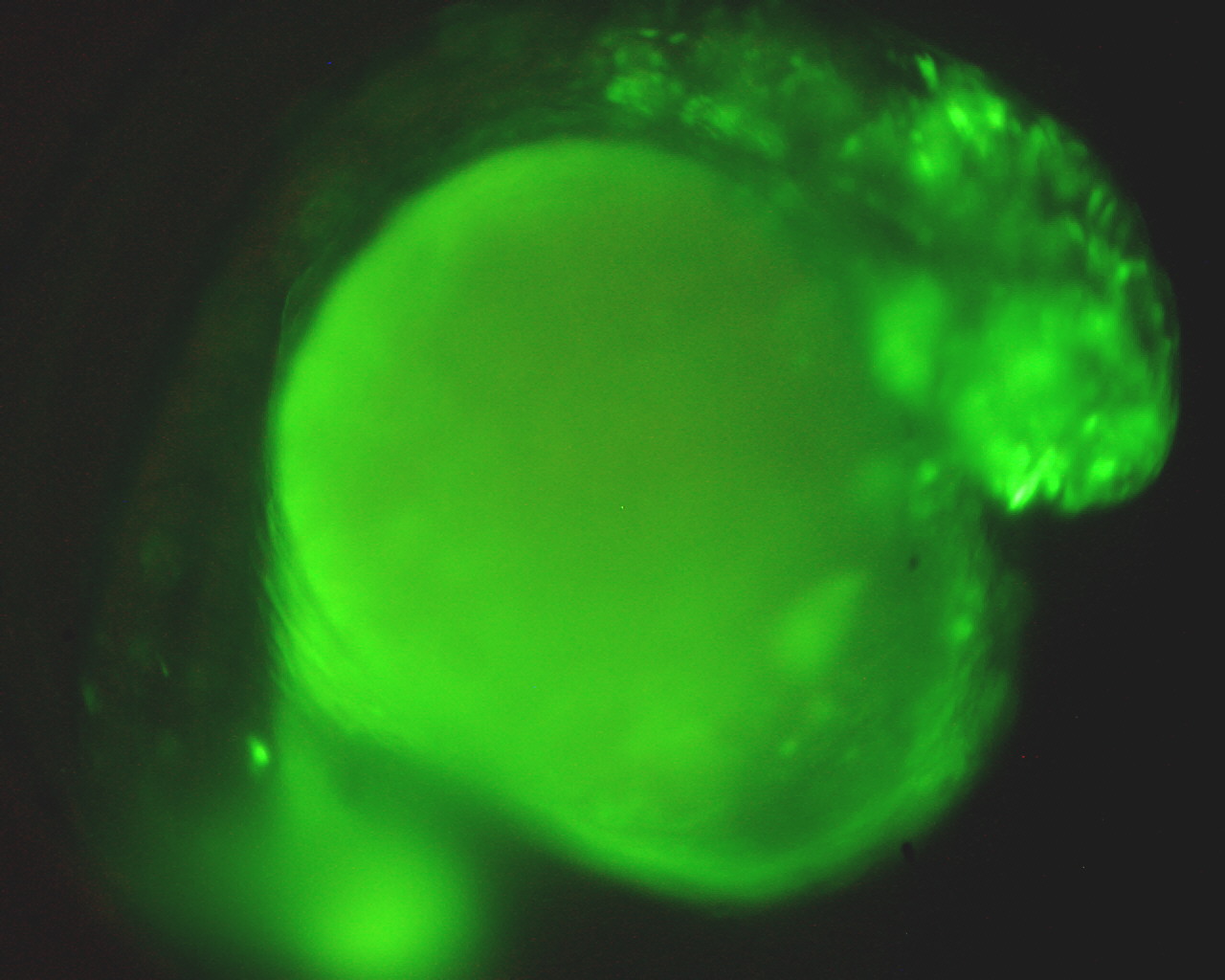

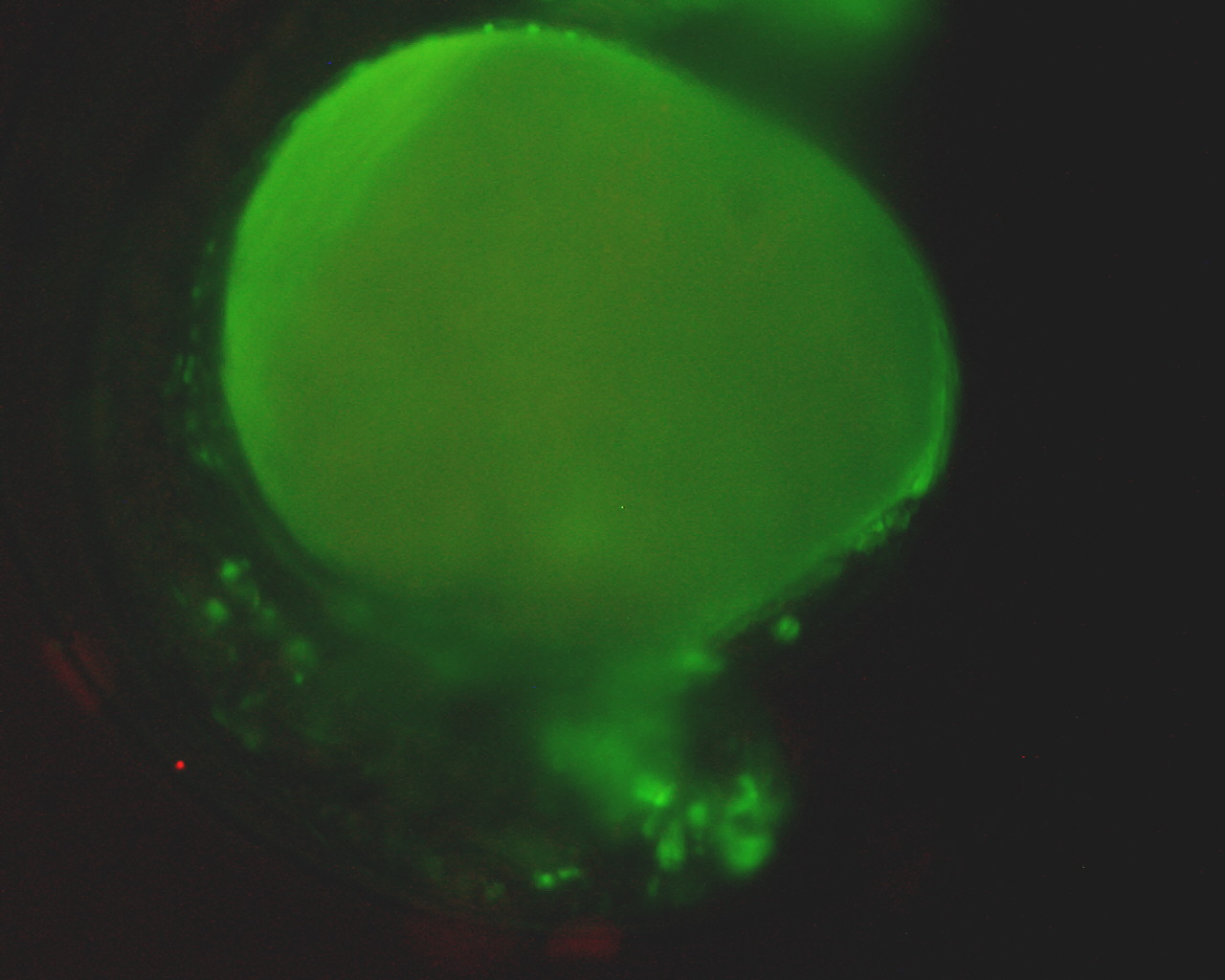

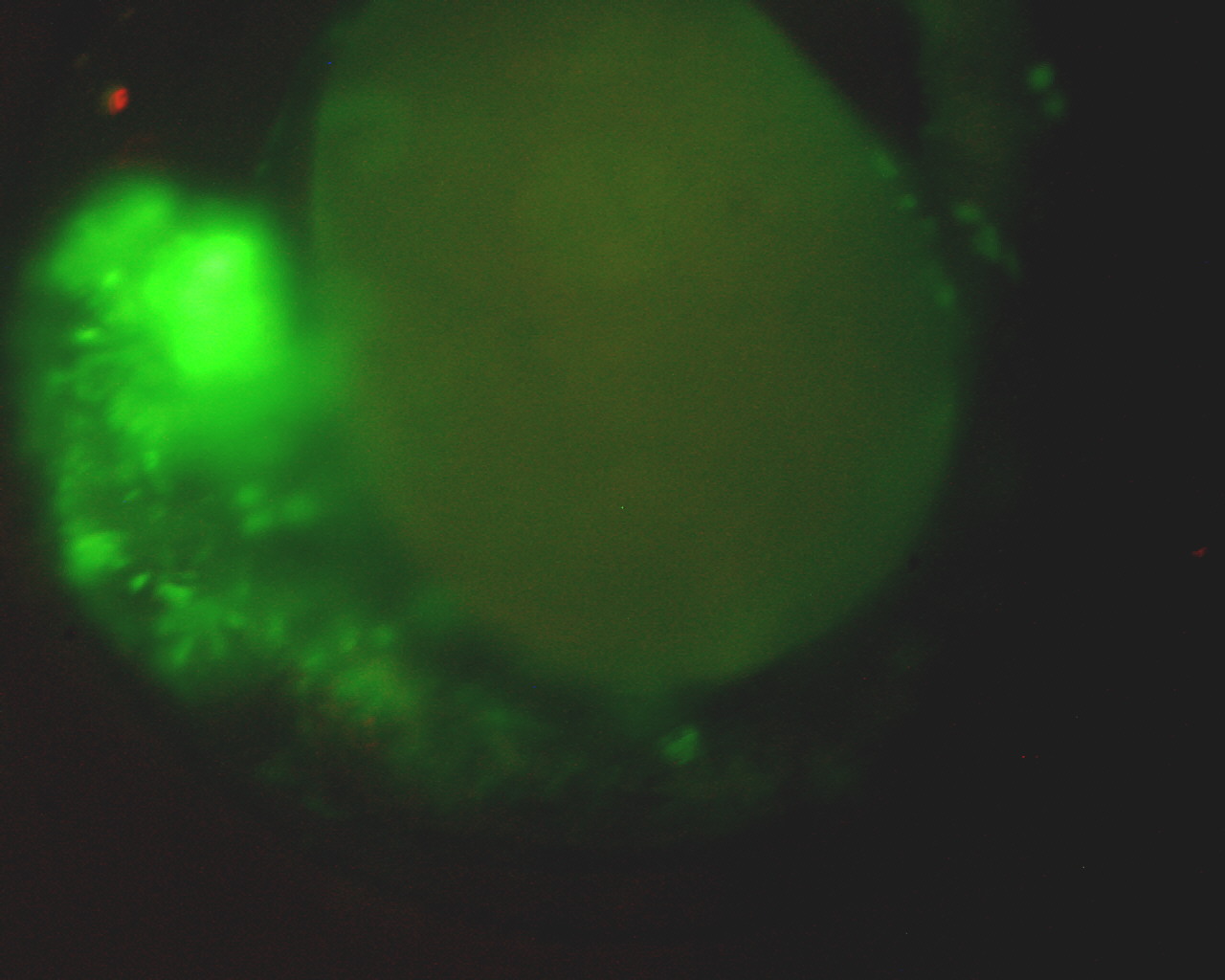

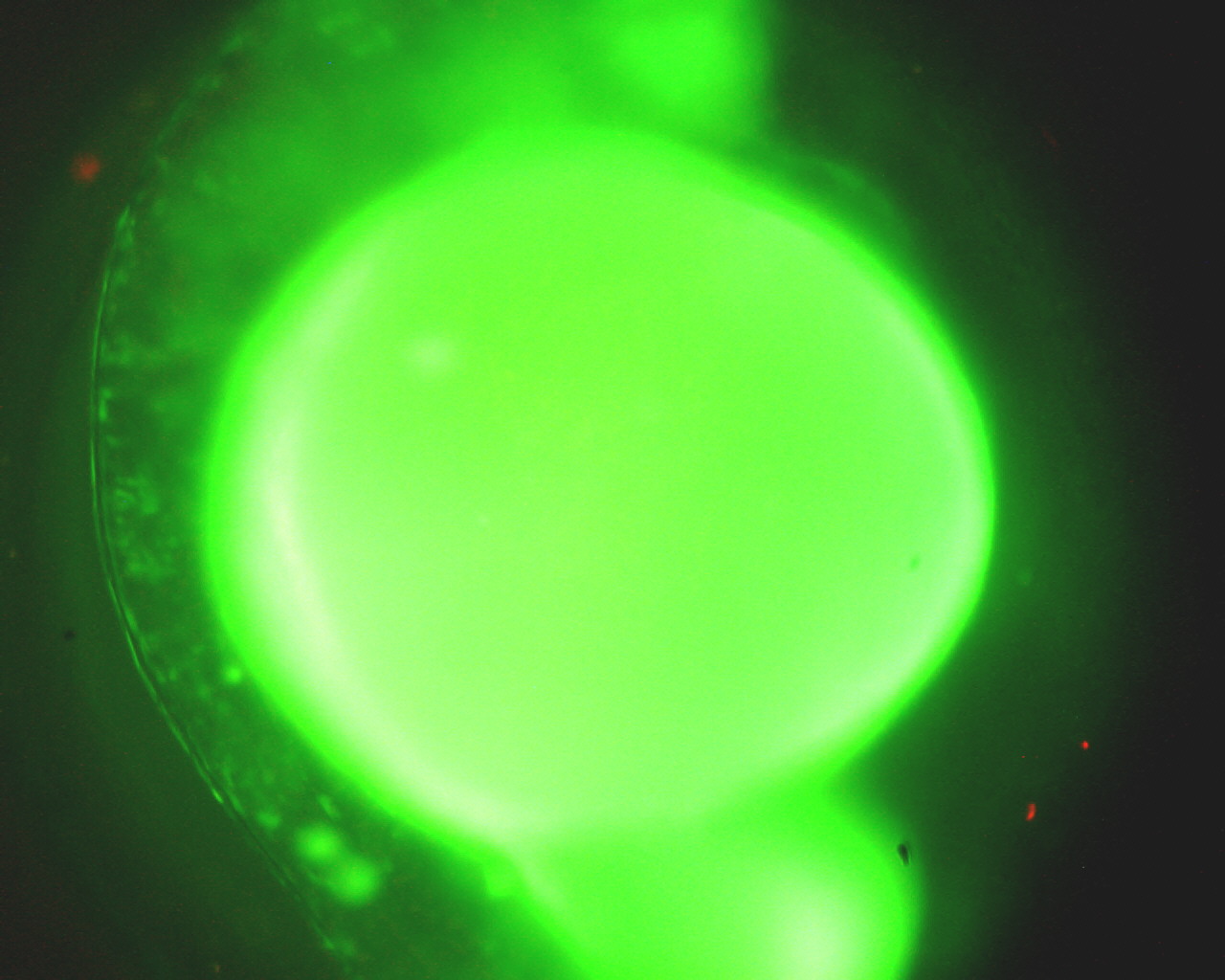

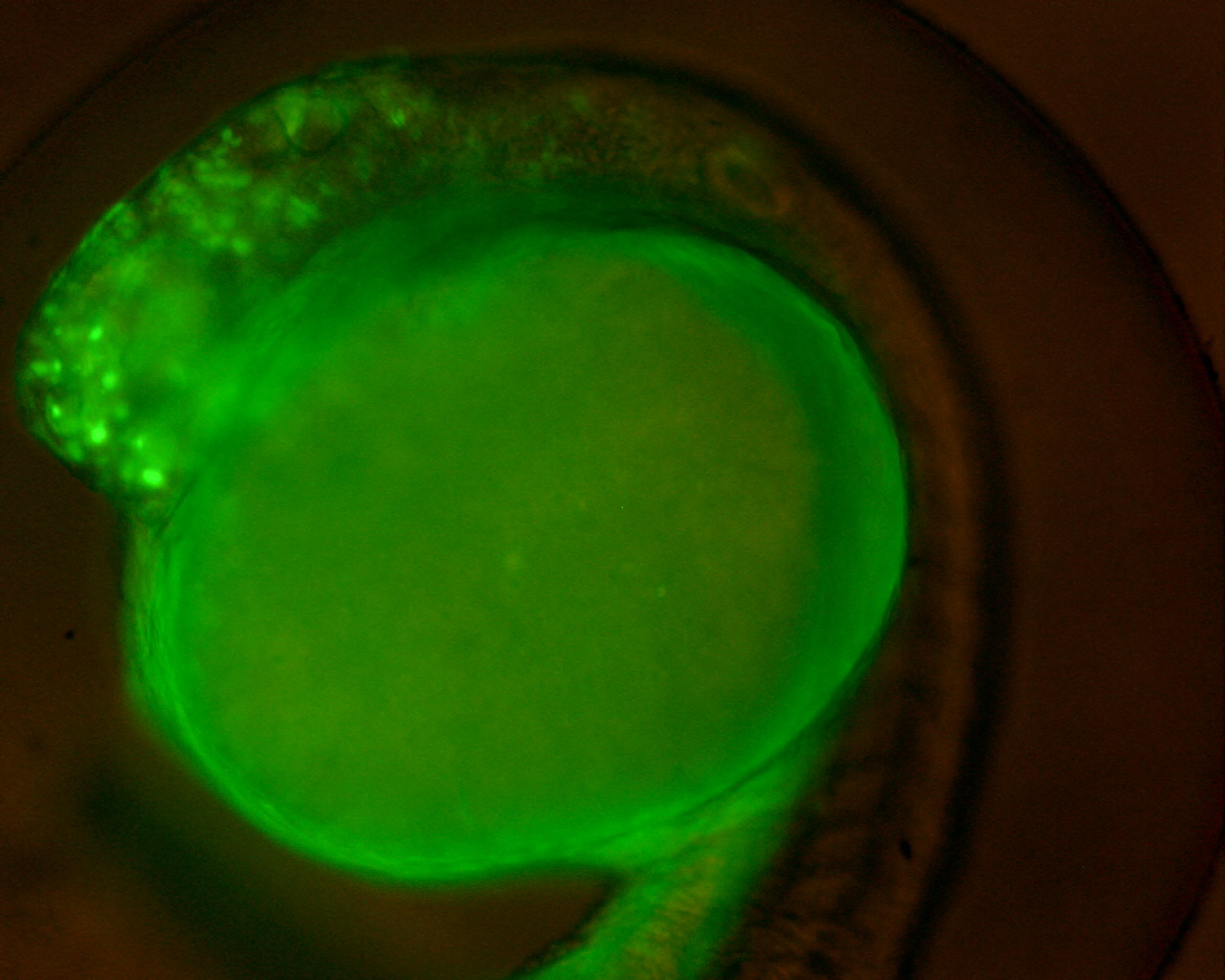


## 1060-Bp


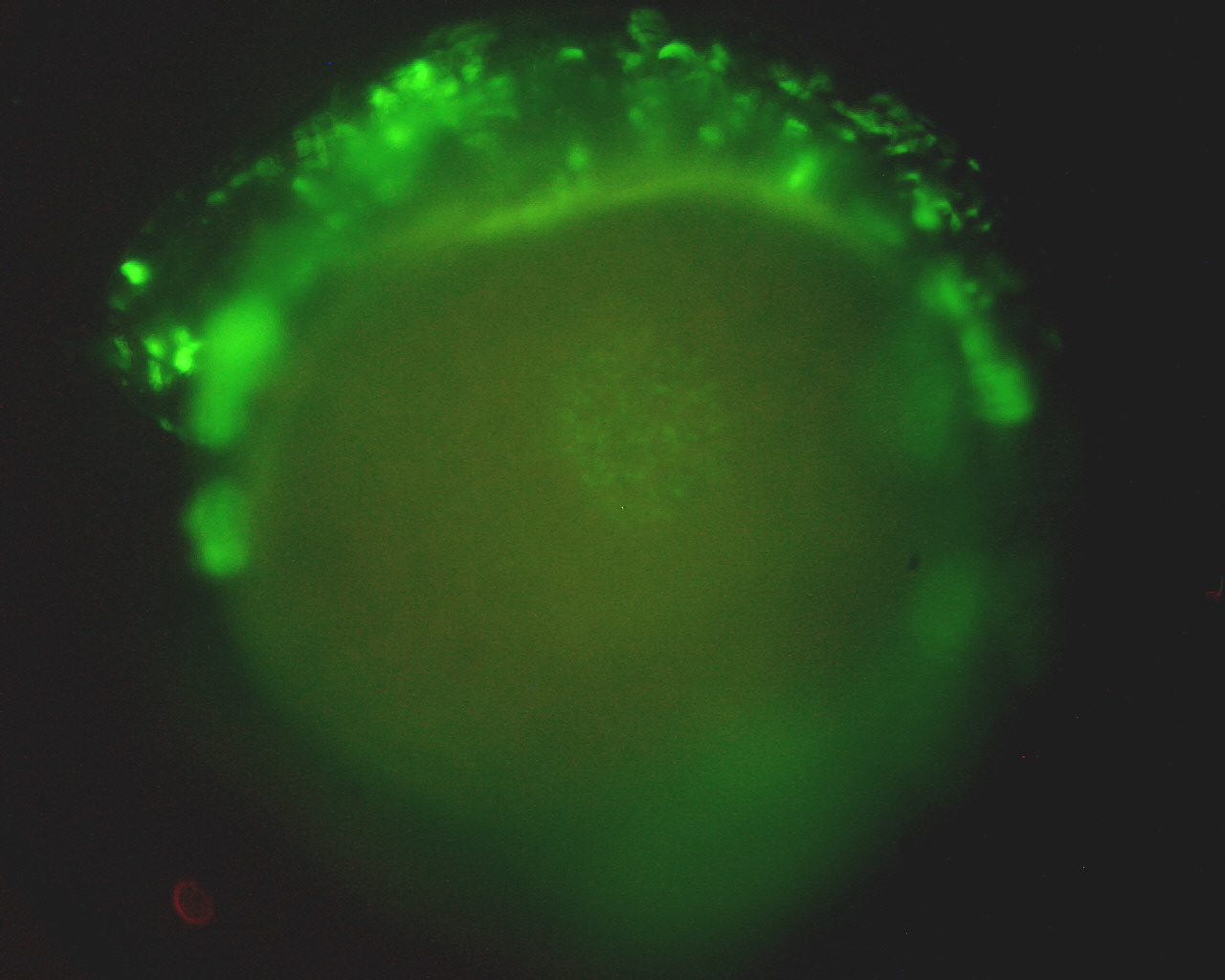

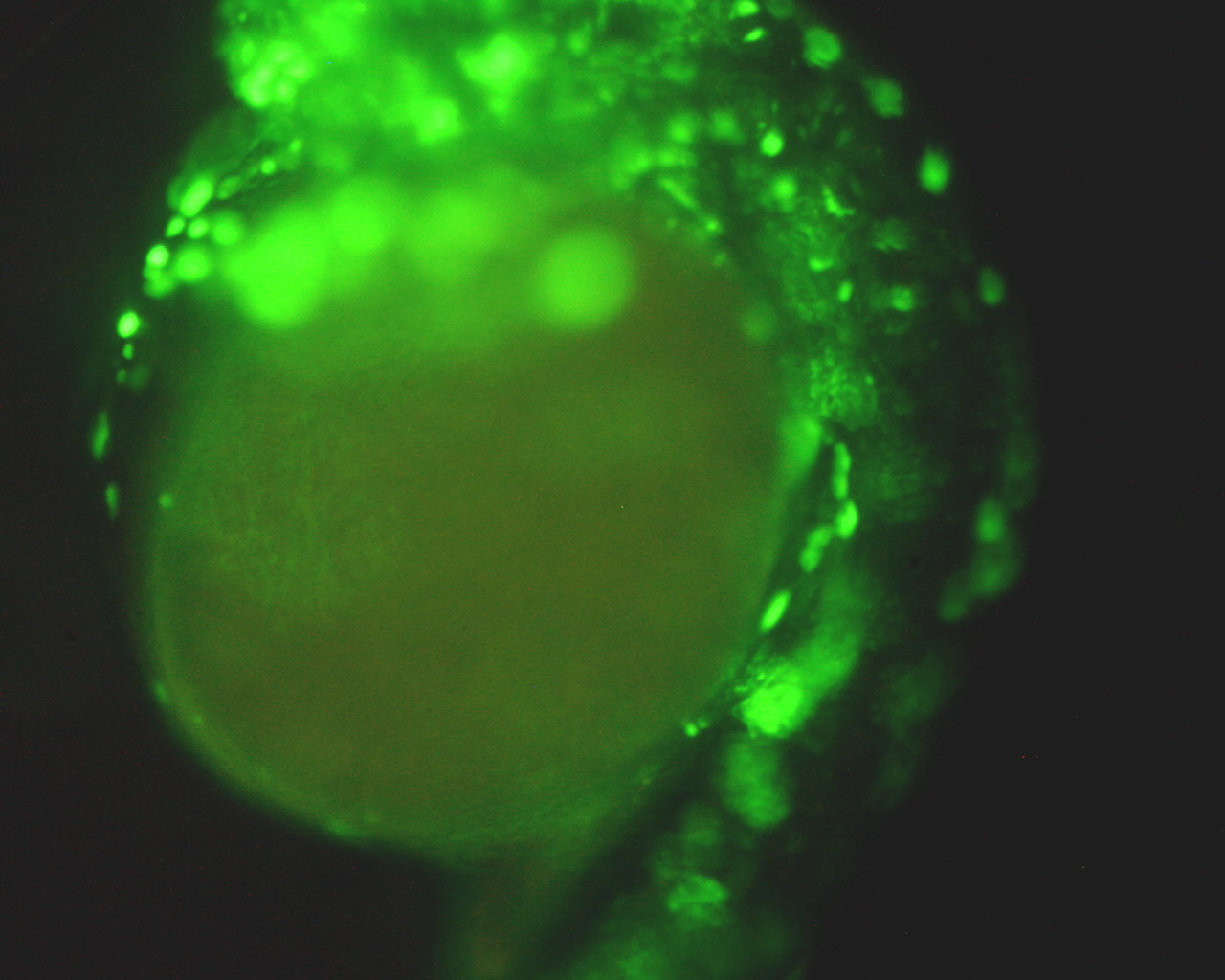

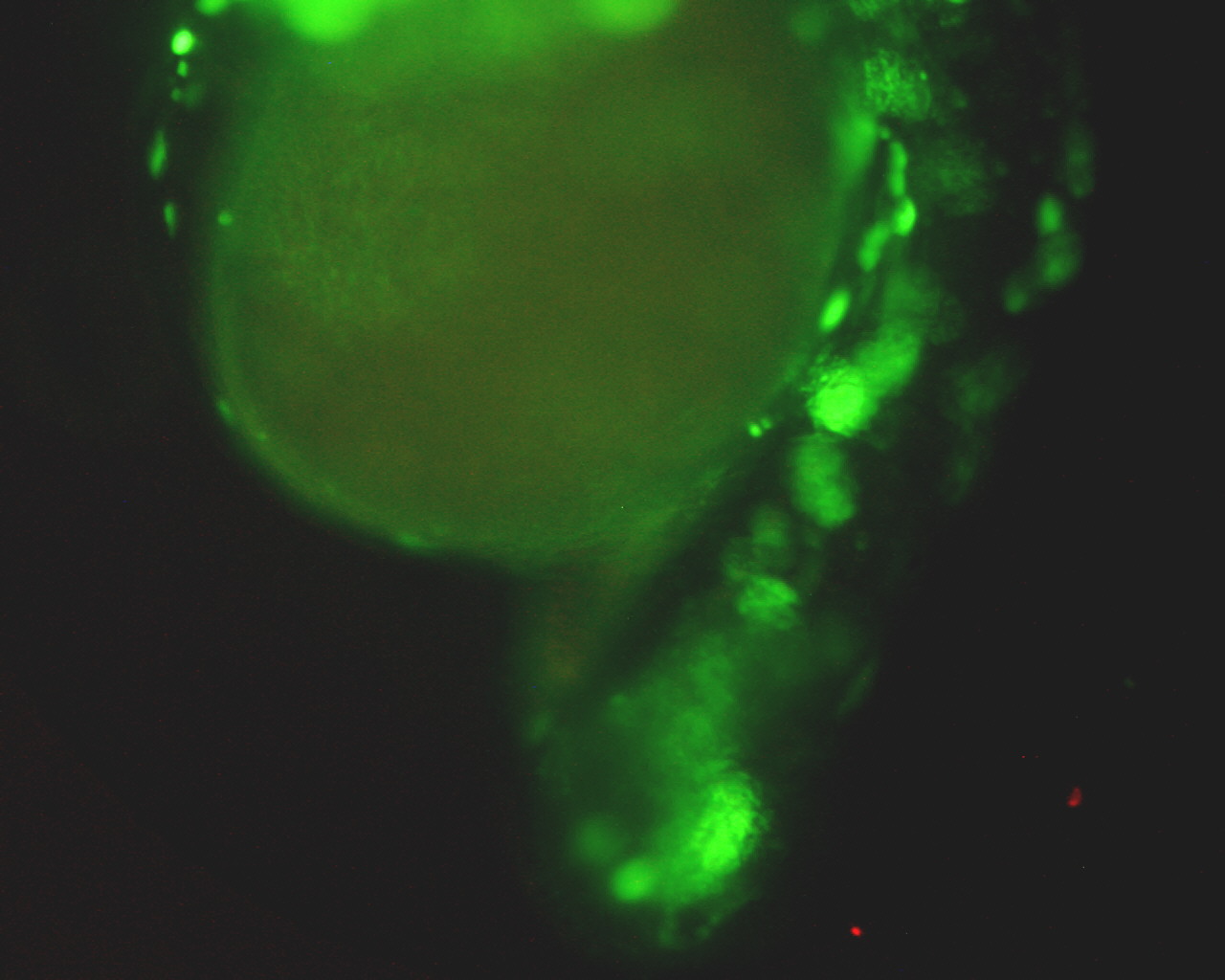

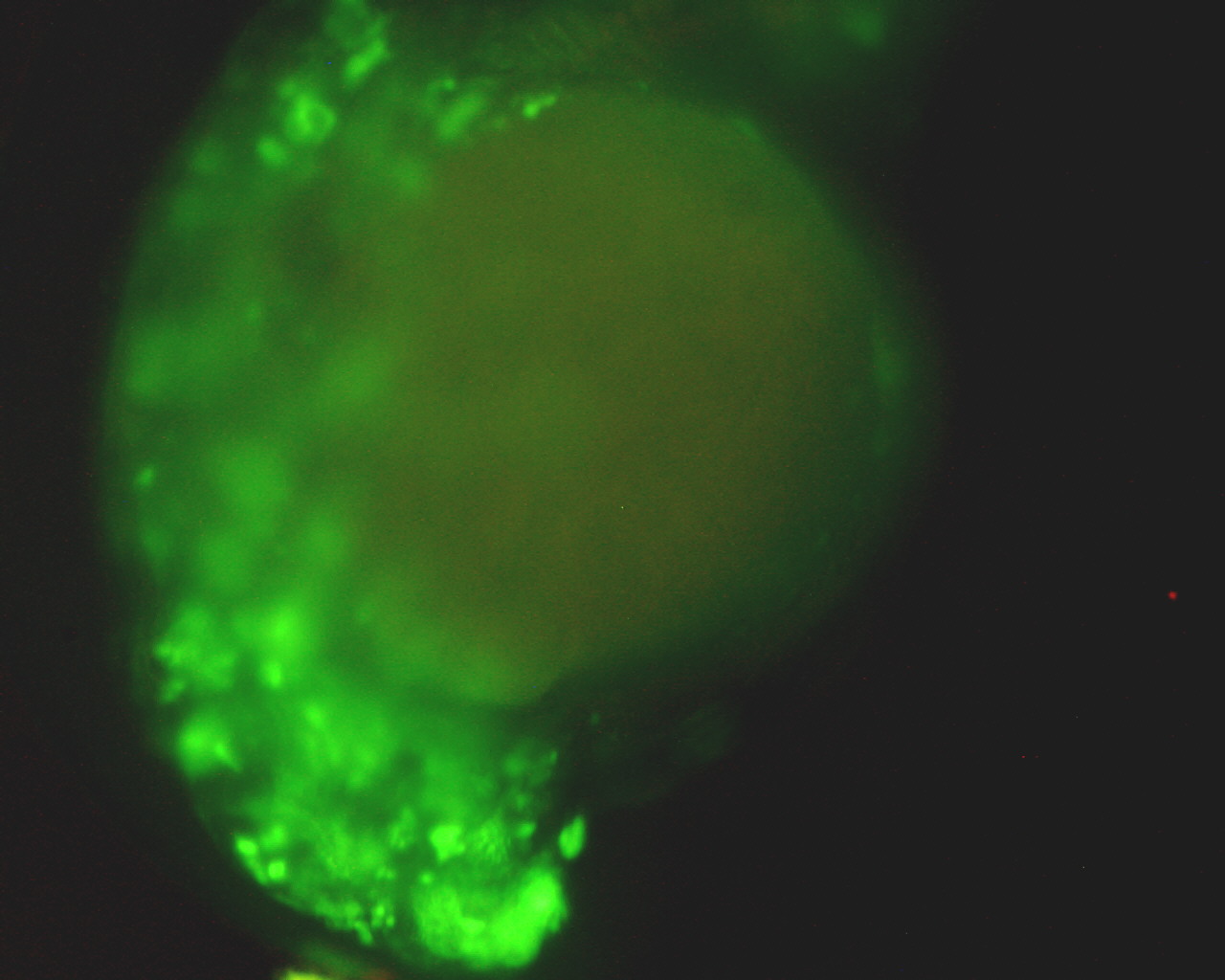

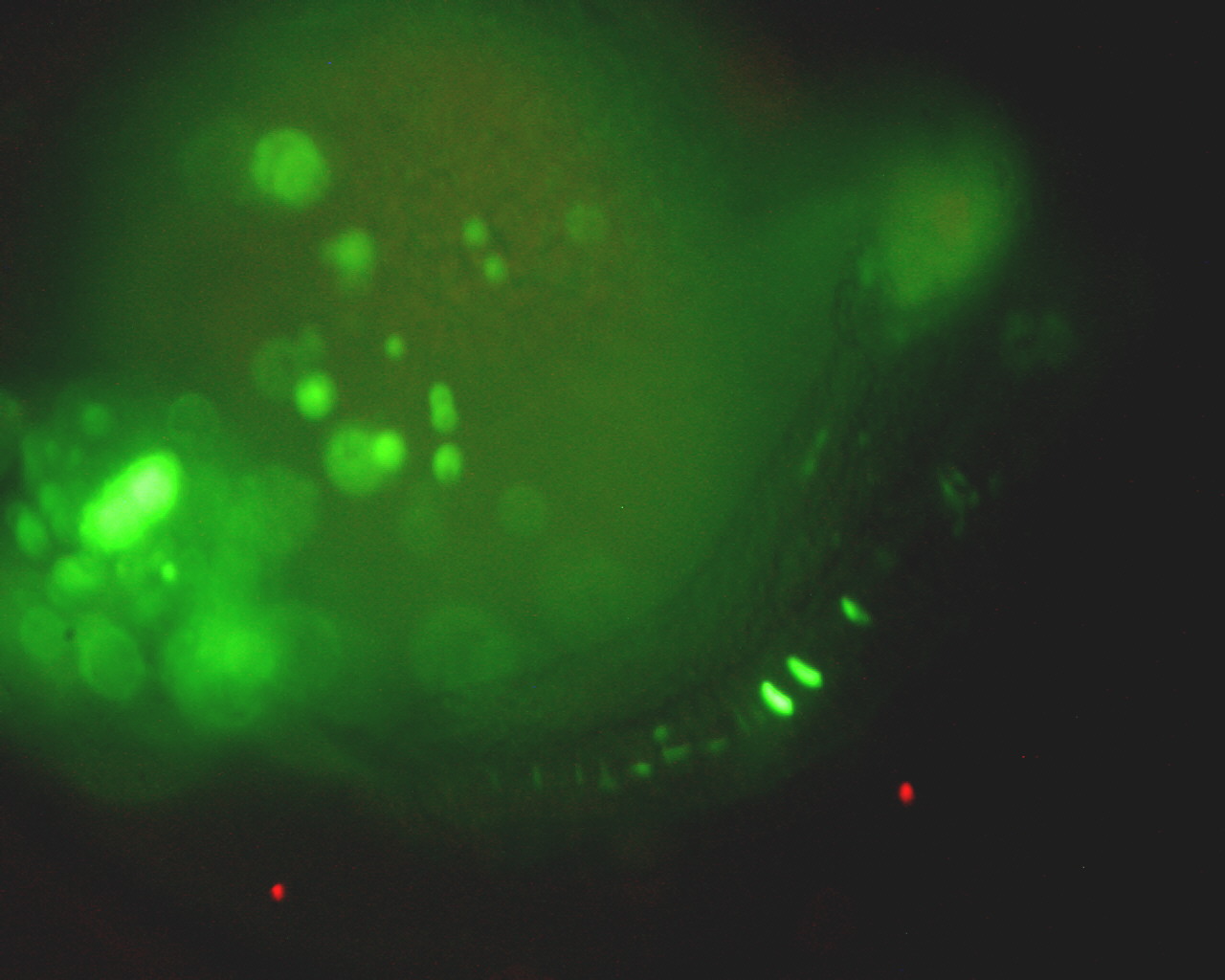

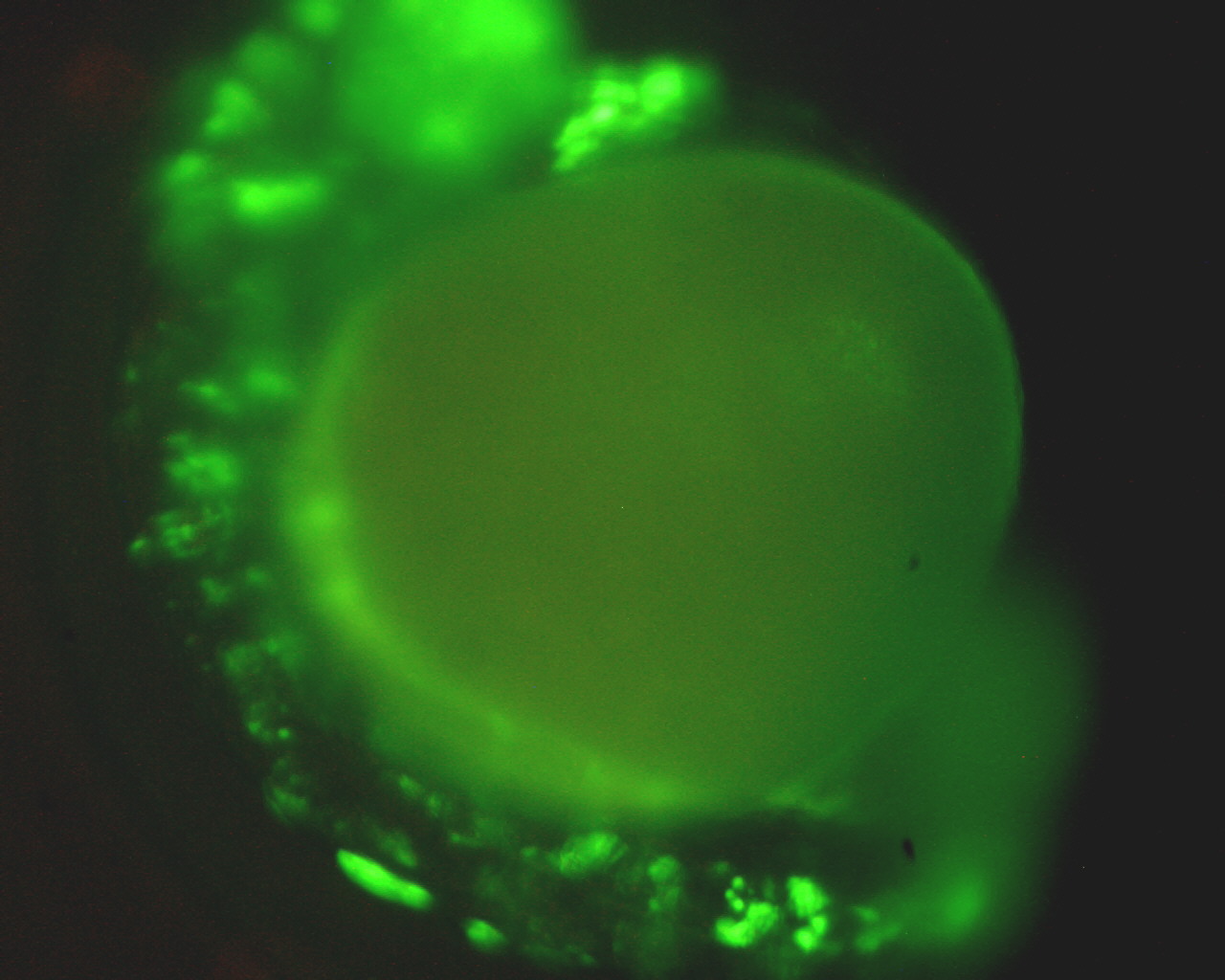

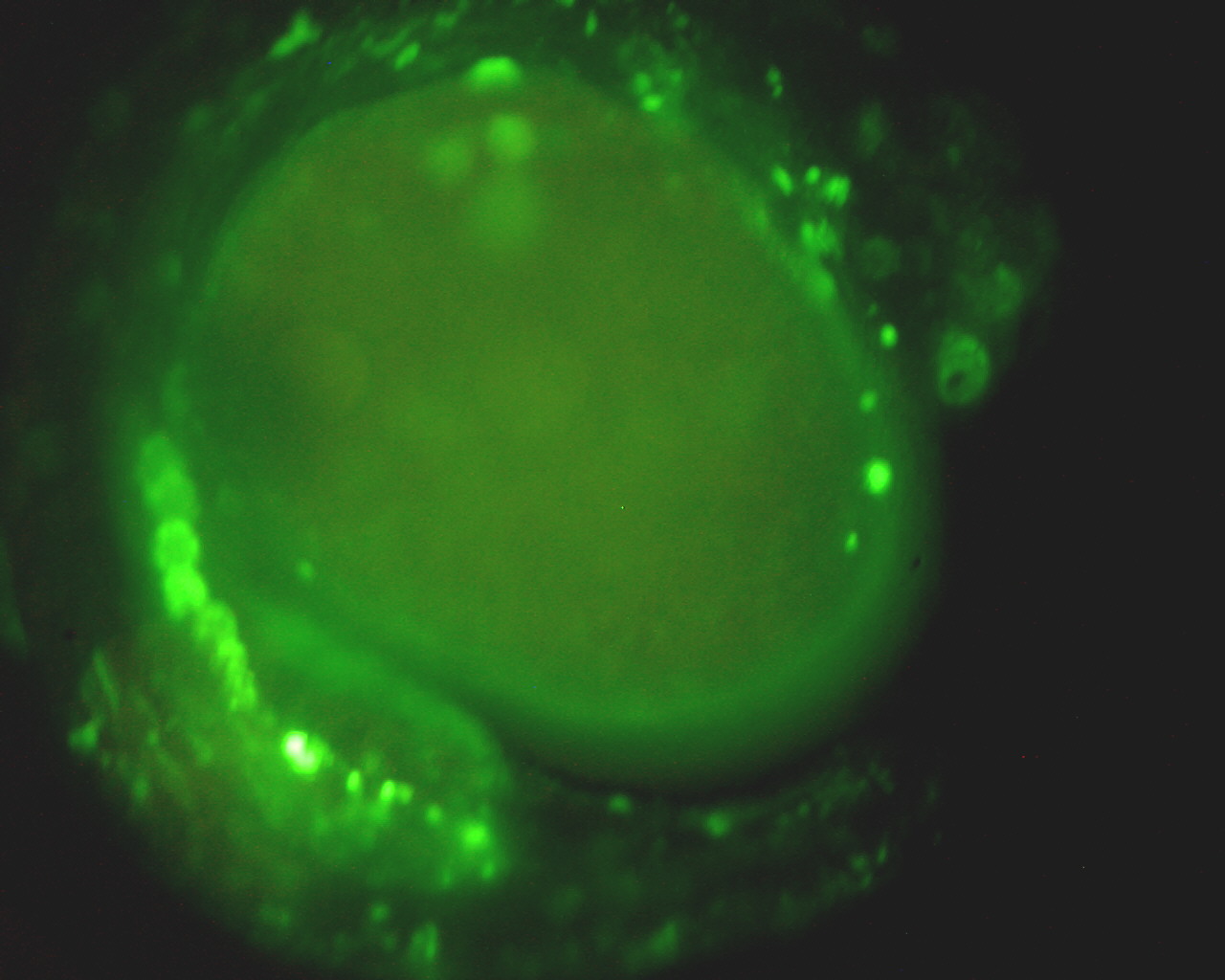

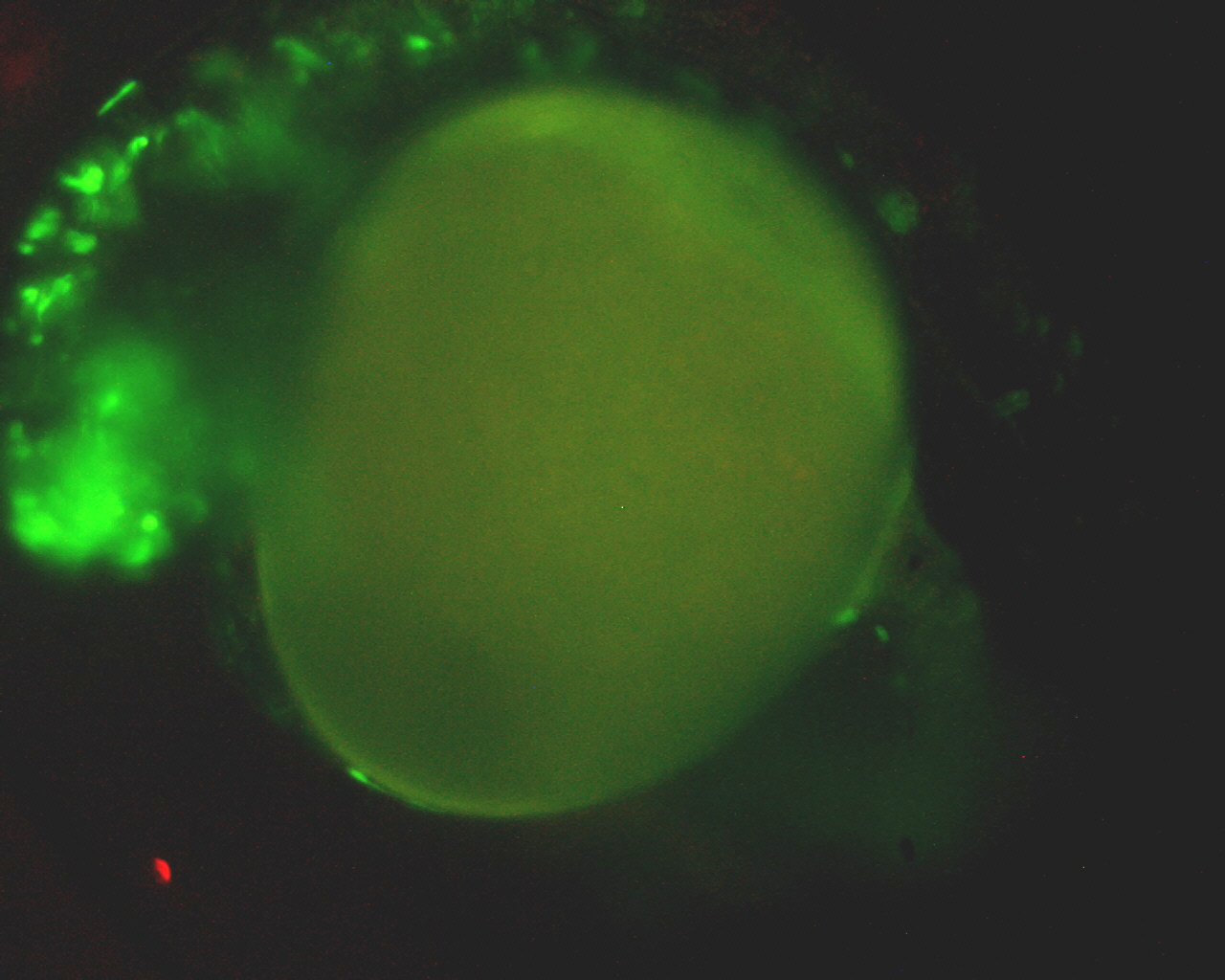

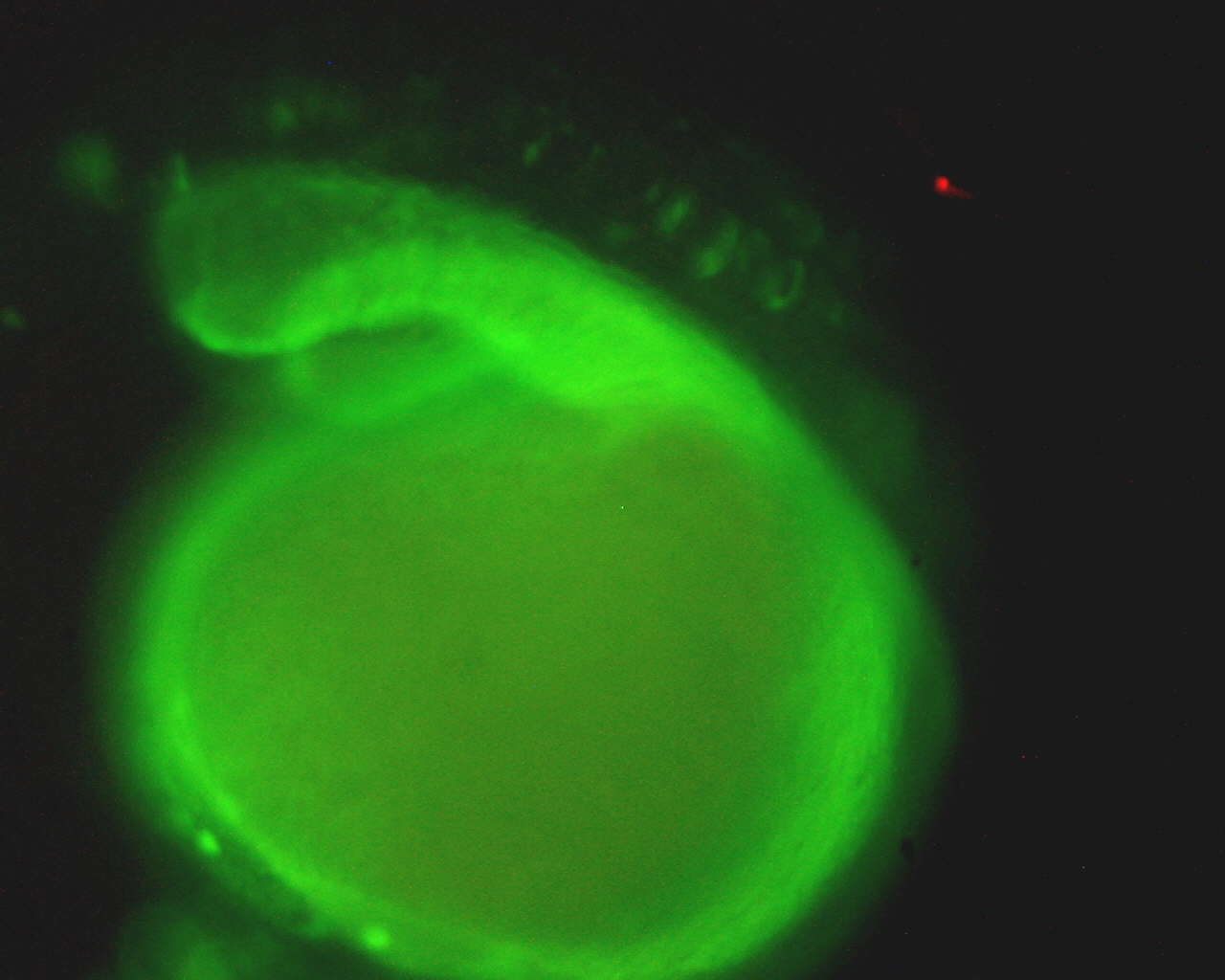


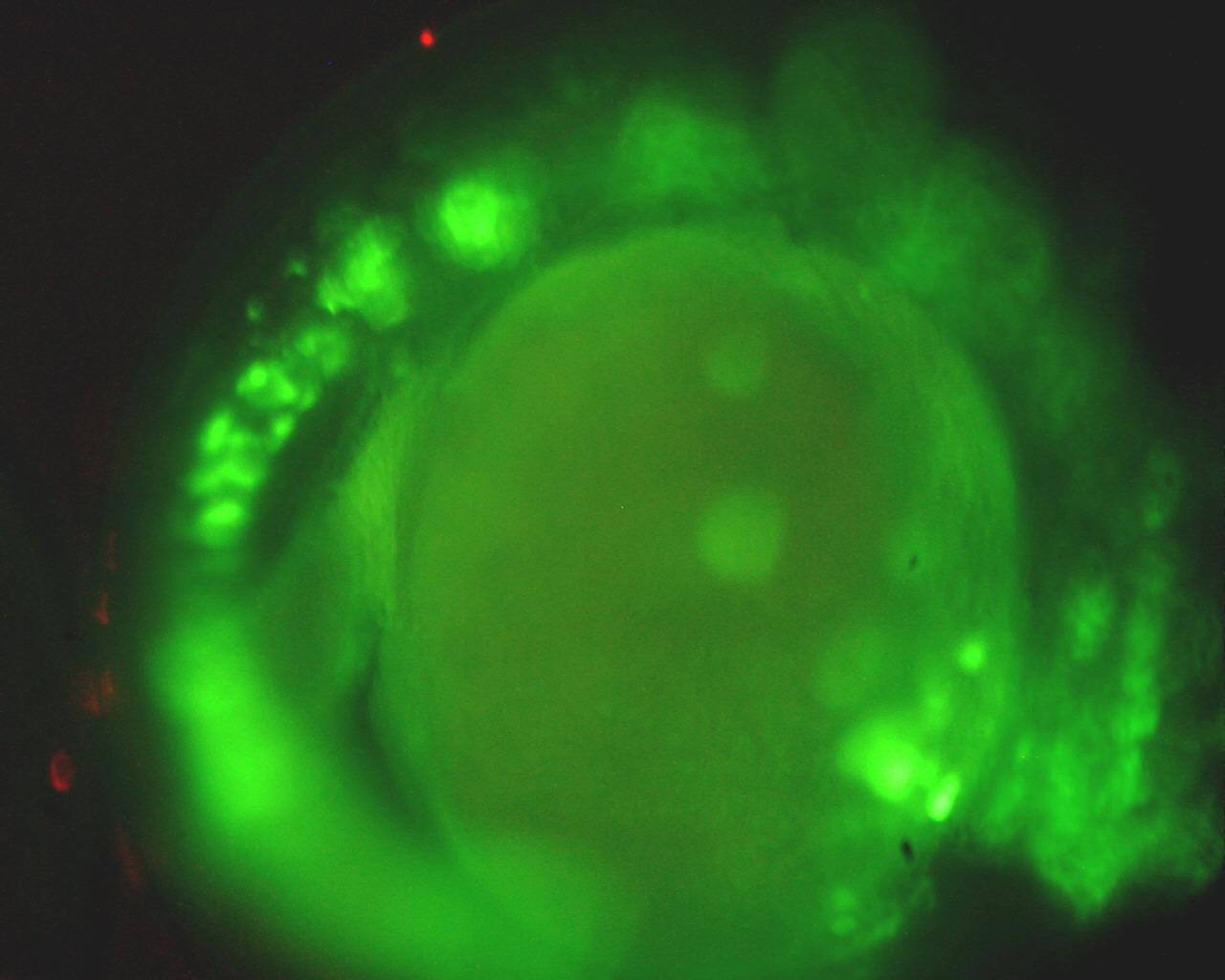

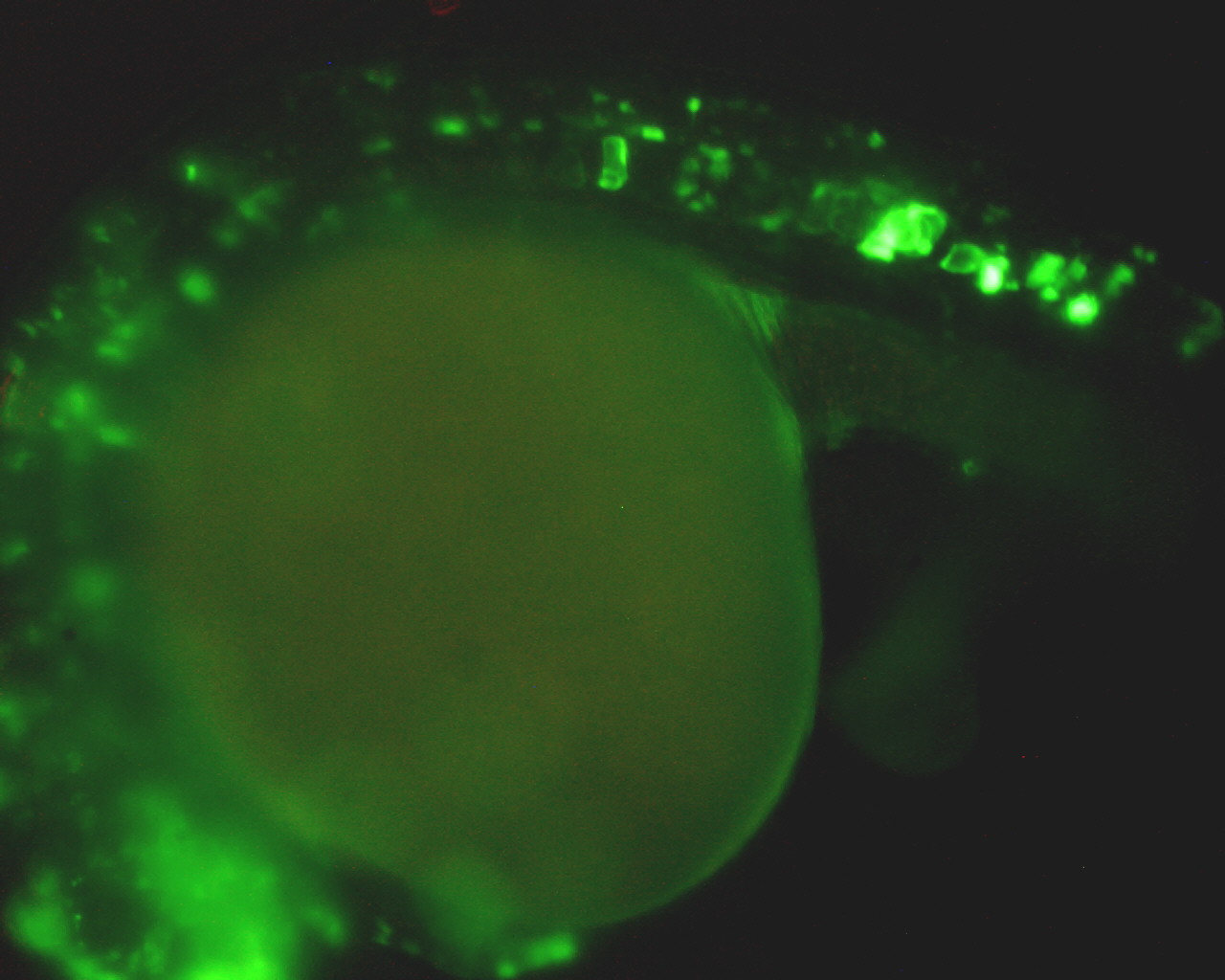

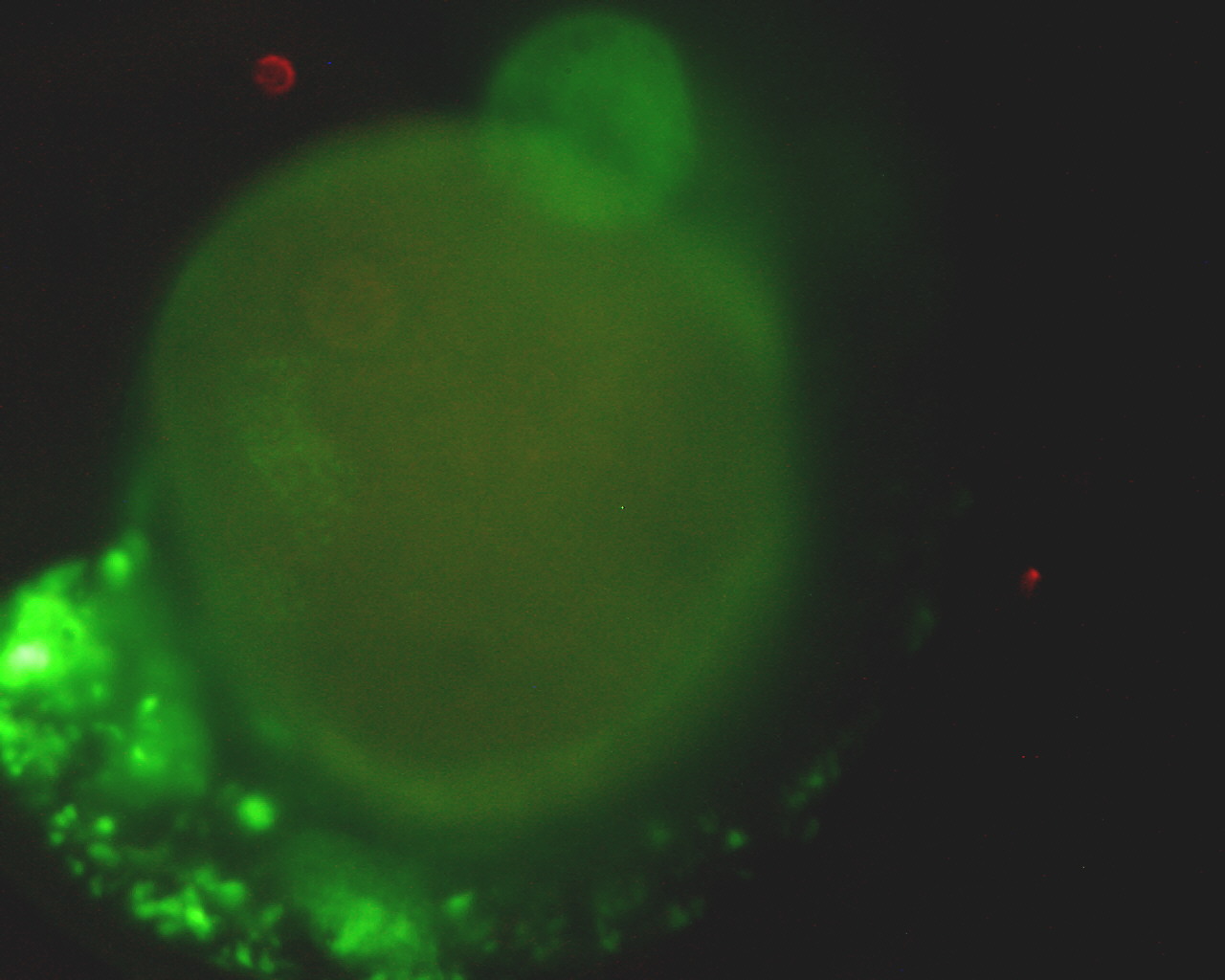

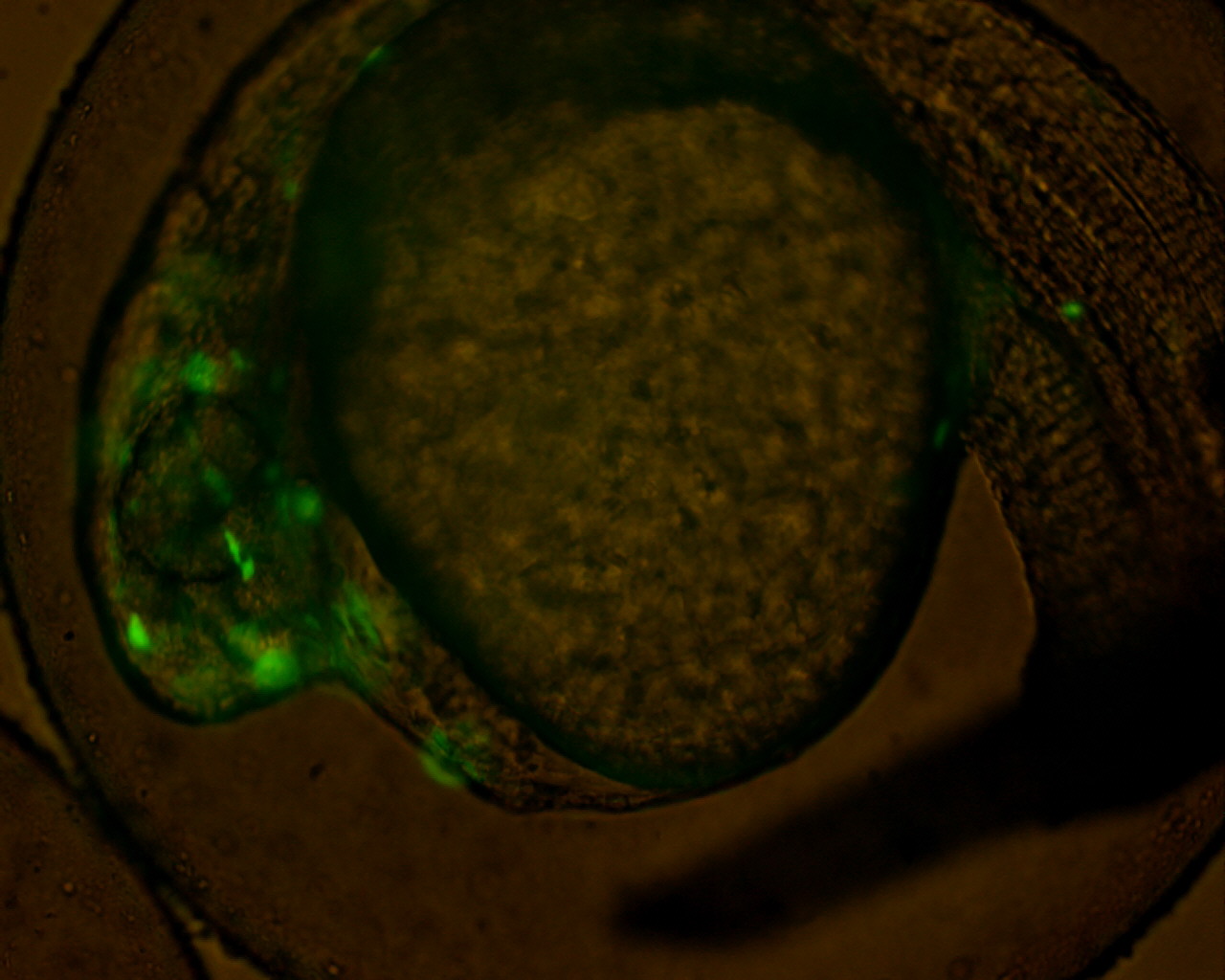

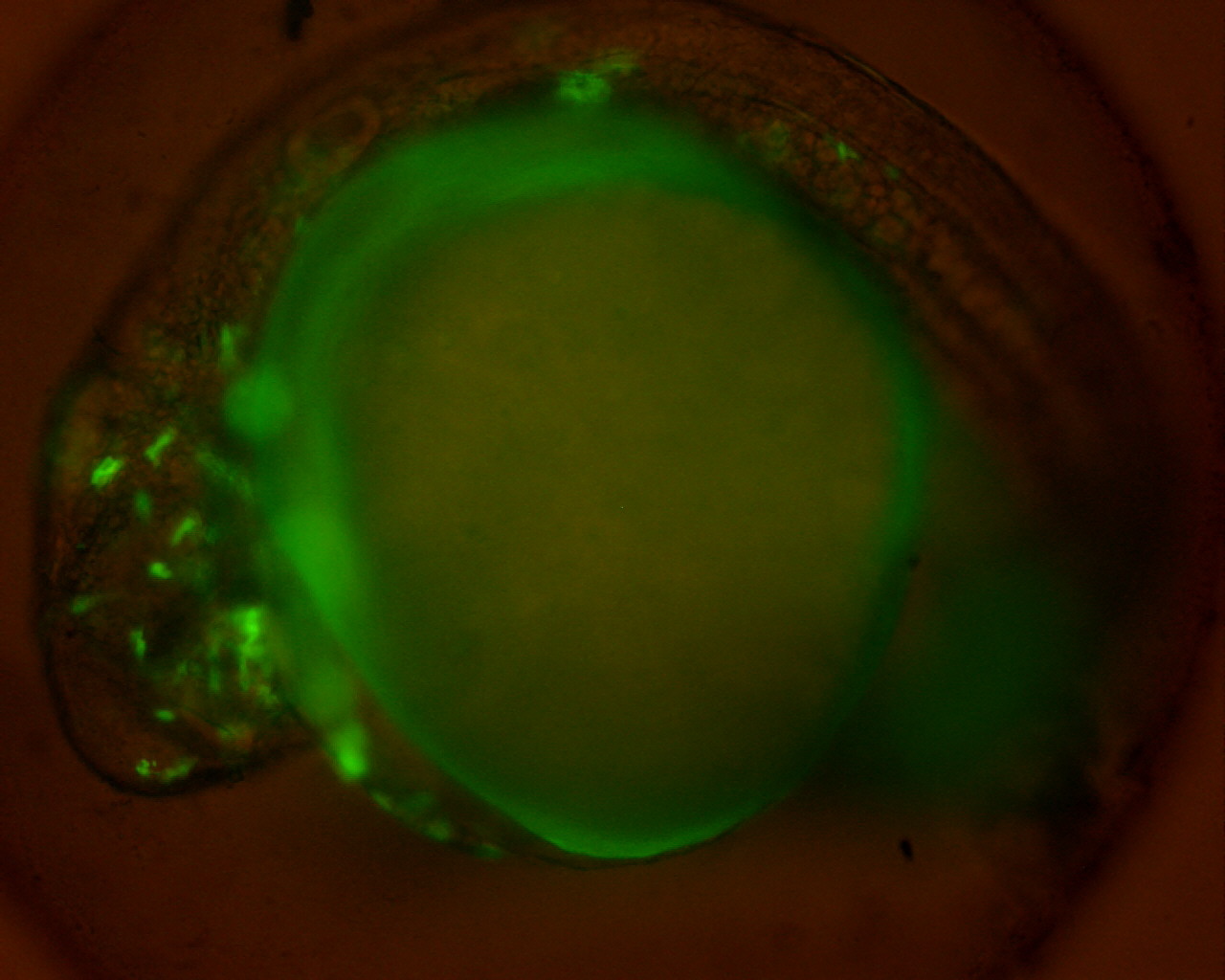

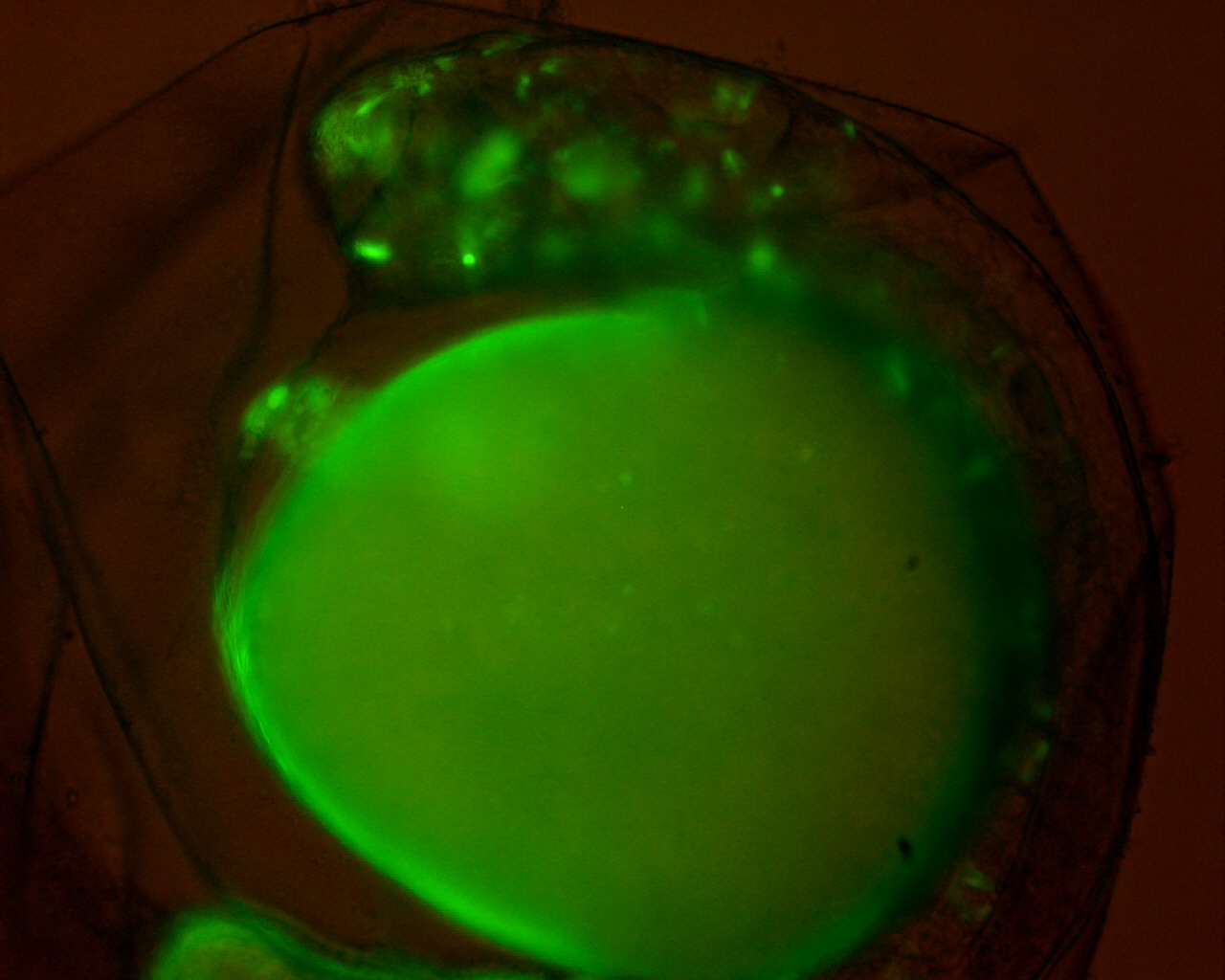

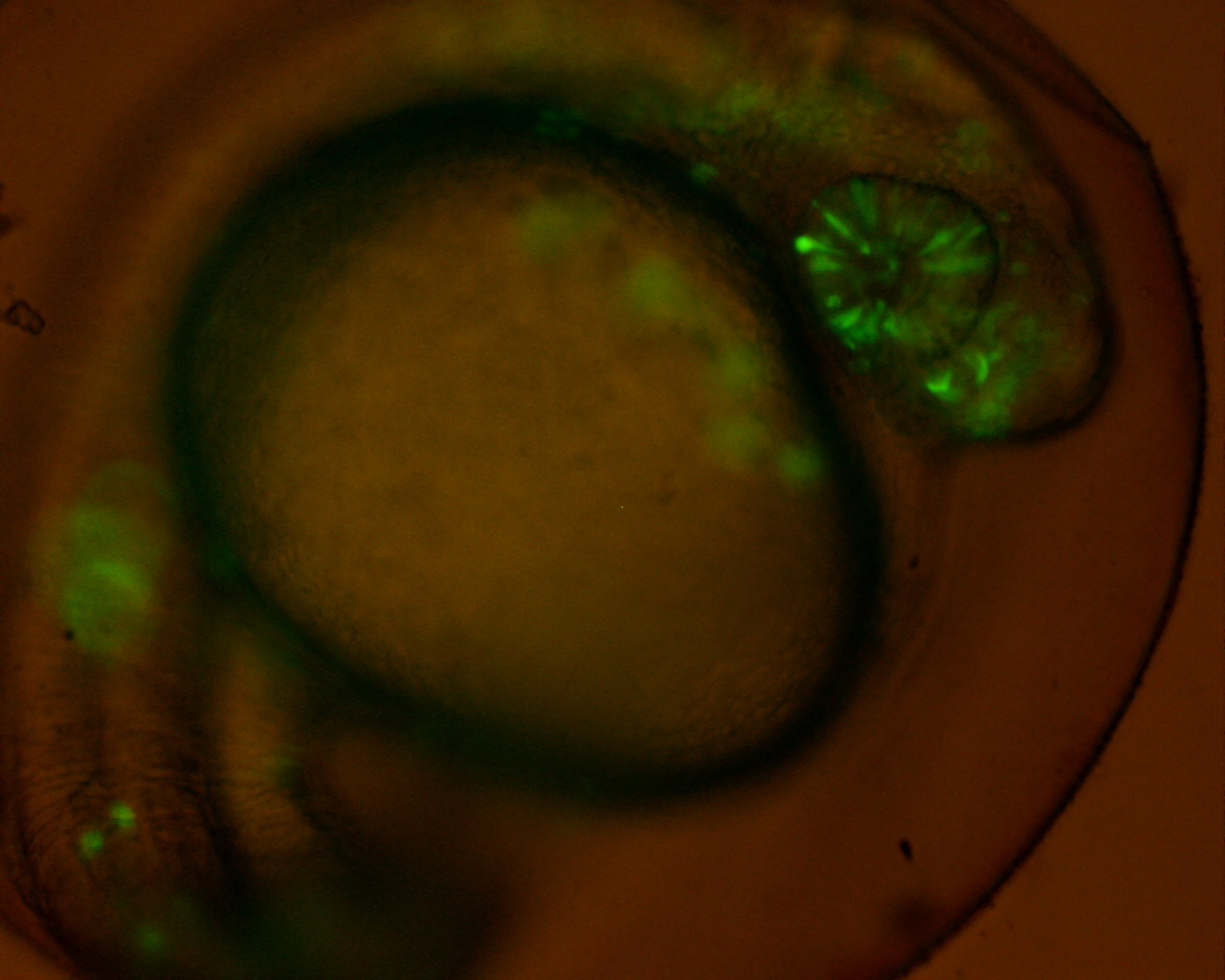

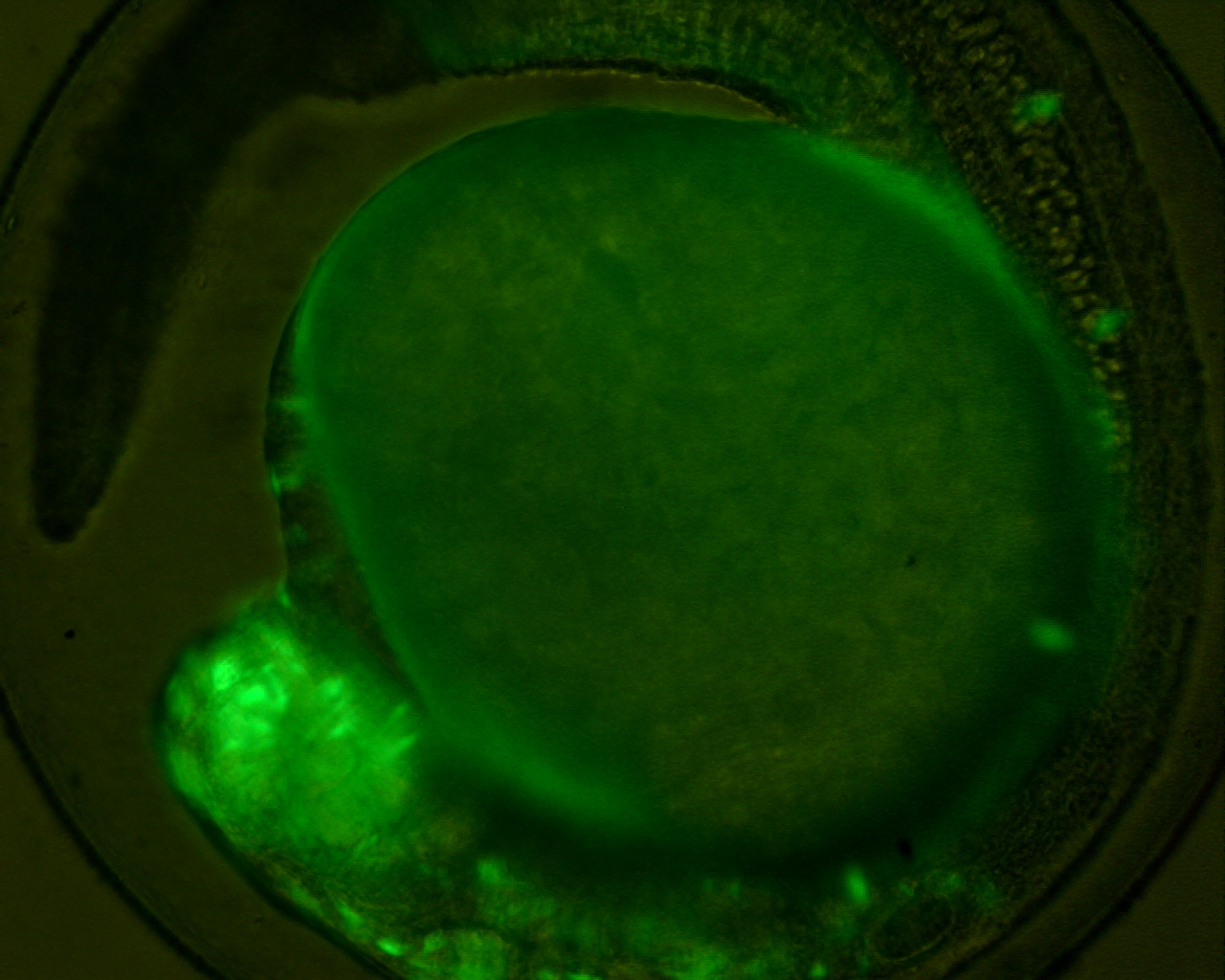

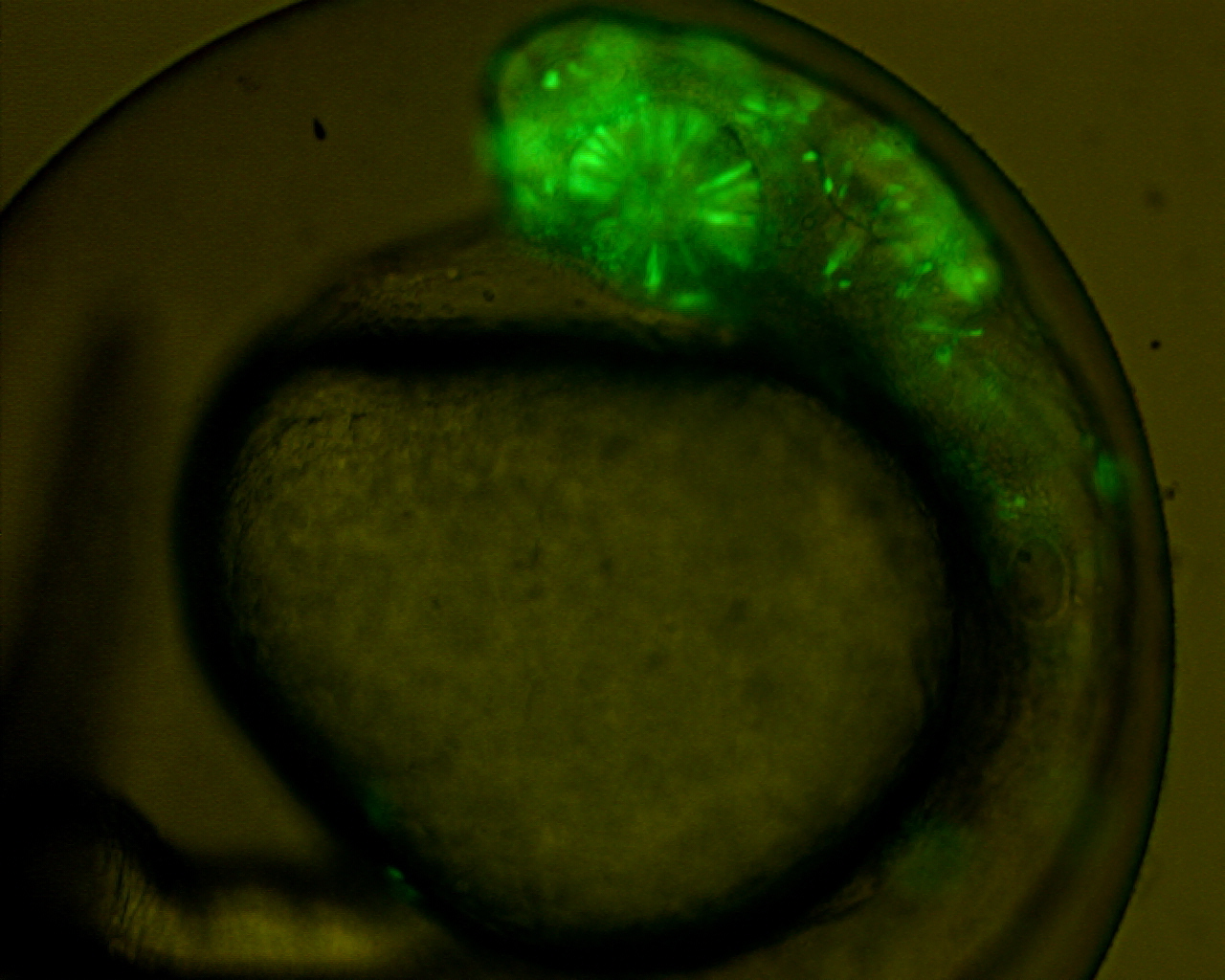


## 1060-Bp


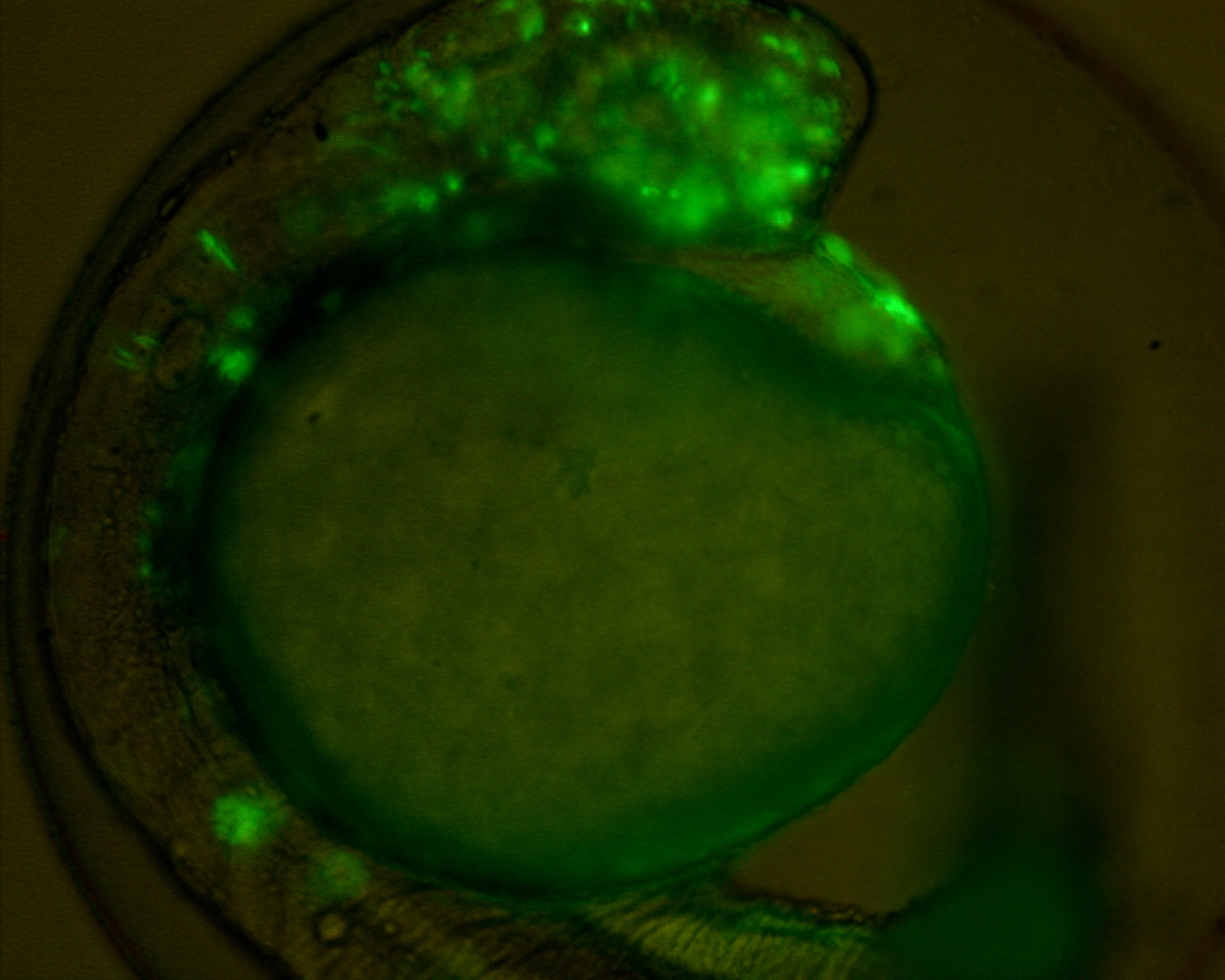

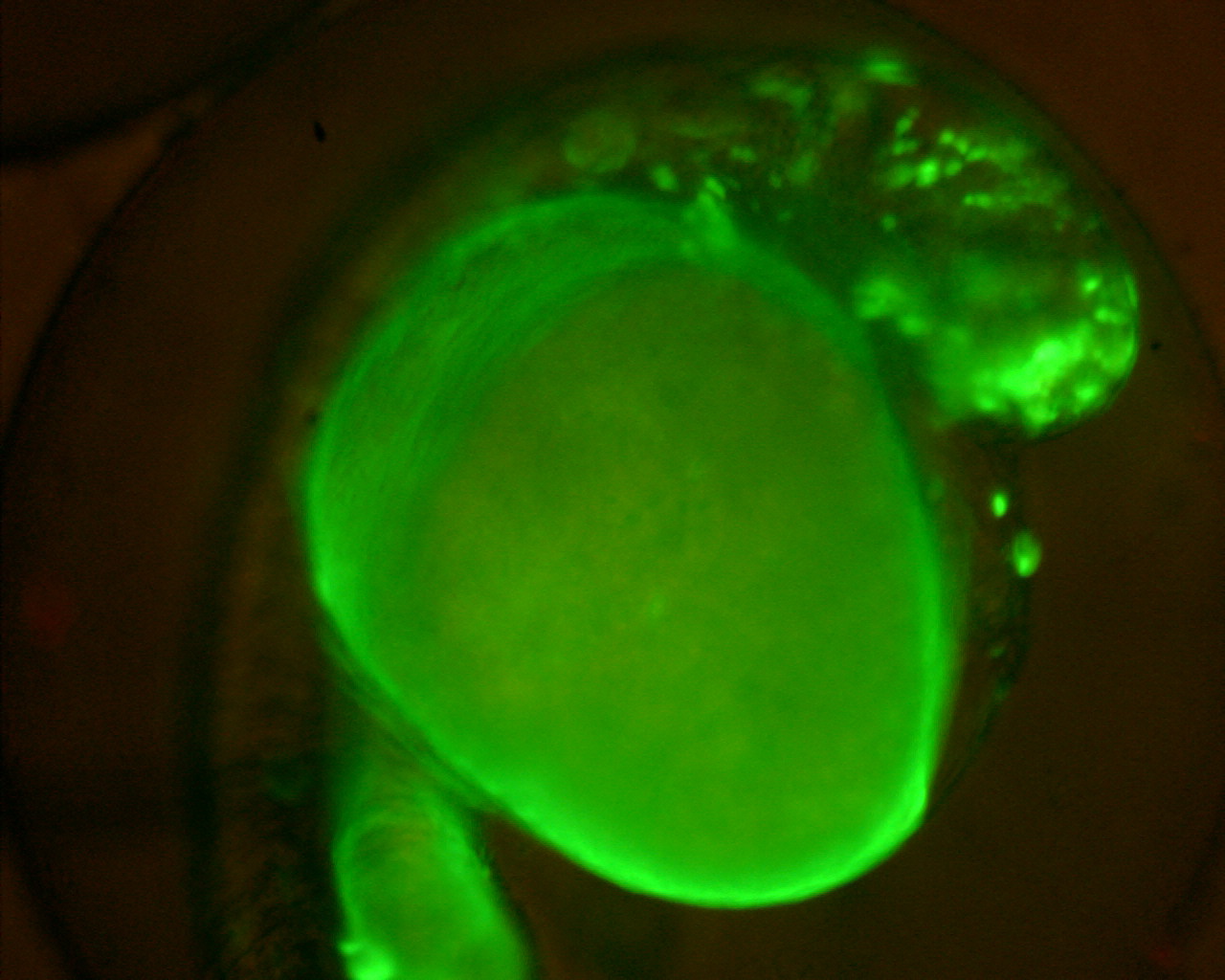

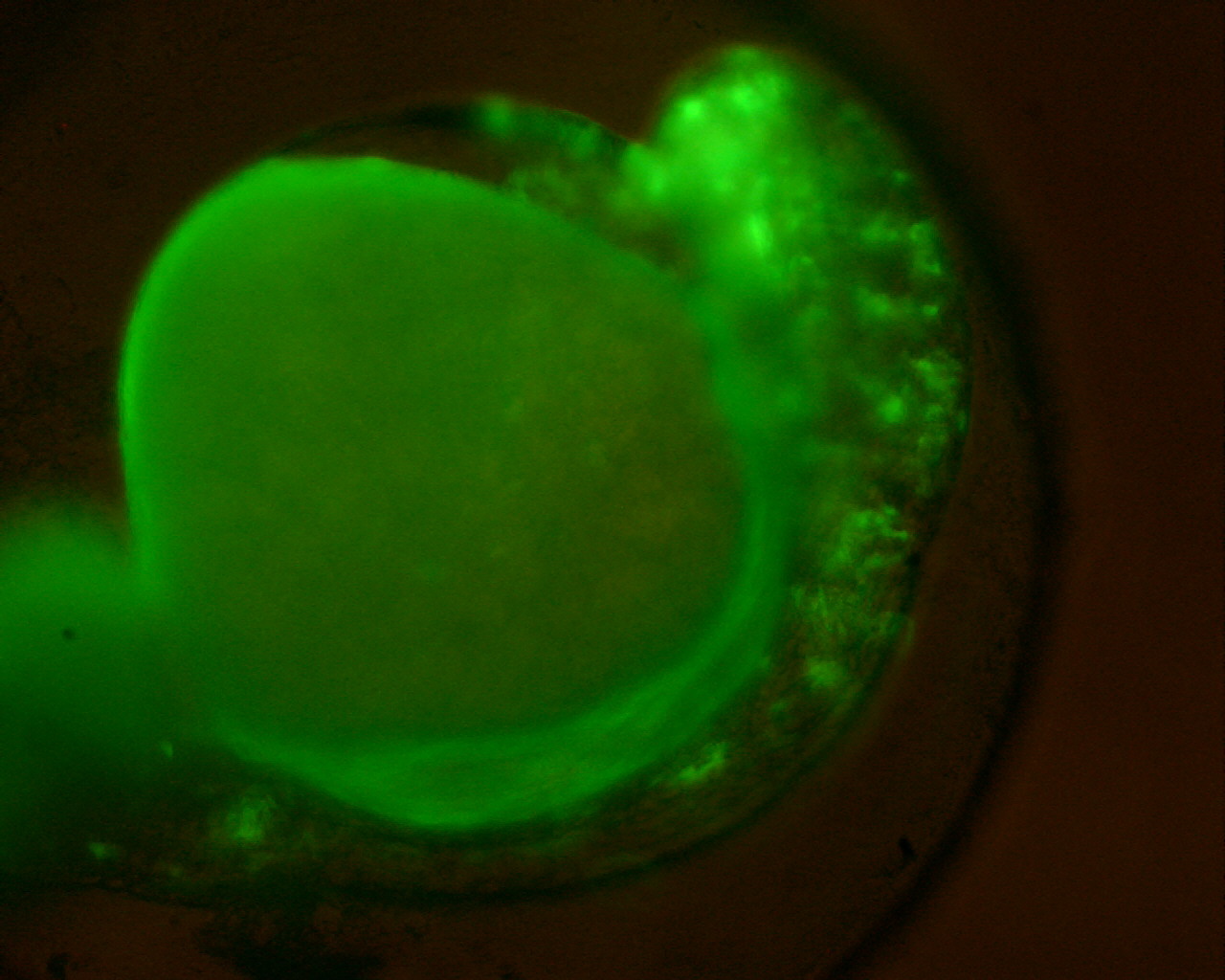


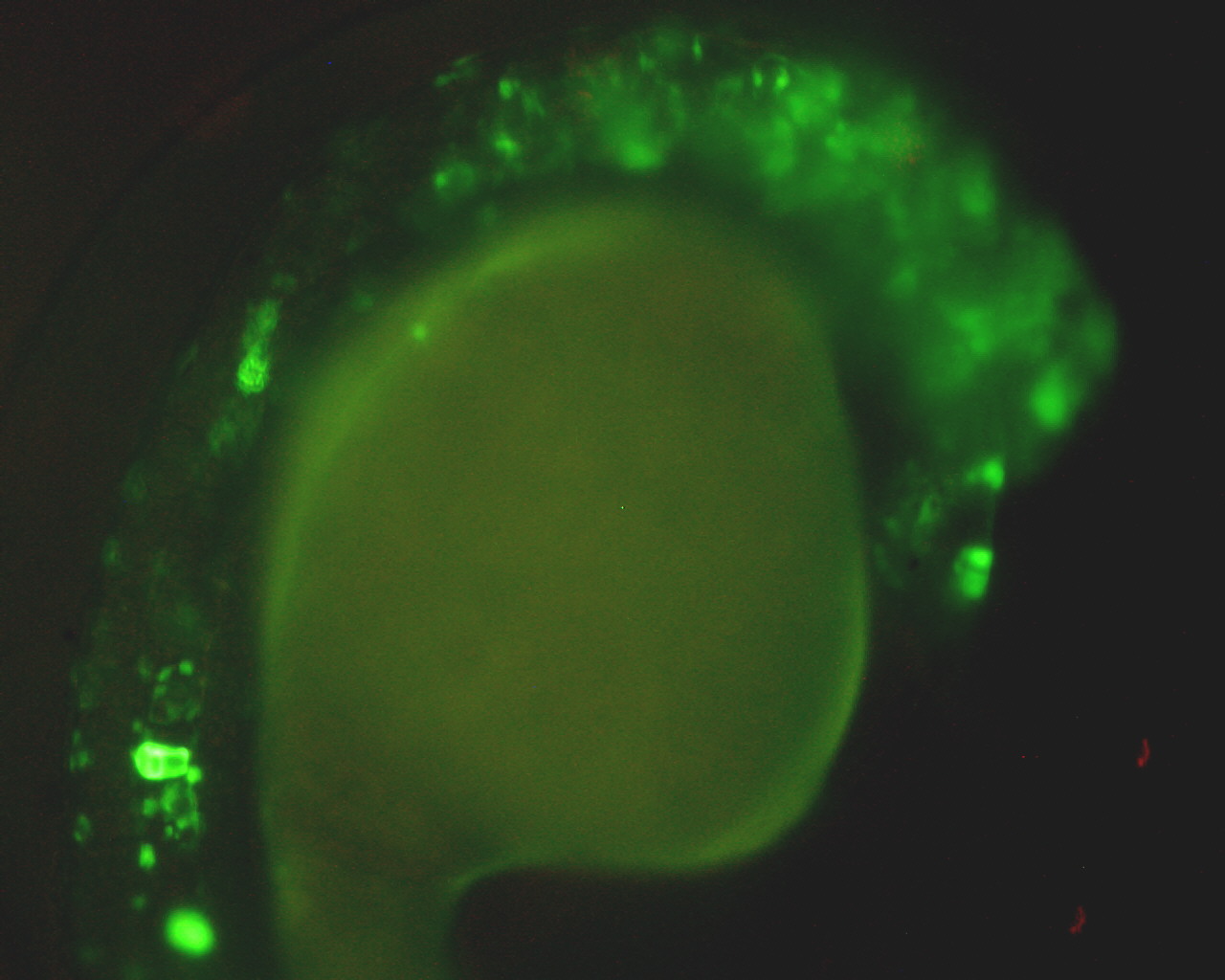

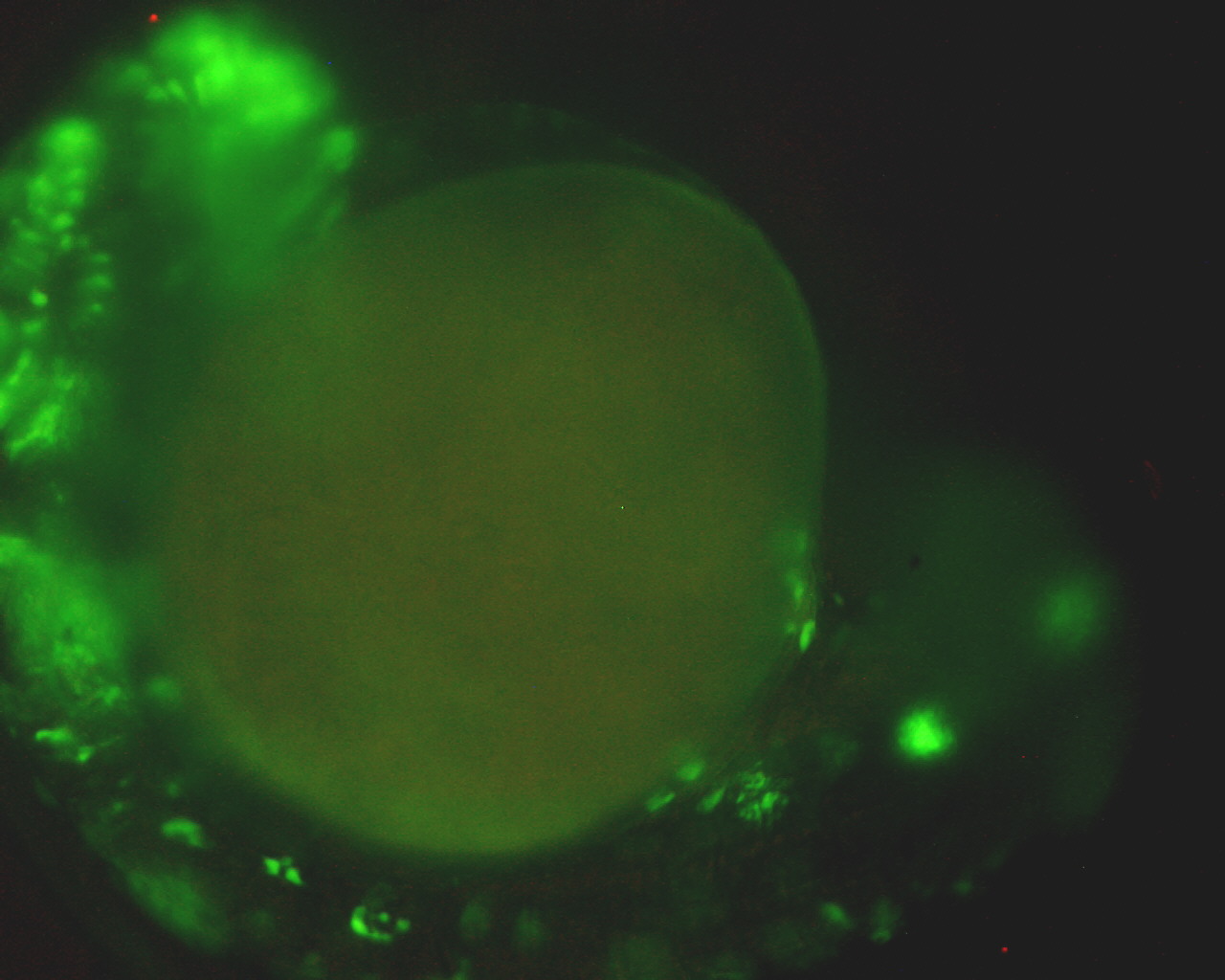

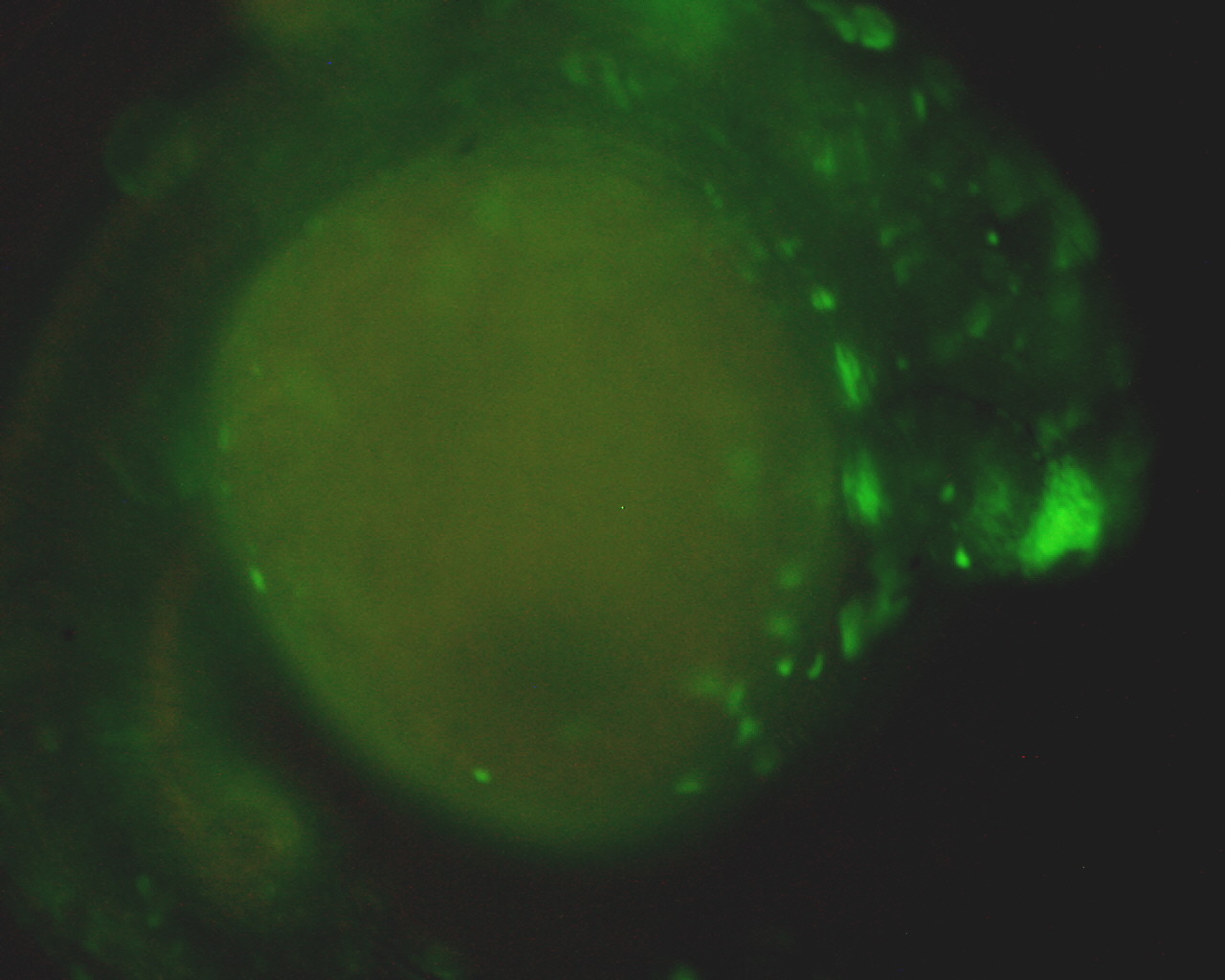

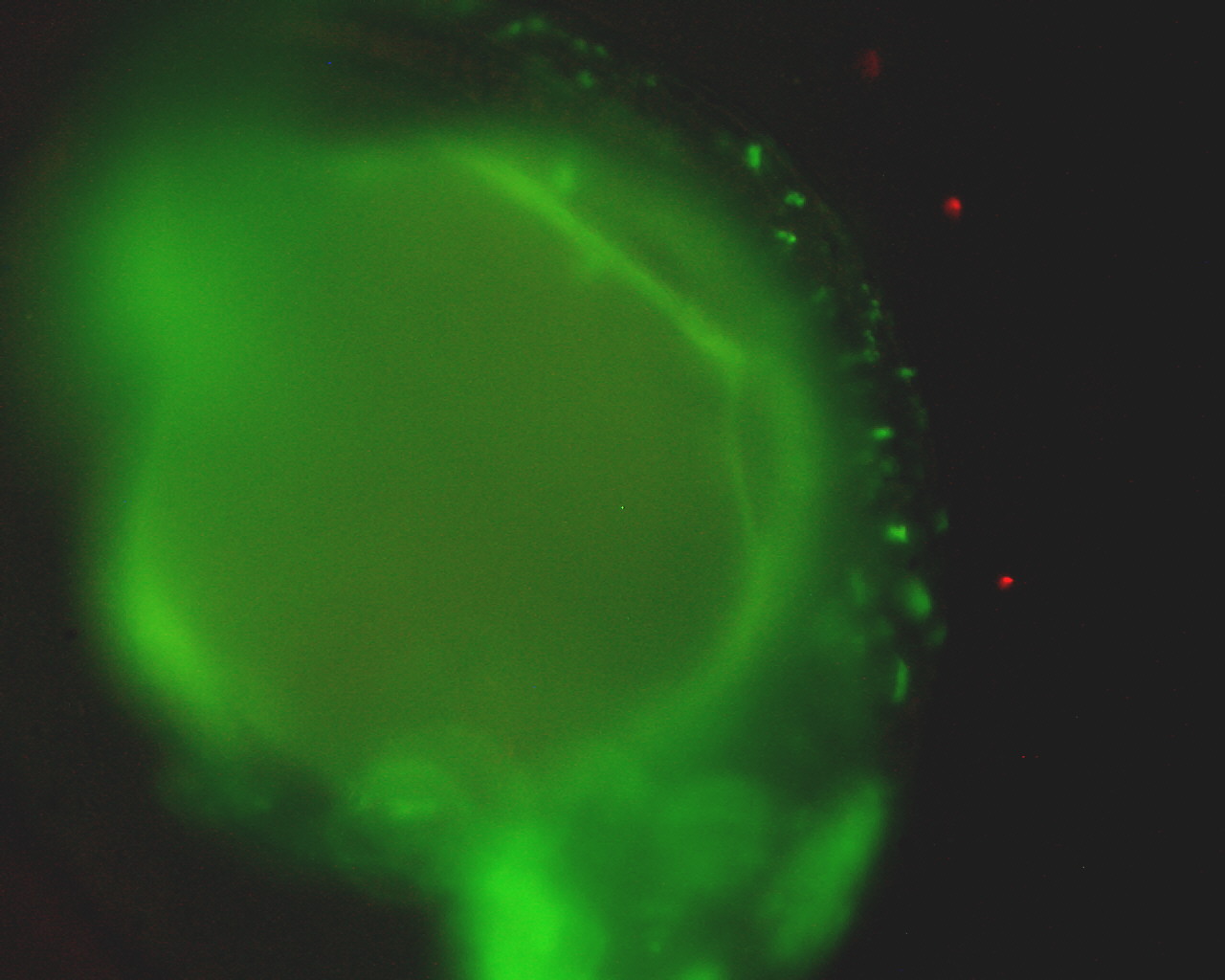

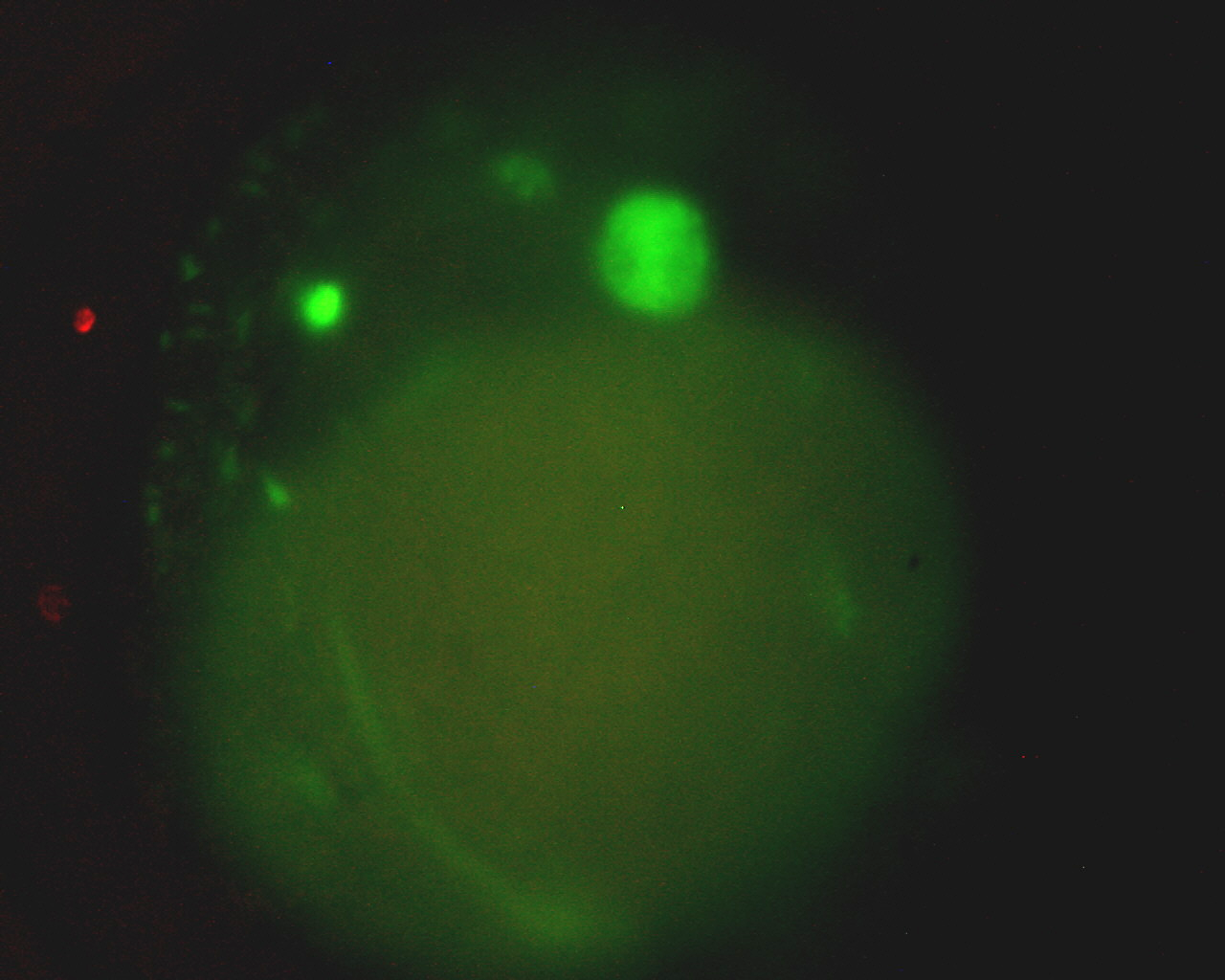

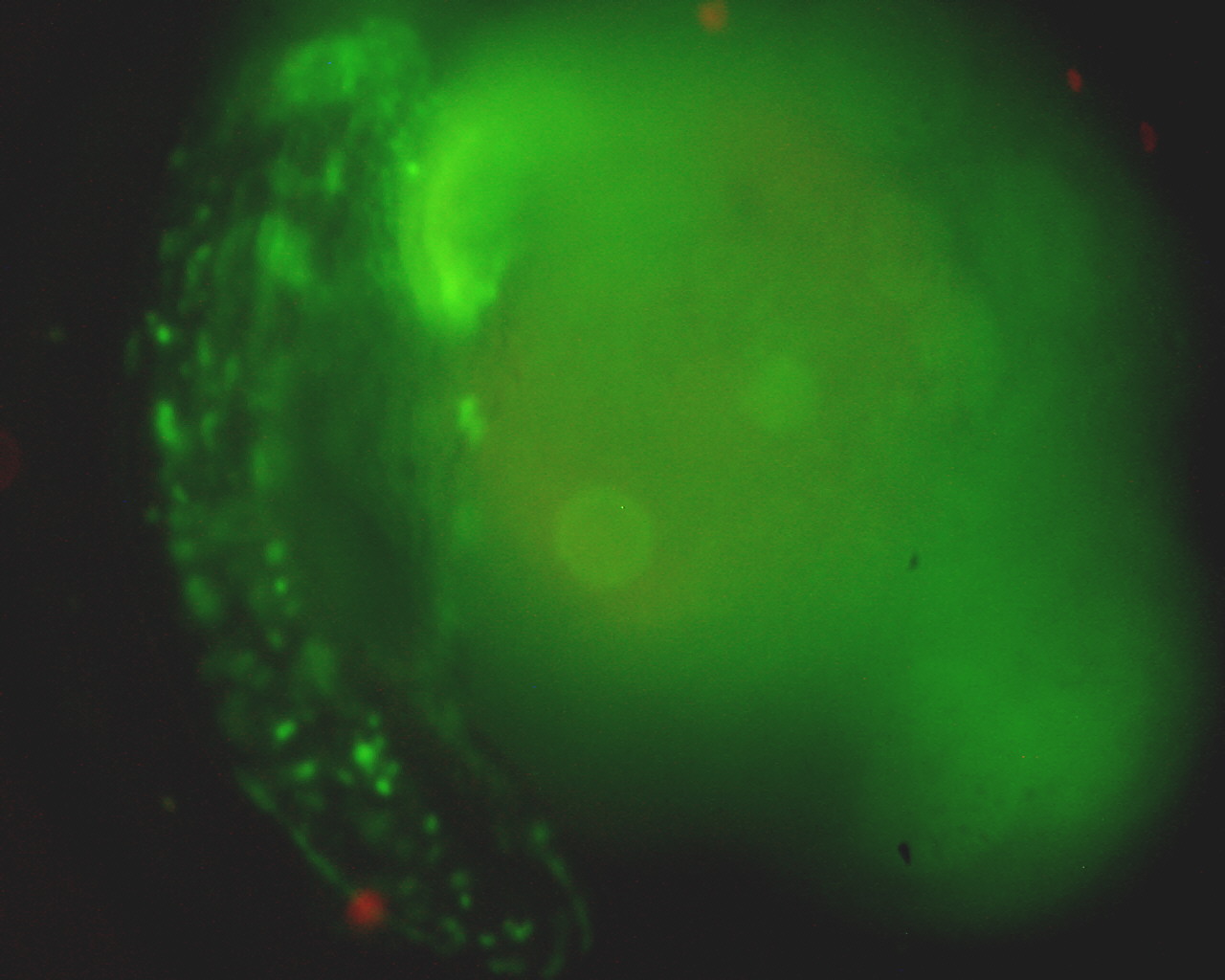


tail

Supplement: Additional file 6 — Additional images for 3087-Bp and 1060-Bp. More GFP images for 3087-Bp and 1060-Bp are shown in this file. [file 1471-213X-10-35-S6.DOC]
